# One-pot three-component synthesis and photophysical characteristics of novel triene merocyanines

**DOI:** 10.3762/bjoc.10.51

**Published:** 2014-03-05

**Authors:** Christian Muschelknautz, Robin Visse, Jan Nordmann, Thomas J J Müller

**Affiliations:** 1 Institut für Organische Chemie und Makromolekulare Chemie, Heinrich-Heine-Universität Düsseldorf, Universitätsstr. 1, D-40225 Düsseldorf, Germany

**Keywords:** alkynes, cross coupling, enamines, fluorescence, heterocycles, multicomponent reactions

## Abstract

Novel triene merocyanines, i.e. 1-styryleth-2-enylidene and 4-(1,3,3-trimethylindolin-2-ylidene)but-2-en-1-ylideneindolones are obtained in good to excellent yields in a consecutive three-component insertion Sonogashira coupling–addition sequence. The selectivity of either series is remarkable and has its origin in the stepwise character of the terminal addition step as shown by extensive computations on the DFT level. All merocyanines display intense absorption bands in solution and the film spectra indicate *J*-aggregation. While 1-styryleth-2-enylideneindolones show an intense deep red emission in films, 4-(1,3,3-trimethylindolin-2-ylidene)but-2-en-1-ylideneindolones are essentially nonemissive in films or in the solid state. TD-DFT computations rationalize the charge-transfer nature of the characteristic broad long-wavelength absorptions bands.

## Introduction

Functional organic materials [1], such as chromophores, fluorophores, and electrophores, constitute the active components in molecular electronics [2], photonics [3], and bioanalytics [4–6]. Among many chromophores the class of merocyanines [7–9], i.e. α-donor-ω-acceptor-substituted polyenes, has become increasingly interesting due to their fine-tunable electronic distribution [10]. For instance, merocyanines are perfectly suited for developing molecule-based non-linear optical materials and photovoltaic chromophores [11]. Classically, these push–pull chromophores always have been accessed by Knoevenagel condensations [12–14] or substitution reactions [15–18]. Still the quest for new synthetic strategies, novel substitution patterns, and eventually unusual properties and effects has become an ongoing challenge for organic synthesis, physical organic chemistry, and photophysics.

**Figure 1:**
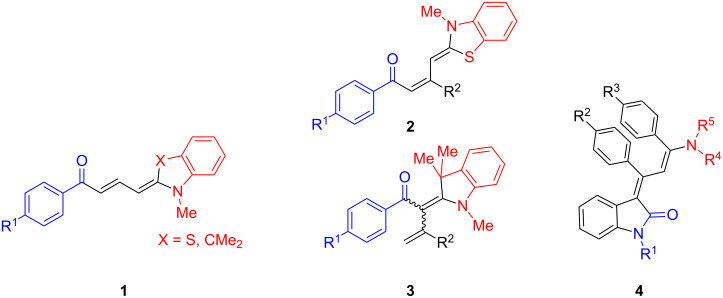
Linear push–pull solid-state diene lumophores with conformationally flexible and fixed acceptor moieties.

Inspired by the concept of a diversity-oriented synthetic approach to chromophores [19–26] we have launched a program to apply transition metal-catalyzed processes as an entry to consecutive multicomponent [27–28] and domino reactions [29]. These highly convergent strategies paved the way to luminescent push–pull dienes **1**–**4** with conformationally flexible and fixed acceptor units (Figure 1) [30–32], pyrazoles [33–34], benzodiazepines [35], furans and pyrroles [36–37] by consecutive multicomponent reactions and to highly emissive spirocycles [38–40] and pyranoindoles [41] via domino sequences. Interestingly, our versatile three-component enaminone synthesis [42–43] could be readily extended in a vinylogous fashion with enamines furnishing orange or deep red diene chromophores **2** and **3** that display aggregation induced emission [31].

In particular, the electrophilic enyne intermediate [32,40], which is trespassed in the three-component synthesis of solid-state luminescent push–pull indolones **4**, intrigued our interest for accessing even triene push–pull systems and to study their electronic properties. Here we report our findings on the diversity-oriented and highly selective three-component synthesis of a new class of deeply colored triene merocyanines in a one-pot fashion. Furthermore, their absorption and emission characteristics are investigated.

## Results and Discussion

**Scheme 1:**
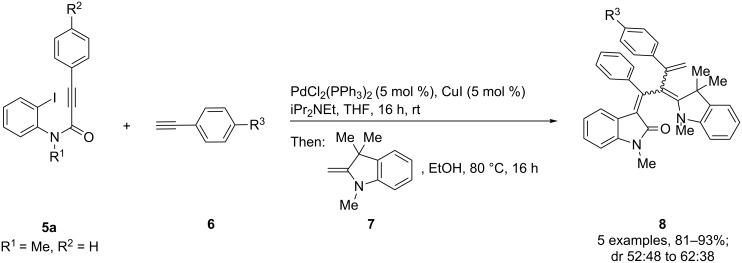
Three-component synthesis of 1-styryleth-2-enylideneindolones **8**.

After the coupling of the *N*-methyl-substituted alkynoyl *o*-iodoanilides **5a** and terminal arylalkynes **6** at room temperature under Sonogashira conditions forming ynylideneindolones as intermediates (the reaction was monitored by TLC to ensure complete conversion) [30–32], which were not isolated, an ethanolic solution of Fischer’s base (**7**) was added and reacted at reflux temperature to give 1-styryleth-2-enylideneindolones **8** in good to excellent yields as violet solids with a metallic luster (Scheme 1, Table 1). In contrary to the reaction with secondary amines, where the *E*,*E*-configured butadiene chromophores are formed with excellent stereoselectivity [30–32], Fischer’s base gives rise to the formation of a mixture of *E*,*E*- and *E*,*Z*-configured push–pull dienes with 3-styryl substituents in narrow diastereomeric ratios ranging from 52:48 to 62:38 (Table 1, entries 1–5) as indicated by the appearance of a second set of many signals in the proton and carbon NMR spectra.

**Table 1:** Three-component synthesis of 1-styryleth-2-enylidene indolones **8**.

Entry	Alkyne **6**	1-Styryleth-2-enylideneindolones **8**	Yield [%]^a^

1	R^3^ = H (**6a**)	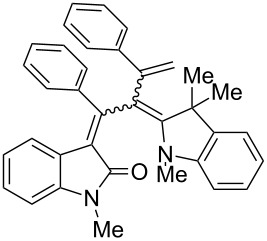 **8a**	93 (d.r. = 56:44)^b^
2	R^3^ = OMe (**6b**)	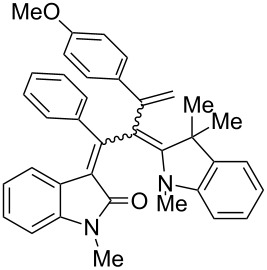 **8b**	81 (d.r. = 62:38)^b^
3	R^3^ = Cl (**6c**)	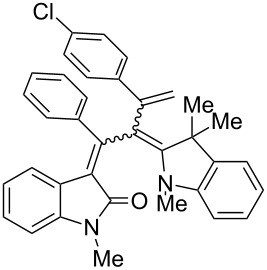 **8c**	82 (d.r. = 56:44)^b^
4	R^3^ = CN (**6d**)	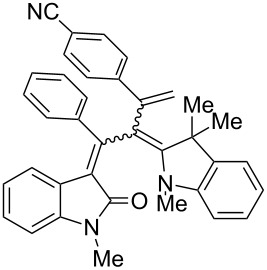 **8d**	87 (d.r. = 52:48)^b^
5	R^3^ = NO_2_ (**6e**)	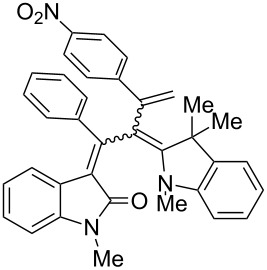 **8e**	91 (d.r. = 52:48)^b^

^a^The yields were determined after chromatography on silica gel. ^b^The diastereomeric ratios were determined by ^1^H NMR spectroscopy after chromatography on silica gel.

The structures of the 1-styryleth-2-enylideneindolones **8** were assigned by NMR, mass spectrometry and by combustion analysis. The diastereomeric ratios of the *E*,*E*- and *E*,*Z*-configured push–pull dienes **8** were determined by integration of the distinct signals of the corresponding pairs of geminal olefinic protons appearing at δ 4.8–5.2 and δ 5.5–5.8 for the major diastereomer and at δ 4.5–4.8 and δ 5.3–5.5 for the minor diastereomer in the ^1^H NMR spectra. The signals of the corresponding carbon nuclei are accordingly identified in the ^13^C NMR spectra as methylene signals at δ 114.7–117.5 for the major diastereomer and at δ 118.1–123.5 for the minor diastereomer.

**Figure 2:**
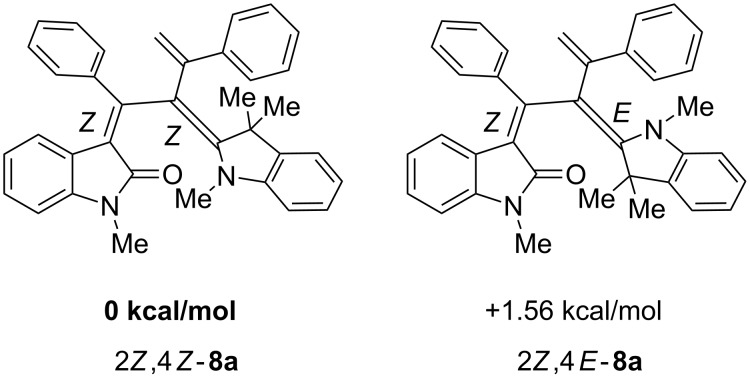
DFT-computed energy differences of the stereoisomers of 2*Z*,4*Z*-**8a** and 2*Z*,4*E*-**8a**.

Based upon computations (B3LYP functional, 6-31G* basis set) [44] on geometry-optimized diastereomers 2*Z*,4*Z*-**8a** and 2*Z*,4*E*-**8a** the former is energetically favored by 1.5 kcal mol^−1^ over the latter (Figure 2), nicely reproducing the experimentally determined very similar diastereomeric distribution of the 1-styryleth-2-enylideneindolones **8**.

**Scheme 2:**
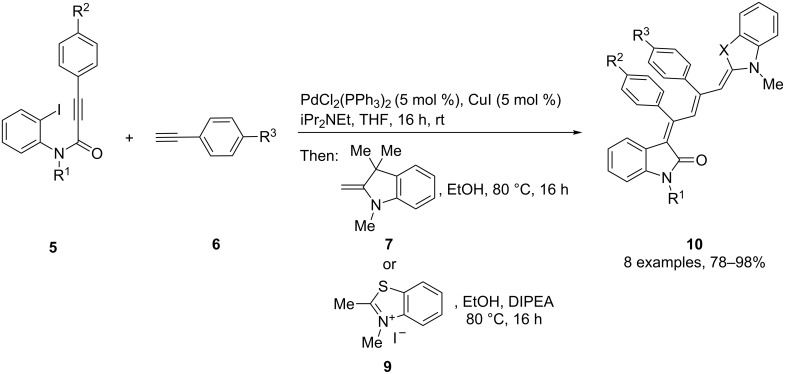
Three-component synthesis of 4-(1,3,3-trimethylindolin-2-ylidene)but-2-en-1-ylideneindolones **10**.

Interestingly, upon coupling of the *N*-tosyl-substituted alkynoyl *o*-iodoanilides **5b** and **5c** with terminal arylalkynes **6** at room temperature under Sonogashira conditions and reacting the ynylideneindolone intermediates (complete conversion monitored by TLC) [30–32] under the same conditions as above with an ethanolic solution of Fischer’s base (**7**), the 4-(1,3,3-trimethylindolin-2-ylidene)but-2-en-1-ylideneindolones **10a–g** (X = CMe_2_) were isolated in good to excellent yields as bluish-black solids with a metallic luster (Scheme 2, Table 2, entries 1–7). The sequence starting from the *N*-methyl-substituted alkynoyl *o*-iodoanilide **5a** surprisingly furnishes after coupling and reaction with an ethanolic solution of benzothiazolium iodide **9** in the presence of diisopropylethylamine (DIPEA) the 4-(3-methylbenzo[*d*]thiazol-2(3*H*)-ylidene)but-2-en-1-ylideneindolone **10h** (X = S) as a greenish-black solid and not the corresponding 1-styryleth-2-enylideneindolones **8** (Table 2, entry 8).

**Table 2:** Three-component synthesis of of 4-(1,3,3-trimethylindolin-2-ylidene)but-2-en-1-ylideneindolones **10**.

Entry	Alkynoyl *o*-iodoanilides **5**	Alkyne **6**	Enamine **7** or benzothiazolium salt **9**	4-(1,3,3-trimethylindolin-2-ylidene)but-2-en-1-ylideneindolones **10**	Yield [%]^a^

1	R^1^ = *p*Tos, R^2^ = H (**5b**)	**6a**	**7**	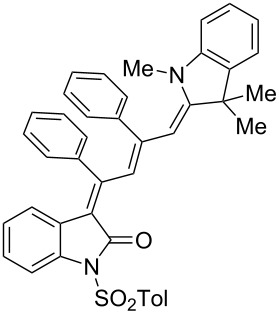 **10a**	98
2	**5b**	**6e**	**7**	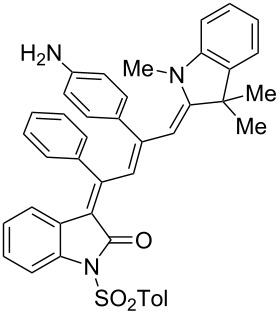 **10b**	90^b^
3	**5b**	**6c**	**7**	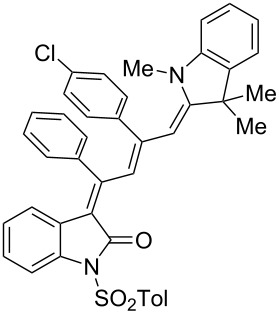 **10c**	78
4	**5b**	**6d**	**7**	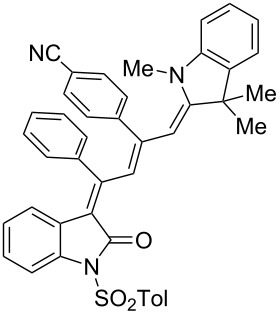 **10d**	82
5	R^1^ = *p*Tos, R^2^ = Cl (**5c**)	R^3^ = *t*-Bu (**6e**)	**7**	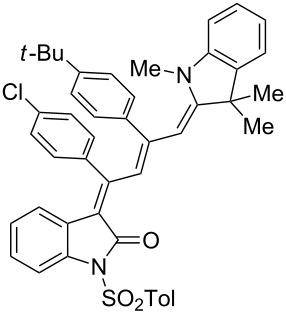 **10e**	82
6	**5c**	**6c**	**7**	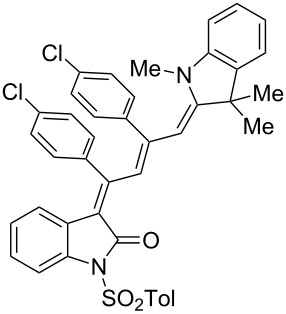 **10f**	92
7	**5c**	**6d**	**7**	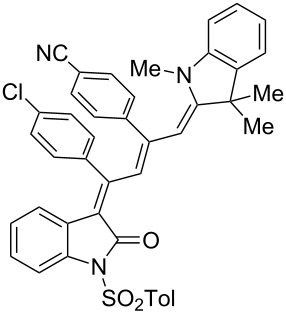 **10g**	90
8	**5a**	**6c**	**9**^c^	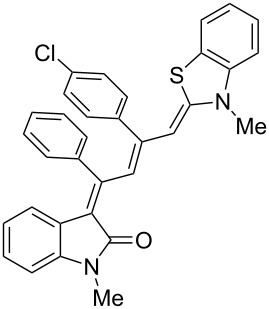 **10h**	84

^a^The yields were determined after chromatography on silica gel. ^b^The nitro group was reduced to an amino group under the reaction conditions. ^c^Diisopropylethylamine was added for in situ generation of the *S*,*N*-ketene acetal.

**Figure 3:**
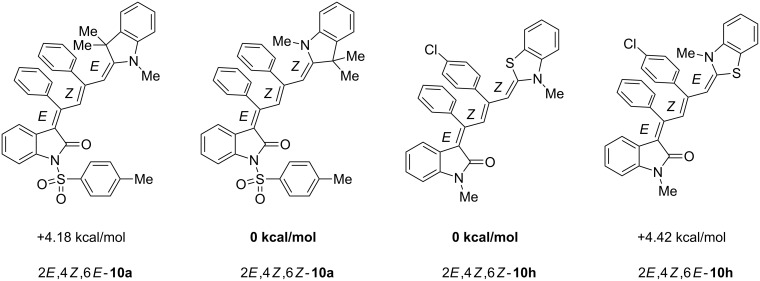
DFT-computed energy differences of the stereoisomers of **10a** and **10h**.

The structures of the 4-(1,3,3-trimethylindolin-2-ylidene)but-2-en-1-ylideneindolones **10** were unambiguously assigned by NMR, mass spectrometry and by combustion analysis. The appearance of only a single set of signals in the NMR spectra indicates that the process is highly stereoselective. The occurrence of deep-colored products indicates the presence of a chromophore with extended π-electron conjugation where the terminal acceptor and donor functionalities are connected via an essentially coplanar methine bridge. Based upon analogy to the 4-aminoprop-3-enylideneindolones [30–32] and computations (B3LYP functional, 6-31G* basis set) [44] on geometry-optimized 2*E*,4*Z*,6*Z*- and 2*E*,4*Z*,6*E*-diastereomers of **10a** and **10h** the stereochemistry of the most stable isomers of these novel triene merocyanines was assigned to be 2*E*,4*Z*,6*Z* for both the Fischer’s base derivatives **10a–g** (X = CMe_2_) and the benzothiazole derivative **10h** (X = S) (Figure 3). Since the stereoconvergent-product formation (only single sets of signals are obtained in the NMR spectra for all representatives of **10**) occurs at elevated temperatures (boiling ethanol in the terminal step) it can be assumed that the assigned structures represent the thermodynamically and kinetically controlled products in this series.

**Scheme 3:**
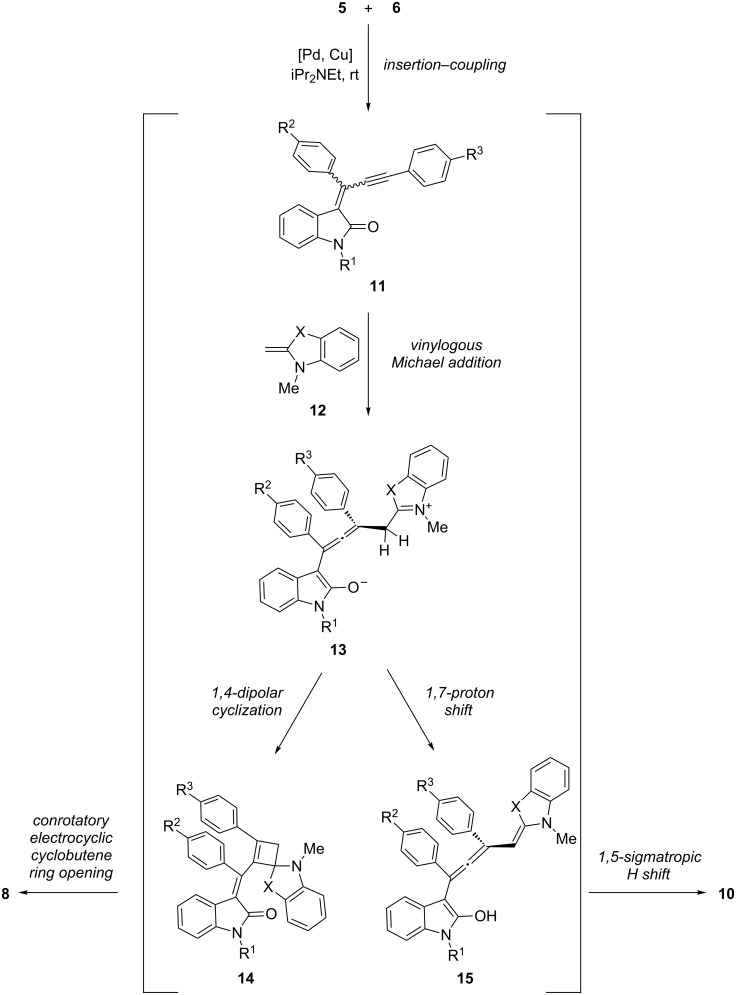
Mechanistic rationale of the three-component sequence furnishing the 1-styryleth-2-enylideneindolones **8** and 4-(1,3,3-trimethylindolin-2-ylidene)but-2-en-1-ylideneindolones **10**.

Mechanistically the observed unusual selectivity for the formation of 1-styryleth-2-enylideneindolones **8** vs 4-(1,3,3-trimethylindolin-2-ylidene)but-2-en-1-ylideneindolones **10** obviously originates from minute electronic differences in the ynylideneindolone intermediate **11**. This species was previously isolated and unambiguously structurally identified [40]. In the case of amine additions to ynylideneindolone intermediates **11** we recently could show by experimental and computational studies that the terminal Michael addition proceeds in a stepwise fashion with the intermediacy of an allenyl enol that undergoes a rapid, irreversible 1,5-sigmatropic hydride shift triggering the allenyl enol–dienone tautomerism [30]. Therefore, we propose a similar mechanism for the formation of the merocyanines **8** and **10** (Scheme 3). The sequences commence after oxidative addition of the Pd species in the carbon–iodine bond of **5** with a 5-*exo*-*dig* insertion of the appended alkynoyl moiety, which is coupled by transmetallation with the alkyne **6** and reductive elimination to give the ynylideneindolone intermediate **11**. The ynylideneindolone **11** is a vinylogous Michael system, and therefore, it is reasonable to assume a 1,4-addition of the nucleophilic enamine **12**, which is employed directly (as in the case of Fischer’s base (**7**)) or generated in situ by deprotonation of the benzothiazolium salt **9**. Hence, in both cases a resonance-stabilized iminium–allenyl enolate **13** is formed. Here, the bifurcation of the sequences takes over. Based upon product analysis the pathway to the formation of the merocyanines **8** begins with a 1,4-dipolar cyclization of **13** furnishing the highly substituted cyclobutene intermediate **14**. Finally, the conrotatory electrocyclic ring opening of the cyclobutene occurs under thermodynamic control, which is obviously only governed by steric effects as reflected by very similar levels of diastereoselectivity of the double-bond formation. In contrast the formation of the merocyanines **10** starts with a proton transfer from the CH-acidic α-position of the iminium moiety of **13** to the amide enolate part. The resulting enol **15** is part of an allenyl enol, which is just perfectly suited for undergoing a 1,5-sigmatropic H-shift, giving directly rise to the formation of the conjugated push–pull triene **10**.

This mechanistic rationale suggests that the observed remarkable chemoselectivity in the formation of two different triene merocyanines could originate from minute electronic distributions in the zwitterionic key intermediate, which is controlled in the allenyl enolate moiety by the indolyl nitrogen substituent R^1^ and by the fragment X on the iminium part, which participates in the stabilization on that side of the zwitterion. The former hypothesis is supported by the fact that only methyl-substituted ynylideneindolone intermediates **11** enable the cyclobutene pathway, whereas all *N*-tosyl derivatives exclusively give the merocyanines **10** via the allenyl enol pathway. All other remote substituents on the ynylideneindolone intermediates **11** do not influence the outcome of the sequence. The latter hypothesis of the iminium-ion stabilization obviously influences the lifetime of the zwitterion **13**. Furthermore the enamine formed by deprotonation of **9** is a *S*,*N*-ketene acetal, which is significantly more nucleophilic than Fischer’s base (**7**). The higher reactivity of the latter enamine and the higher thermodynamic stability of the iminium intermediate both account for a stepwise pathway that proceeds via the intermediacy of zwitterion **13**. Therefore, if intermediate **13** is long lived enough to undergo the prototropy the merocyanines **10** will be the obvious product. Therefore, the local charge density in **13** is most crucial for the bifurcation and, hence, for the product formation.

For scrutinizing this rationale we computed the electrostatic charges on the atoms 1–7 of the allenyl enolate moiety of model zwitterions **16**, which only differ by the *N*-substituent, on the density functional level of theory (B3LYP functional, 6-31G* basis set) [40]. For a stepwise cyclobutene formation via 1,4-dipolar cyclization the negative partial charge on the central allenyl carbon atom 6 should be relatively high. The computations of the electrostatic charges for an *N*-methyl (**16a**) and an *N*-tosyl intermediate (**16b**) clearly shows charge density differences at five distinct atoms (Table 3).

**Table 3:** Selected computed electrostatic charges of zwitterion intermediates **16** (DFT computations with B3LYP functional and the 6-31G* basis set).

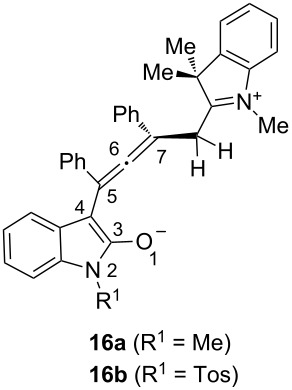		

Atom	**16a** (R^1^ = Me)	**16b** (R^1^ = *p*Tos)

O1	−0.564	−0.520
N2	−0.107	−0.299
C3	0.480	0.416
C4	−0.296	−0.209
C5	0.035	−0.060
C6	−0.162	−0.119
C7	−0.070	−0.089

All merocyanines **8** and **10** are expectedly deeply colored both in the solid state and in solution. For further investigation of the photophysical properties the absorption spectra were recorded in dichloromethane solution and of thin films prepared by dropcasting of concentrated dichloromethane solutions on glass-probe cuvettes (Table 4). For the powders and films of the merocyanines **8** intense deep-red luminescence was detected upon eyesight. The emission spectra of the films of **8** were recorded, whereas the films of the merocyanines **10** did not display emission upon eyesight.

**Table 4:** Selected absorption and emission data of the 1-styryleth-2-enylidene indolones **8** and the 4-(1,3,3-trimethylindolin-2-ylidene)but-2-en-1-ylidene indolones **10**.

Entry	Compound	Absorption		Emission	Stokes shift

		λ_max,abs_ [nm] (ε, L·mol^−1^·cm^−1^)^a^	λ_max,abs_ [nm] (film)^b^	λ_max,em_ [nm] (film)^b^	Δ 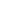 [cm^−1^]^c^ (film)

1	**8a**	510 (23600) 330 (20100) 290 (33200)	519	655	4000
2	**8b**	513 (24200) 327 (25800) 267 (56300)	523	662	4000
3	**8c**	513 (17900) 259 (40200)	525	665	4000
4	**8d**	517 (21500) 298 (39700) 265 (51300)	527	665	3900
5	**8e**	522 (14900) 317 (29300)	532	665	3800
6	**10a**	587 (33600) 290 (20400)	601	–	–
7	**10b**	592 (57500) 276 (51400)	623	–	–
8	**10c**	577 (30200) 257 (66400)	599	–	–
9	**10d**	592 (34000) 375 (15200) 269 (36100)	604	–	–
10	**10e**	597 (50900) 373 (40300) 266 (37300)	609	–	–
11	**10f**	591 (28700)	604	–	–
12	**10g**	597 (39400) 376 (20400) 276 (40700)	607	–	–
13	**10h**	563 (68900) 345 (24500)	617 572 (sh)	–	–

^a^Recorded in dichloromethane. ^b^Prepared by dropcasting. ^c^Δ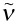 = λ_max,abs_^−1^ − λ_max,em_^−1^ [cm^−1^].

**Figure 4:**
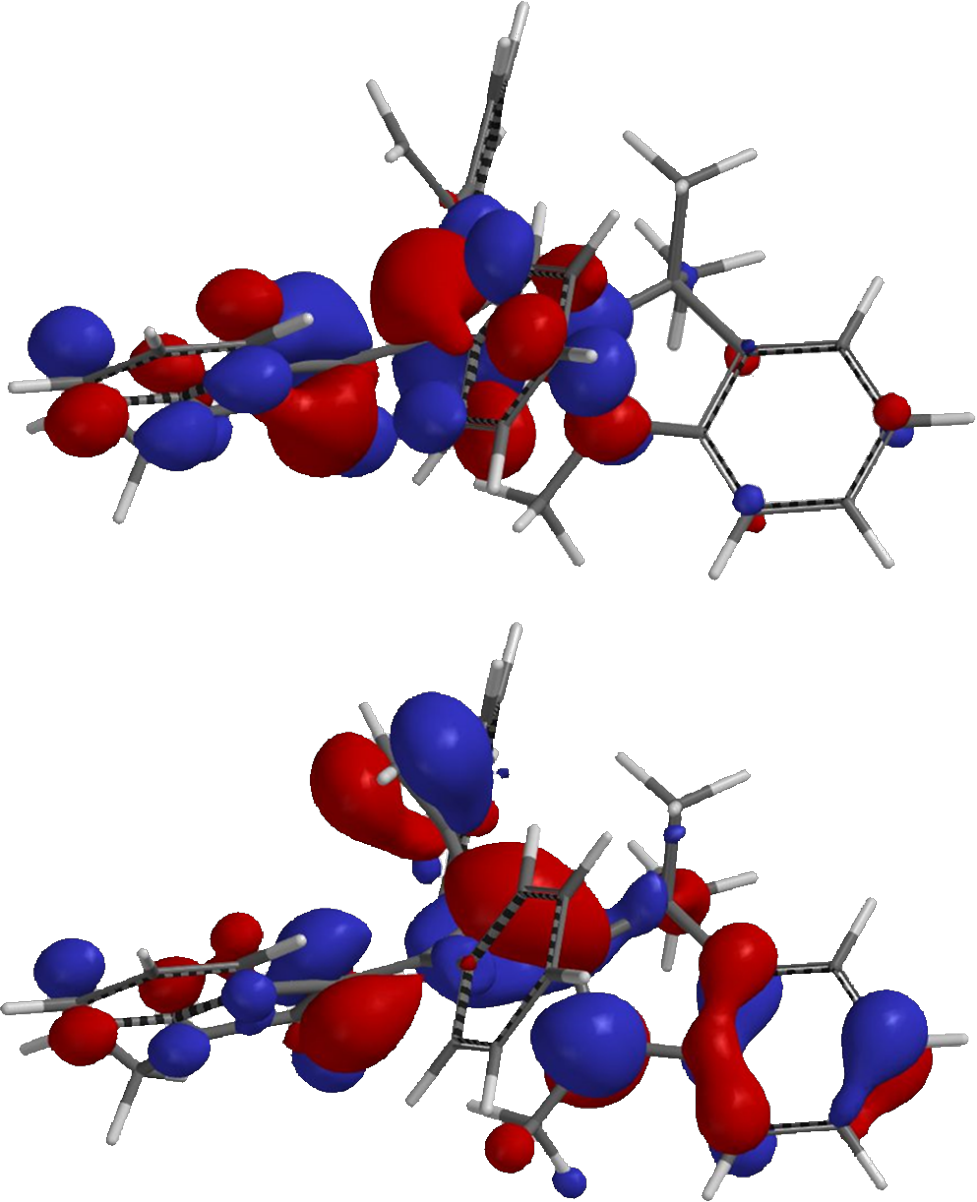
DFT-computed (B3LYP functional, 6-31G* basis set) HOMO (left) and LUMO (right) of merocyanine **8a**.

The 1-styryleth-2-enylideneindolones **8** are deep red in solution and in the films with intense, broad unstructured absorption bands between 510 and 522 nm in dichloromethane solutions, whereas the maxima of the films are slightly red-shifted and appear between 519 and 532 nm as a result of a *J*-aggregation [45]. Apparently, the aryl substitution on the triene moiety only affects the absorption bands to a minor extent. The merocyanine character of the 1-styryleth-2-enylideneindolones **8** is additionally supported by quantum chemical calculations on the DFT level (B3LYP functional, 6-31G* basis set) [40]. The computed Kohn–Sham frontier molecular orbitals of compound **8a** clearly indicate a charge transfer from the aminovinyl dominated donor fragment in the HOMO to the indolone-centered LUMO, which is generally responsible for the intense longest-wavelength absorption band upon optical excitation (Figure 4).

**Figure 5:**
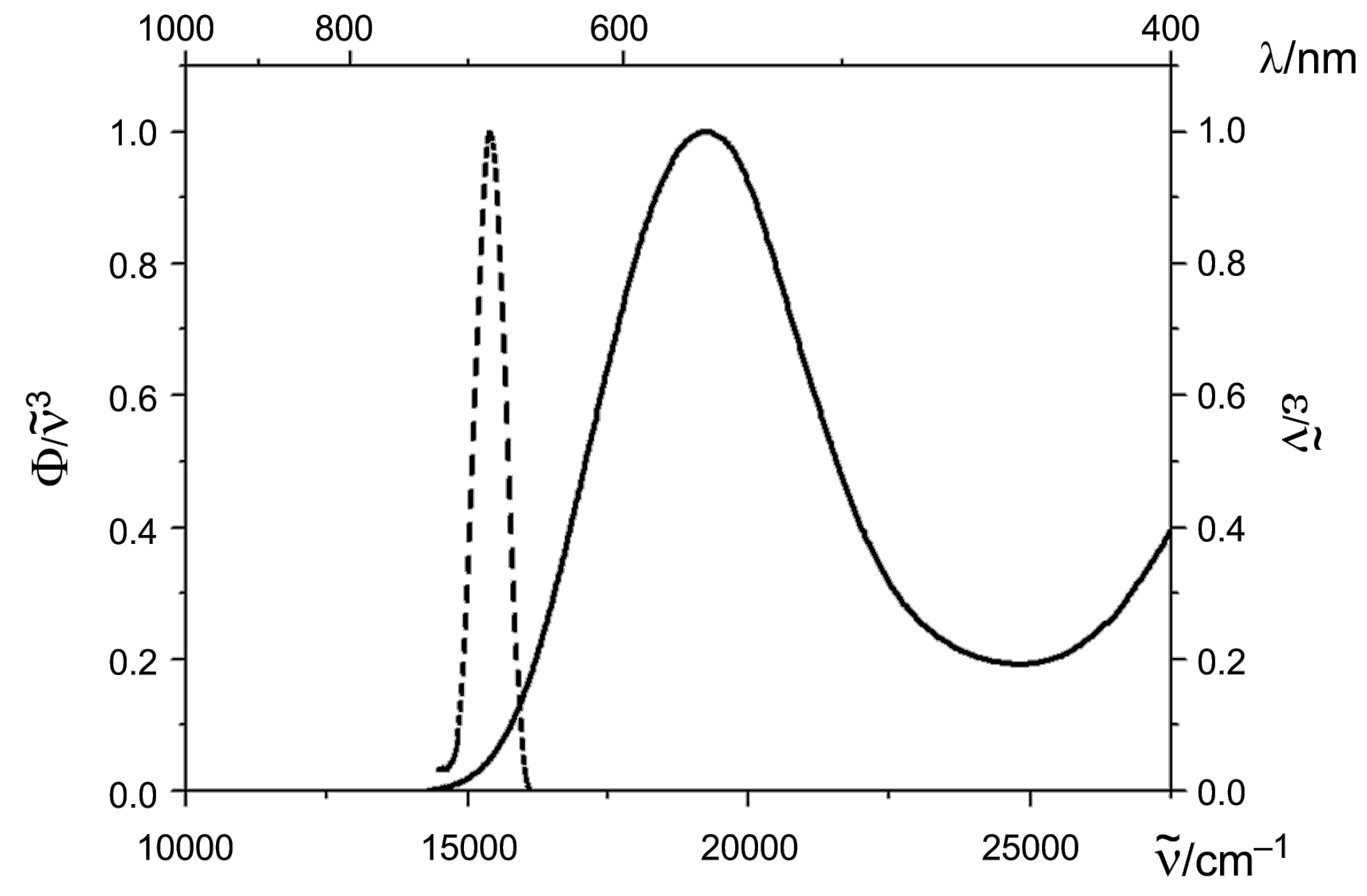
Absorption and emission spectrum of the dropcasted film of compound **8a** (recorded at room temperature, normalized spectra, λ_max,exc_ = 490 nm).

In contrast to the deep-red luminescence of both amorphous films and dyes in the solid state with sharp bands between 644 and 665 nm (Figure 5), the emission in solution is completely quenched. As already witnessed for the film absorption maxima of the 4-amino-prop-3-enylideneindolones **4** [30,32] the *J*-aggregation results in an induced emission [46–47] by Davydov splitting of the vibrationally relaxed lowest excited-state energy level [48–49] and causes a significant red shift of the sharp emission bands.

**Figure 6:**
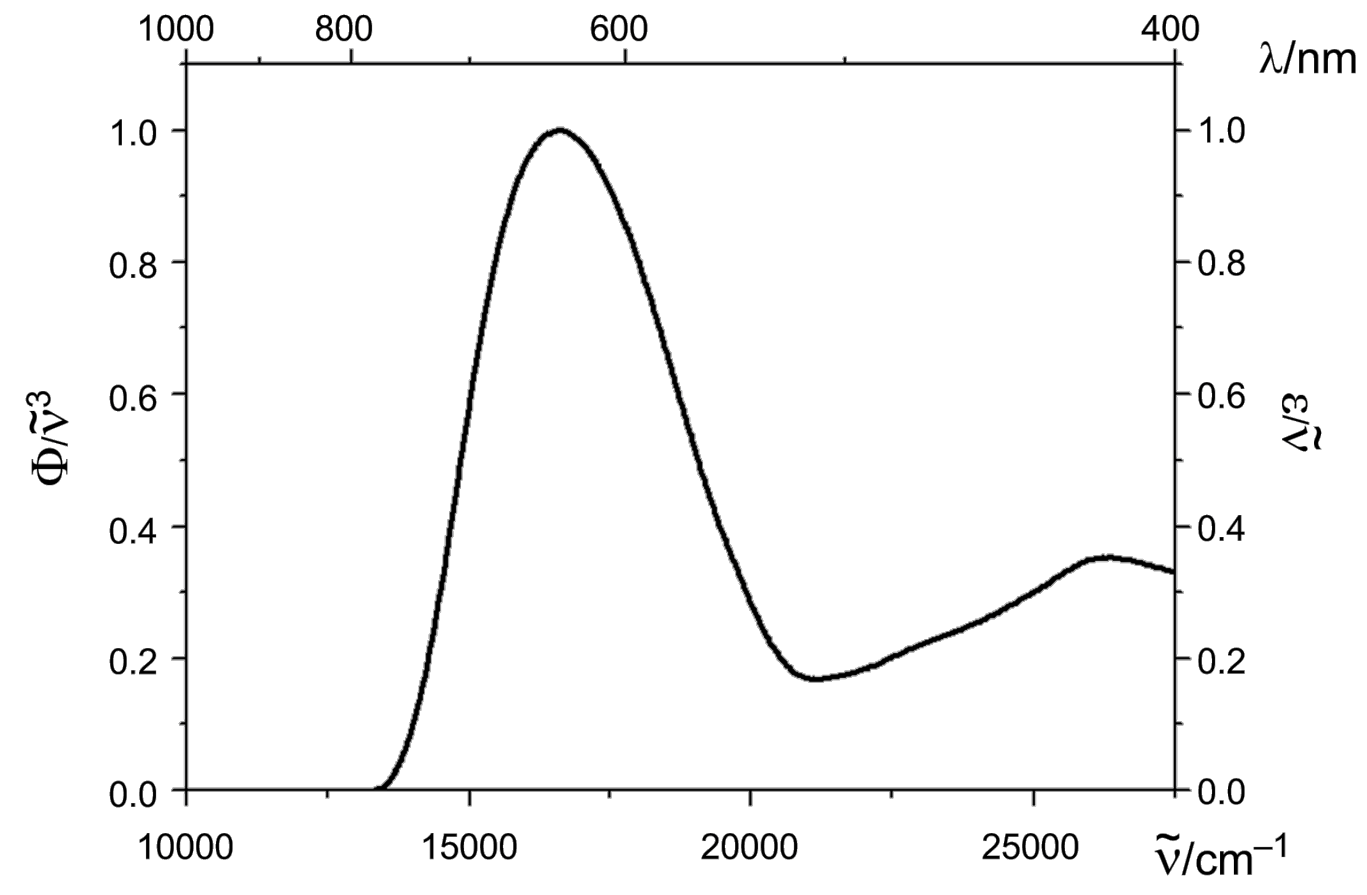
Absorption spectrum of the dropcasted film of compound **10d** (recorded at room temperature, normalized spectrum).

**Figure 7:**
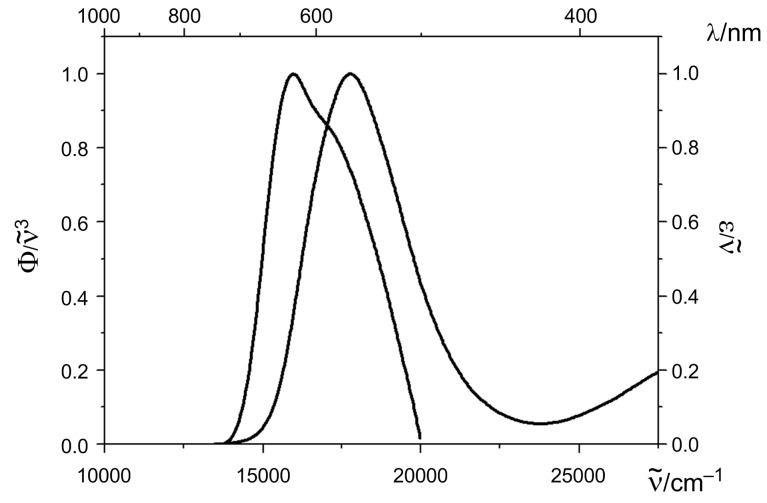
Absorption spectra of compound **10h** in dichloromethane (right trace) and of the dropcasted film (left trace) (recorded at room temperature, normalized spectrum).

The 4-(1,3,3-trimethylindolin-2-ylidene)but-2-en-1-ylideneindolones **10a–g** are dark-blue to black solids and display in dichloromethane solutions broad unstructured longest wavelength absorption bands in a range from 577 to 597 nm with high molar extinction coefficients. As a consequence of *J*-aggregation the absorption bands of the films are red-shifted and appear between 599 and 623 nm (Table 4, Figure 6). The benzothiazol-terminated merocyanine **10h** displays in dichloromethane solution a hypsochromically shifted absorption band at 563 nm with the highest molar extinction coefficient (Table 4, entry 13), yet for the amorphous film the most pronounced bathochromic shift in the series of the merocyanines **10** (Figure 7). In the solid state a merocyanine characteristic metallic green luster can be seen. Again, the aryl substitution on the triene moiety only affects the absorption bands to a minor extent.

For elucidation of the absorption characteristics of the 4-(1,3,3-trimethylindolin-2-ylidene)but-2-en-1-ylideneindolones **10**, a thorough geometry optimization of the ground-state structure of compound **10a** was performed using Gaussian09 [50] with the B3LYP functional [51–55] and the Pople 6-311G(d,p) basis set [56]. For a better comparison with the experimentally determined solution spectrum the calculation was carried out using the Polarizable Continuum Model (PCM) applying dichloromethane as a solvent [57]. The minimum structure of **10a** was unambiguously confirmed by an analytical frequency analysis.

The optimized structure of **10a** was submitted to a TD-DFT calculation for assigning the experimentally determined absorption characteristics (Table 5). Therefore, the hybrid exchange–correlation functional CAM-B3LYP [58] was implemented and a non-equilibrium solvation [59–63] for the state-specific solvation of the vertical excitation was included.

**Table 5:** Experimental and TD-DFT computed (CAM-B3LYP 6-311G(d,p)) absorption maxima of the 4-(1,3,3-trimethylindolin-2-ylidene)but-2-en-1-ylideneindolone 2*E*,4*Z*,6*Z*-**10a**.

Structure	Experimental λ_max,abs_ [nm]^a^	Computed λ_max,abs_ [nm]	Dominant contributions

2*E*,4*Z*,6*Z*-**10a**	290	286	HOMO → LUMO+2 (56%)
	–	345	HOMO−1 → LUMO (89%)
	587	541	HOMO → LUMO (96%)

^a^Recorded in dichloromethane.

**Figure 8:**
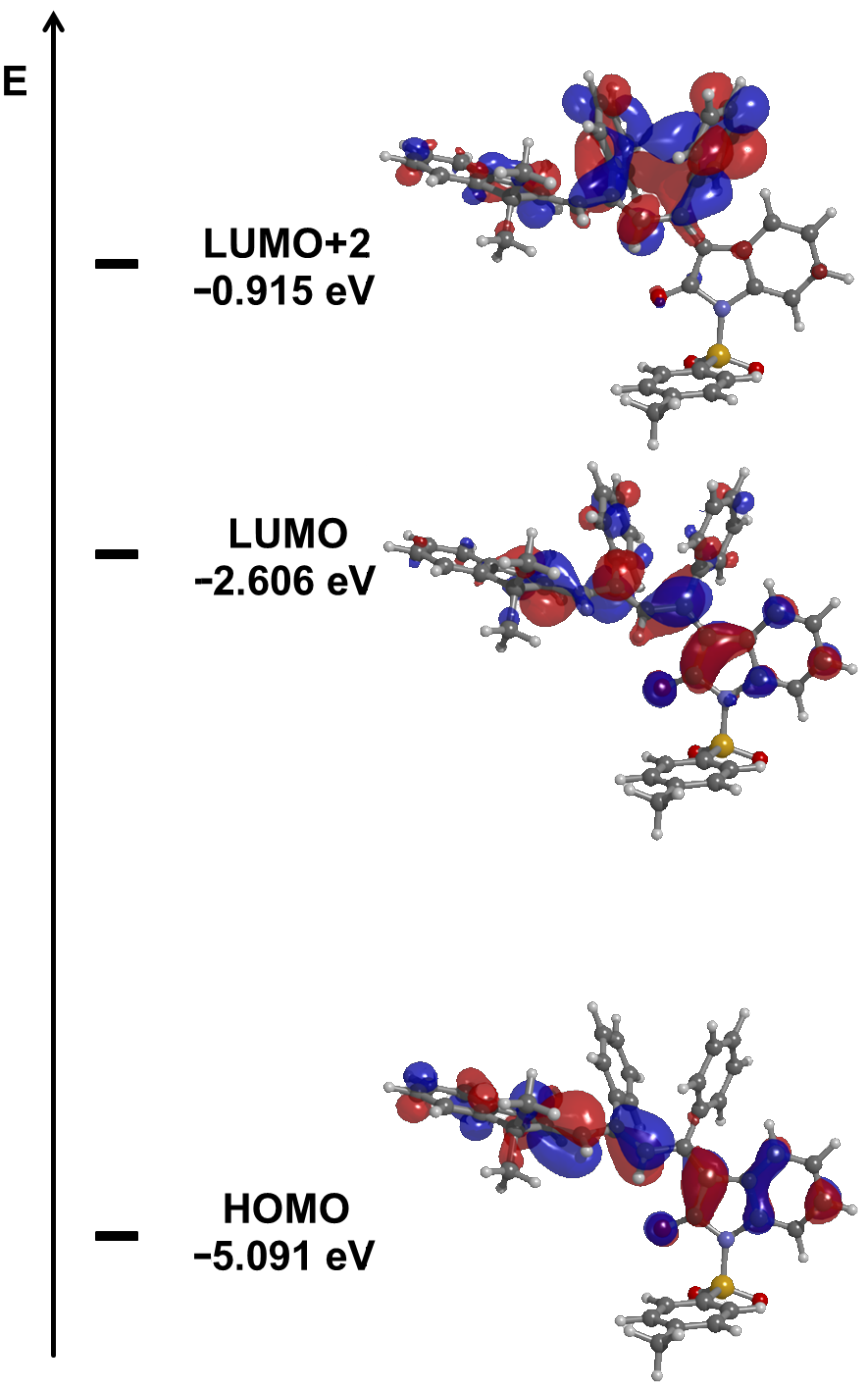
DFT-computed (B3LYP functional, 6-311G(d,p) basis set) FMOs (HOMO, bottom; LUMO (center), and LUMO+2 (top)) of merocyanine 2*E*,4*Z*,6*Z*-**10a**.

The computations reveal that the longest wavelength absorption maximum appears at 541 nm, i.e., at a comparable energy as in the experimental spectrum. This transition is exclusively dominated by the HOMO–LUMO transition. The computed Kohn–Sham frontier molecular orbitals of structure **10a** clearly indicate the charge-transfer character from HOMO to LUMO along the triene axis in the molecule, which generates the intense longest wavelength absorption band upon optical excitation (Figure 8). While the phenyl substituents on the triene chromophore largely contribute to shorter wavelength absorption bands, the tosyl moiety does not display any coefficient density in the FMOs and, therefore, qualifies as a favorable electronic innocent bridge for ligating other chromophores to this novel class of merocyanines.

## Conclusion

In conclusion, we enabled the diversity-oriented synthesis of novel triene merocyanines with intense bathochromic absorption by a consecutive three-component insertion–coupling–addition sequence in good to excellent yields. While the *N*-substituent on the indolone moiety exerts minute electronic differences in the dipolar intermediate, which is responsible for the bifurcation as supported by computational studies, enamine nucleophiles favorably lead to 1-styryleth-2-enylideneindolones diastereomers for *N*-methyl-substituted anilides. The *S*,*N*-ketene acetal derived from dimethyl benzothiazolium favors the formation of the corresponding 4-(3-methylbenzo[*d*]thiazol-2(3*H*)-ylidene)but-2-en-1-ylideneindolone. 4-(1,3,3-Trimethylindolin-2-ylidene)but-2-en-1-ylideneindolones are also the exclusive products for *N*-tosylanilides as starting materials. As a result of aggregation-induced luminescence, 1-styryleth-2-enylideneindolones display in films and in the solid state distinct and intensive deep-red emission upon excitation of the longest wavelength absorption band. 4-(1,3,3-Trimethylindolin-2-ylidene)but-2-en-1-ylideneindolones neither luminesce in solution nor in the solid state upon electronic excitation, yet, they display broad absorption bands and computations suggest, that novel types of panchromatic absorbing bichromophores should be readily available by ligating the second chromophore via the electronically nonperturbing *N*-sulfonyl moiety. Synthetic, photophysical, and computational studies addressing aggregating broad-band absorbing bichromophores are currently underway.

## Experimental

**8c**: In a flame-dried and argon-flushed Schlenk tube the iodophenylanilide **5a** (361 mg, 1.00 mmol), alkyne **6c** (150 mg, 1.10 mmol), and dry, degassed THF (5 mL) were placed. After the addition of PdCl_2_(PPh_3_)_2_ (35 mg, 0.05 mmol), and CuI (10 mg, 0.05 mmol), diisopropylethylamine (1.7 mL, 10 mmol) was added and the reaction mixture was stirred at rt for 16 h. Then, Fischer’s base (**7**, 346 mg, 2.00 mmol), and EtOH (2 mL) were added. The sealed reaction vessel was placed in a thermostatted oil bath at 80 °C and stirred for 48 h. After cooling to rt the solvents were removed in vacuo and the residue was chromatographed on silica gel (hexane/EtOAc 9:1) to give the 1-styryleth-2-enylideneindolone **8c** (441 mg, 0.82 mmol, 82%) as violet solid, dr = 56:44. Mp 228 °C; ^1^H NMR (300 MHz, CDCl_3_) δ 1.46–1.58 (m, 6H), 2.75–3.29 (m, 6H), 4.92 (0.56H), 5.61 (s, 0.56H), 5.78 (s, 0.56H), 6.25 (d, *J* = 7.8 Hz, 0.56H), 6.40–6.56 (m, 2H), 6.60–6.67 (m, 1H), 6.80–7.29 (m, 11H), 7.32 (d, *J* = 7.4 Hz, 0.56H); additional signals for the minor diastereomer: δ 4.61 (s, 0.44H), 5.33 (s, 0.44H), 6.73 (d, *J* = 7.5 Hz, 0.88H), 7.49 (d, *J* = 7.1 Hz, 0.44H); ^13^C NMR (75 MHz, CDCl_3_) δ 25.2 (CH_3_), 26.2 (CH_3_), 36.2 (CH_3_), 49.4 (C_quat_), 106.8 (C_quat_), 107.1 (CH), 107.9 (CH), 115.0 (CH_2_), 120.4 (CH), 121.3 (CH), 122.2 (CH), 124.3 (C_quat_), 124.8 (C_quat_), 126.4 (CH), 127.4 (CH), 127.8 (CH), 128.0 (CH), 128.9 (CH), 129.5 (CH), 130.0 (CH), 131.2 (CH), 132.9 (C_quat_), 133.2 (C_quat_), 140.2 (C_quat_), 140.4 (C_quat_), 140.5 (C_quat_), 141.4 (C_quat_), 144.4 (C_quat_), 145.5 (C_quat_), 153.9 (C_quat_), 165.4 (C_quat_); additional signals for the minor diastereomer: δ 26.3 (CH_3_), 37.4 (CH_3_), 50.1 (C_quat_), 107.2 (C_quat_), 107.2 (CH), 120.1 (CH_2_), 120.8 (CH), 121.6 (CH), 122.5 (CH), 126.5 (CH), 127.5 (CH), 128.2 (CH), 128.3 (CH), 128.4 (CH), 128.6 (CH), 129.0 (CH), 129.5 (CH), 132.5 (CH), 154.4 (C_quat_), 167.5 (C_quat_); EIMS (70 eV) *m*/*z* (% relative intensity): 544 ([^37^Cl–M]^+^, 23), 542 ([^35^Cl–M]^+^, 100), 382 ([C_25_H_18_^35^ClNO]^+^, 22), 158 ([C_11_H_12_N]^+^, 38); IR (KBr) 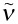: 3080, 3053, 2968, 2924, 2862, 1654, 1598, 1541, 1471, 1456, 1438, 1413, 1373, 1352, 1336, 1288, 1263, 1236, 1203, 1138, 1122, 1085, 1074, 1024, 1009, 958, 925, 893, 846, 825, 799, 732, 711, 693, 650, 619 cm^−1^; UV–vis (CH_2_Cl_2_) λ_max_, nm (ε): 259 (40200), 513 (17900); HRMS (*m*/*z*) calcd for C_36_H_31_^35^ClN_2_O: 542.2125; found: 542.2119; Anal. calcd for C_36_H_31_ClN_2_O (543.1): C, 79.61; H, 5.75; N, 5.16; found: C, 79.80; H, 6.02; N, 5.16.

**10a**: In a flame-dried and argon-flushed Schlenk tube iodophenylanilide **5b** (501 mg, 1.00 mmol), alkyne **6a** (112 mg, 1.10 mmol), and dry, degassed THF (5 mL) were placed. After the addition of PdCl_2_(PPh_3_)_2_ (35 mg, 0.05 mmol), and CuI (10 mg, 0.05 mmol), diisopropylethylamine (1.7 mL, 10 mmol) was added and the reaction mixture was stirred at rt for 16 h. Then, the enamine **7** (346 mg, 2.00 mmol) and EtOH (2 mL) were added. The sealed reaction vessel was placed in a thermostatted oil bath at 80 °C and stirred for 48 h. After cooling to rt the solvents were removed in vacuo and the residue was chromatographed on silica gel (hexane/EtOAc 4:1) to give the 4-(1,3,3-trimethylindolin-2-ylidene)but-2-en-1-ylideneindolone **10a** (636 mg, 98%) as bluish-black solid. Mp 141 °C; ^1^H NMR (300 MHz, CDCl_3_) δ 0.85 (s, 6H), 2.30 (s, 3H), 2.31 (s, 3H), 4.68 (d, *J* = 1.4 Hz, 1H), 5.70 (d, *J* = 7.3 Hz, 1H), 6.27 (d, *J* = 7.8 Hz, 1H), 6.56 (t, *J* = 7.8 Hz, 1H), 6.74 (dt, *J* = 7.3, 0.7 Hz, 1H), 6.90–7.22 (m, 12H), 7.32 (dd, *J* = 7.7, 1.8 Hz, 2H), 7.66 (d, *J* = 1.4 Hz, 1H), 7.80 (dd, *J* = 8.2, 2.2 Hz, 1H), 7.86 (d, *J* = 7.8 Hz, 1H), 7.92 (d, *J* = 8.4 Hz, 2H); ^13^C NMR (75 MHz, CDCl_3_) δ 21.9 (CH_3_), 29.4 (CH_3_), 34.4 (CH_3_), 46.7 (C_quat_), 94.3 (CH), 107.4 (CH), 113.0 (CH), 118.2 (C_quat_), 120.8 (CH), 121.8 (CH), 122.7 (CH), 123.4 (CH), 124.6 (CH), 125.4 (C_quat_), 127.3 (CH), 127.8 (CH), 127.9 (CH), 128.1 (CH), 128.7 (CH), 128.9 (CH), 129.1 (CH), 129.3 (CH), 129.5 (CH), 129.8 (CH), 136.4 (C_quat_), 137.1 (C_quat_), 138.2 (C_quat_), 140.7 (C_quat_), 144.4 (C_quat_), 145.1 (C_quat_), 146.4 (C_quat_), 153.9 (C_quat_), 155.9 (C_quat_), 161.8 (C_quat_), 165.9 (C_quat_). EIMS (70 eV) *m/z* (% relative intensity): 648 ([M]^+^, 4), 493 ([C_35_H_29_N_2_O]^+^, 6), 334 (31), 321 (11), 306 (11), 291 (12), 222 (11), 218 (37), 144 (33), 142 (47), 132 (27), 127 (22), 117 (16), 105 (53), 91 (100); IR (KBr) 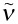: 3051, 2964, 2924, 2862, 1691, 1577, 1504, 1485, 1465, 1454, 1442, 1400, 1367, 1334, 1315, 1290, 1246, 1217, 1176, 1161, 1130, 1161, 1130, 1116, 1085, 1076, 1060, 1018, 1001, 960, 943, 925, 873, 854, 815, 773, 742, 725, 686, 663, 651, 611 cm^−1^; UV–vis (CH_2_Cl_2_) λ_max_, nm (ε): 290 nm (20400), 587 (33600); Anal. calcd for C_42_H_36_N_2_O_3_S (648.8): C, 77.75; H, 5.59; N, 4.32; found: C, 77.58; H, 5.41; N, 4.29.

## Supporting Information

File 1 Experimental procedures, spectroscopic and analytical data, and copies of NMR spectra of compounds **8** and **10**.

## References

[1] Müller T J J, Bunz U H F (2007). Functional Organic Materials. Syntheses, Strategies, and Applications.

[2] Müllen K, Wegner G (1998). Electronic Materials: The Oligomer Approach.

[3] Müllen K, Scherf U (2006). Organic Light-Emitting Diodes – Synthesis, Properties, and Applications.

[4] Kim E, Park S B (2009). Chem–Asian J.

[5] Cairo C W, Key J A, Sadek C M (2010). Curr Opin Chem Biol.

[6] Wagenknecht H-A (2008). Ann N Y Acad Sci.

[7] Hamer F M (1964). The Cyanine Dyes and Related Compounds.

[8] Mishra A, Behera R K, Behera P K, Mishra B K, Behera G B (2000). Chem Rev.

[9] Kulinich A V, Ishchenko A A (2009). Russ Chem Rev.

[10] Peng X-H, Zhou X-F, Carroll S, Geise H J, Peng B, Dommisse R, Esmans E, Carleer R (1996). J Mater Chem.

[11] Shirinian V Z, Shimkin A A (2008). Top Heterocycl Chem.

[12] Kovtun Yu P, Prostota Ya O, Shandura M P, Poronik Ye M, Tolmachev A I (2004). Dyes Pigm.

[13] Kovtun Y P, Prostota Y O, Tolmachev A I (2003). Dyes Pigm.

[14] Yagi S, Maeda K, Nakazumi H (1999). J Mater Chem.

[15] Kay A J, Woolhouse A D, Gainsford G J, Haskell T G, Barnes T H, McKinnie I T, Wyss C P (2001). J Mater Chem.

[16] Würthner F (1999). Synthesis.

[17] Würthner F, Yao S (2003). J Org Chem.

[18] Yao S, Beginn U, Gress T, Lysetska M, Würthner F (2004). J Am Chem Soc.

[19] Müller T J J, Müller T J J, Bunz U H F (2007). Diversity-oriented Synthesis of Chromophores by Combinatorial Strategies and Multi-component Reactions. Functional Organic Materials. Syntheses, Strategies, and Applications.

[20] Müller T J J, D'Souza D M (2008). Pure Appl Chem.

[21] Yi C, Blum C, Liu S-X, Frei G, Neels A, Stoeckli-Evans H, Leutwyler S, Decurtins S (2008). Tetrahedron.

[22] Samanta A, Vendrell M, Das R, Chang Y-T (2010). Chem Commun.

[23] Vendrell M, Lee J-S, Chang Y-T (2010). Curr Opin Chem Biol.

[24] Main M, Snaith J S, Meloni M M, Jauregui M, Sykes D, Faulkner S, Kenwright A M (2008). Chem Commun.

[25] Briehn C A, Bäuerle P (2002). Chem Commun.

[26] Briehn C A, Schiedel M-S, Bonsen E M, Schuhmann W, Bäuerle P (2001). Angew Chem, Int Ed.

[27] Willy B, Müller T J J (2009). Curr Org Chem.

[28] Willy B, Müller T J J (2008). ARKIVOC.

[29] Müller T J J (2012). Synthesis.

[30] Muschelknautz C, Mayer B, Rominger F, Müller T J J (2013). Chem Heterocycl Compd.

[31] Muschelknautz C, Frank W, Müller T J J (2011). Org Lett.

[32] D'Souza D M, Muschelknautz C, Rominger F, Müller T J J (2010). Org Lett.

[33] Willy B, Müller T J J (2011). Org Lett.

[34] Willy B, Müller T J J (2008). Eur J Org Chem.

[35] Willy B, Dallos T, Rominger F, Schönhaber J, Müller T J J (2008). Eur J Org Chem.

[36] Braun R U, Zeitler K, Müller T J J (2001). Org Lett.

[37] Braun R U, Müller T J J (2004). Synthesis.

[38] Schönhaber J, Müller T J J (2011). Org Biomol Chem.

[39] D'Souza D M, Kiel A, Herten D P, Rominger F, Müller T J J (2008). Chem–Eur J.

[40] D'Souza D M, Rominger F, Müller T J J (2005). Angew Chem, Int Ed.

[41] Schönhaber J, Frank W, Müller T J J (2010). Org Lett.

[42] Karpov A S, Müller T J J (2003). Org Lett.

[43] Karpov A S, Müller T J J (2003). Synthesis.

[44] (2008). Spartan ’08.

[45] Möbius D (1995). Adv Mater.

[46] Hong Y, Lam J W Y, Tang B Z (2011). Chem Soc Rev.

[47] Hong Y, Lam J W Y, Tang B Z (2009). Chem Commun.

[48] Davydov A S (1971). Theory of Molecular Excitons.

[49] Pope M, Swenberg C E (1982). Electronic Processes in Organic Crystals.

[50] (2009). Gaussian 09.

[51] Lee C, Yang W, Parr R G (1988). Phys Rev B.

[52] Becke A D (1993). J Chem Phys.

[53] Becke A D (1993). J Chem Phys.

[54] Kim K, Jordan K D (1994). J Phys Chem.

[55] Stephens P J, Devlin F J, Chabalowski C F, Frisch M J (1994). J Phys Chem.

[56] Krishnan R, Binkley J S, Seeger R, Pople J A (1980). J Chem Phys.

[57] Scalmani G, Frisch M J (2010). J Chem Phys.

[58] Yanai T, Tew D P, Handy N C (2004). Chem Phys Lett.

[59] Berezhkovskii A M (1992). Chem Phys.

[60] Cammi R, Tomasi J (1995). Int J Quantum Chem, Quantum Chem Symp.

[61] Mennucci B, Cammi R, Tomasi J (1998). J Chem Phys.

[62] Li X-Y, Fu K-X (2004). J Comput Chem.

[63] Cammi R, Corni S, Mennucci B, Tomasi J (2005). J Chem Phys.

