# Supplementary material for: One-pot three-component synthesis and photophysical characteristics of novel triene merocyanines
Source: Beilstein J Org Chem. 2014 Mar 5;10:599–612. doi: 10.3762/bjoc.10.51 (PMC3999876; doi:10.3762/bjoc.10.51)
Supplement: File 1 — Experimental procedures, spectroscopic and analytical data, and copies of NMR spectra of compounds 8 and 10. [file Beilstein_J_Org_Chem-10-599-s001.pdf]

One-pot three-component synthesis and photophysical characteristics of novel triene merocyanines

Christian Muschelknautz, Robin Visse, Jan Nordmann, and Thomas J. J. Müller*

Address: Institut für Organische Chemie und Makromolekulare Chemie, Heinrich-Heine-Universität Düsseldorf, Universitätsstr. 1, D-40225 Düsseldorf, Germany.

Email: Thomas J. J. Müller - ThomasJJ.Mueller@uni-duesseldorf.de

*Corresponding author

Dedicated to Dr. Hans-Ulrich Wagner on the occasion of his 75th birthday.

Experimental procedures, spectroscopic and analytical data, and copies of NMR spectra of compounds 8 and 10.

Content

1. General considerations S4

2. General Procedure for the synthesis of 1-styryleth-2-enylideneindolones **8** S5

2.1. 3-(1,3-Diphenyl-2-(1,3,3-trimethylindolin-2-ylidene)but-3-en-1-ylidene)-1-methylindolin-2-one (**8a**) S5

2.2. 3-(3-(4-Methoxyphenyl)-1-phenyl-2-(1,3,3-trimethylindolin-2-ylidene)but-3-en-1-ylidene)-1-methylindolin-2-one (**8b**) S6

2.3. 3-(3-(4-Chlorophenyl)-1-phenyl-2-(1,3,3-trimethylindolin-2-ylidene)but-3-en-1-ylidene)-1-methylindolin-2-one (**8c**) S7

2.4. 4-(4-(1-Methyl-2-oxoindolin-3-ylidene)-4-phenyl-3-(1,3,3-trimethylindolin-2-ylidene)but-1-en-2-yl)benzonitrile (**8d**) S8

2.5. 1-Methyl-3-(3-(4-nitrophenyl)-1-phenyl-2-(1,3,3-trimethylindolin-2-ylidene)but-3-en-1-ylidene)indolin-2-one (**8e**) S8

3. General Procedure for the synthesis of 4-(1,3,3-trimethylindolin-2-ylidene)but-2-en-1-ylideneindolones **10** S10

3.1. (*Z*)-3-((2*Z*,4*E*)-1,3-Diphenyl-4-(1,3,3-trimethylindolin-2-ylidene)but-2-en-1-ylidene)-1-tosylindolin-2-one (**10a**) S11

3.2. (*Z*)-3-((2*Z*,4*E*)-3-(4-Aminophenyl)-1-phenyl-4-(1,3,3-trimethylindolin-2-ylidene)but-2-en-1-ylidene)-1-tosylindolin-2-one (**10b**) S12

3.3. (*Z*)-3-((2Z,4*E*)-3-(4-Chlorphenyl)-1-phenyl-4-(1,3,3-trimethylindolin-2-yliden)but-2-en-1-yliden)-1-tosylindolin-2-one (**10c**) S13

3.4. 4-((1*Z*,2*Z*,4*E*)-4-(2-Oxo-1-tosylindolin-3-ylidene)-4-phenyl-1-(1,3,3-trimethyl-indolin-2-ylidene)but-2-en-2-yl)benzonitrile (**10d**) S14

3.5. (*Z*)-3-((2*Z*,4*E*)-3-(4-(*tert*-Butyl)phenyl)-1-(4-chlorophenyl)-4-(1,3,3-trimethyl-indolin-2-ylidene)but-2-en-1-ylidene)-1-tosylindolin-2-one (**10e**) S14

3.6. (*Z*)-3-((2*Z*,4*E*)-1,3-Bis(4-chlorophenyl)-4-(1,3,3-trimethylindolin-2-ylidene)but-2-en-1-ylidene)-1-tosylindolin-2-one (**10f**) S15

3.7. 4-((1*Z*,2*Z*,4*E*)-4-(4-Chlorophenyl)-4-(2-oxo-1-tosylindolin-3-ylidene)-1-(1,3,3-tri-methylindolin-2-ylidene)but-2-en-2-yl)benzonitrile (**10g**) S16

3.8. (*Z*)-3-((2*Z*,4*Z*)-3-(4-Chlorophenyl)-4-(3-methylbenzo[*d*]thiazol-2(3*H*)-ylidene)-1-phenylbut-2-en-1-ylidene)-1-methylindolin-2-one (**10h**) S17

4.1. 3-(1,3-Diphenyl-2-(1,3,3-trimethylindolin-2-ylidene)but-3-en-1-ylidene)-1-methylindolin-2-one (**8a**) S18

4.2. 3-(3-(4-Methoxyphenyl)-1-phenyl-2-(1,3,3-trimethylindolin-2-ylidene)but-3-en-1-ylidene)-1-methylindolin-2-one (**8b**) S20

4.3. 3-(3-(4-Chlorophenyl)-1-phenyl-2-(1,3,3-trimethylindolin-2-ylidene)but-3-en-1-ylidene)-1-methylindolin-2-one (**8c**) ... S22
4.4. 4-(4-(1-Methyl-2-oxoindolin-3-ylidene)-4-phenyl-3-(1,3,3-trimethylindolin-2-ylidene)but-1-en-2-yl)benzonitrile (**8d**) ... S24
5.1. (*Z*)-3-((2*Z*,4*E*)-1,3-Diphenyl-4-(1,3,3-trimethylindolin-2-ylidene)but-2-en-1-ylidene)-1-tosylindolin-2-one (**10a**) ... S28
5.2. (*Z*)-3-((2*Z*,4*E*)-3-(4-Aminophenyl)-1-phenyl-4-(1,3,3-trimethylindolin-2-ylidene)but-2-en-1-ylidene)-1-tosylindolin-2-one (**10b**) ... S30
5.3. (*Z*)-3-((2*Z*,4*E*)-3-(4-Chlorphenyl)-1-phenyl-4-(1,3,3-trimethylindolin-2-yliden)but-2-en-1-yliden)-1-tosylindolin-2-on (**10c**) ... S32
5.4. 4-((1*Z*,2*Z*,4*E*)-4-(2-Oxo-1-tosylindolin-3-ylidene)-4-phenyl-1-(1,3,3-trimethyl-indolin-2-ylidene)but-2-en-2-yl)benzonitrile (**10d**) ... S34
5.5. (*Z*)-3-((2*Z*,4*E*)-3-(4-(*tert*-Butyl)phenyl)-1-(4-chlorophenyl)-4-(1,3,3-trimethyl-indolin-2-ylidene)but-2-en-1-ylidene)-1-tosylindolin-2-one (**10e**) ... S36
5.6. (*Z*)-3-((2*Z*,4*E*)-1,3-Bis(4-chlorophenyl)-4-(1,3,3-trimethylindolin-2-ylidene)but-2-en-1-ylidene)-1-tosylindolin-2-one (**10f**) ... S38
5.7. 4-((1*Z*,2*Z*,4*E*)-4-(4-Chlorophenyl)-4-(2-oxo-1-tosylindolin-3-ylidene)-1-(1,3,3-tri-methylindolin-2-ylidene)but-2-en-2-yl)benzonitrile (**10g**) ... S40
5.8. (*Z*)-3-((2*Z*,4*Z*)-3-(4-Chlorophenyl)-4-(3-methylbenzo[*d*]thiazol-2(3*H*)-ylidene)-1-phenylbut-2-en-1-ylidene)-1-methylindolin-2-one (**10h**) ... S42
6. Computational data of structures 2*E*,4*Z*,6*E*-**10a**, 2*E*,4*Z*,6*Z*-**10a**, 2*E*,4*Z*,6*Z*-**10h**, 2*E*,4*Z*,6*E*-**10h** ... S44
6.1. Structure 2*Z*,4*Z*-**8a** ... S45
6.2. Structure 2*Z*,4*E*-**8a** ... S47
6.3. Structure 2*E*,4*Z*,6*E*-**10a** ... S49
6.4. Structure 2*E*,4*Z*,6*Z*-**10a** ... S51
6.5. Structure 2*E*,4*Z*,6*Z*-**10h** ... S53
6.6. Structure 2*E*,4*Z*,6*E*-**10h** ... S55
6.7. Structure of the *N*-methyl allenyl enolate **16a** ... S57
6.8. Structure of the *N*-tosyl allenyl enolate **16b** ... S59
6.9. HOMO and LUMO of 2*Z*,4*Z*-**8a** ... S61
6.10. TD-DFT calculation of 2*E*,4*Z*,6*Z*-**10a** ... S61

1. General considerations

All reactions involving palladium–copper catalysis were performed in degassed oxygen-free solvents under a nitrogen atmosphere using Schlenk and syringe techniques. $PdCl_2(PPh_3)_2$, CuI, Fischer's base (**7**) and 2,3-dimethylbenzothiazolium iodide (**9**) were purchased from Acros and Aldrich (reagent grade) and used without further purification. Diisopropylethylamine was dried and distilled according to standard procedures.[1] Detailed preparative procedures including full analytics and ^{1}H, ^{13}C and 135-DEPT NMR spectra of the *o*-iodophenylanilides **5** have been previously reported.[2] Column chromatography was performed using silica gel 60 mesh 230–400 (Macherey-Nagel, Düren). TLC analysis was performed using silica gel plates (60 F254 Merck, Darmstadt), eluent hexane/EtOAc.

^{1}H NMR spectra were recorded on Bruker DRX 500 (500 MHz) and Bruker DRX 300 spectrometers (300 MHz), ^{13}C NMR spectra on Bruker DRX 500, Bruker DRX 300, and Bruker AC 300 spectrometers. The solvent used for all NMR spectra was $CDCl_3$ with Me_4Si as a internal standard. The assignments of quaternary carbon nuclei, CH, CH_2 and CH_3 groups were made by using 135-DEPT spectra. Mass spectra were recorded on Jeol JMS-700 and Finnigan TSQ 700 spectrometers. IR spectra were recorded on a Bruker Vector 22 FT-IR spectrometer as KBr discs. UV–vis spectra were recorded on a Hewlett Packard HP8452 A Diode Array spectrophotometer in CH_2Cl_2 solution. Fluorescence spectra were recorded on a Perkin Elmer LS-55 spectrometer. Elemental analyses were carried out in the microanalytical laboratories of the Institute of Pharmacy, Heinrich-Heine-Universität Düsseldorf. Melting points were determined on a Büchi Melting Point B-540 apparatus and are uncorrected.

For the measurements of the optical properties compounds **8** and **10** were dissolved in CH_2Cl_2 for the preparation of films on glass by slow evaporation. For the UV–vis measurements a thin film was dropcasted onto one side of the inner surface of a common glass cuvette. For the fluorescence measurements a thin film was dropcasted onto an object slide. The slide was placed into the cavity in a perpendicular orientation and with a 45° angle with respect to the optical pathway of a fluorescence spectrometer. The excitation wavelength was chosen in accordance to the longest absorption wavelength maximum as determined from the film UV–vis spectra.

2. General Procedure for the synthesis of 1-styryleth-2-enylideneindolones 8

In a flame-dried and argon-flushed Schlenk tube the iodo phenylanilide **5a** (361 mg, 1.00 mmol), alkyne **6** (1.10 mmol), and dry, degassed THF (5 mL) were placed (for experimental details see Table 1). After the addition of $PdCl_2(PPh_3)_2$ (35 mg, 0.05 mmol), and CuI (10 mg, 0.05 mmol), diisopropylethylamine (1.7 mL, 10 mmol) was added and the reaction mixture was stirred at rt for 16 h. Then, Fischer's base (**7**, 346 mg, 2.00 mmol), and EtOH (2 mL) were added. The sealed reaction vessel was placed in a thermostatted oil bath at 80 °C and stirred for 48 h. After cooling to rt the solvents were removed in vacuo and the residue was chromatographed on silica gel (hexane/EtOAc) to give the 1-styryleth-2-enylideneindolones **8** as violet solids.

Table S1: Experimental details of the three-component synthesis of the 1-styryleth-2-enylideneindolones **8**.

Entry	Alkyne **6**	1-Styryleth-2-enylideneindolones **8** (isolated yield)
1	112 mg (1.10 mmol) of phenylacetylene (**6a**)	473 mg (93%) of **8a**
2	145 mg (1.10 mmol) of 4-methoxyphenylacetylene (**6b**)	510 mg (81%) of **8b**
3	150 mg (1.10 mmol) of 4-chlorophenylacetylene (**6c**)	441 mg (82%) of **8c**
4	140 mg (1.10 mmol) of 4-cyanophenylacetylene (**6d**)	464 mg (87%) of **8d**
5	162 mg (1.10 mmol) of 4-nitrophenylacetylene (**6e**)	504 mg (91%) of **8e**

2.1. 3-(1,3-Diphenyl-2-(1,3,3-trimethylindolin-2-ylidene)but-3-en-1-ylidene)-1-methylindolin-2-one (8a)

After chromatography on silica gel (hexane/ethylacetate 9:1) compound **8a** was obtained as a violet solid (473 mg, 0.93 mmol, 93%), dr = 56:44, Mp 181 °C. ^{1}H NMR (500 MHz, $CDCl_3$): δ 1.61-1.74 (m, 6 H), 3.19-3.47 (m, 6 H), 4.97 (s, 0.56 H), 5.71 (s, 0.56 H), 5.82 (d, J = 7.7 Hz, 0.56 H), 6.39-7.43 (m, 16 H), 7.58 (d, J = 6.3 Hz, 0.56 H); additional signals for the minor diastereomer: δ 4.68 (s, 0.44 H), 5.42 (s, 0.44 H), 6.26 (d, J = 7.2 Hz, 0.44 H), 7.66-7.72 (m, 0.44 H). ^{13}C NMR (125 MHz, $CDCl_3$): δ 25.4 (CH_3), 36.3 (CH_3), 37.4 (CH_3), 49.1 (C_{quat}), 106.9 (CH), 107.4 (CH), 114.7 (CH_2), 120.1 (C_{quat}), 120.4 (CH), 120.7 (CH), 121.6 (CH), 122.1 (CH), 123.9 (C_{quat}), 124.9 (C_{quat}), 126.9 (CH), 127.3 (CH), 127.5 (CH), 127.7 (CH), 128.1 (CH), 128.8

(CH), 131.2 (CH), 132.5 (CH), 140.6 (C_{quat}), 140.7 (C_{quat}), 141.1 (C_{quat}), 141.3 (C_{quat}), 141.6 (C_{quat}), 145.2 (C_{quat}), 145.3 (C_{quat}), 154.4 (C_{quat}), 165.3 (C_{quat}); additional signals for the minor diastereomer: δ 26.2 (CH_3), 49.4 (C_{quat}), 107.1 (CH), 107.9 (CH), 119.7 (CH_2), 121.1 (CH), 121.7 (CH), 122.4 (CH), 124.5 (C_{quat}), 125.6 (C_{quat}), 126.1 (CH), 127.0 (CH), 127.4 (CH), 127.6 (CH), 127.9 (CH), 128.2 (CH), 129.9 (CH), 131.5 (CH), 145.5 (C_{quat}), 154.8 (C_{quat}), 167.6 (C_{quat}). EI MS (70 eV, *m/z* (%)): 508 ($[M]^+$, 100), 493 ($[M – CH_3]^+$, 31), 349 ($[C_{25}H_{19}NO]^+$, 16), 158 (32). IR (KBr): $\tilde{\nu}$ = 2981, 2993, 2873, 1676, 1604, 1560, 1506, 1496, 1417, 1373, 1364, 1305, 1261, 1234, 1089, 1074, 1049, 1018, 1012, 1001, 918, 873, 743, 731, 698, 673, 648 cm^{-1}. UV/Vis (CH_2Cl_2): λ_{max} (ε) = 290 nm (33200), 330 (20100), 510 (23600). Anal. calcd. for $C_{36}H_{32}N_2O \cdot 0.02\ CHCl_3$ (508.7 + 2.4): C 83.29, H 6.22, N 5.38; Found: C 83.20, H 6.37, N 5.35.

2.2. 3-(3-(4-Methoxyphenyl)-1-phenyl-2-(1,3,3-trimethylindolin-2-ylidene)but-3-en-1-ylidene)-1-methylindolin-2-one (8b)

After chromatography on silica gel (hexane/ethylacetate 9:1) compound **8b** was obtained as a violet solid (510 mg, 0.81 mmol, 81%), dr = 62:38, Mp 226 °C. ^1H NMR (500 MHz, $CDCl_3$): δ 1.56-1-69 (m, 6 H), 3.14-3.28 (m, 6 H), 3.70-3.74 (m, 3 H), 4.86 (s, 0.62 H), 5.58 (s, 0.62 H), 5.81 (d, *J* = 7.7 Hz, 0.62 H), 6.25-7.69 (m, 16.38 H); additional signals for the minor diastereomer: δ 4.59 (s, 0.38 H), 5.32 (s, 0.38 H), 6.25 (m, 0.38 H). ^{13}C NMR (125 MHz, $CDCl_3$): δ 24.6 (CH_3), 26.0 (CH_3), 36.3 (CH_3), 49.1 (C_{quat}), 55.4 (CH_3), 106.2 (C_{quat}), 106.9 (CH), 113.2 (CH), 115.9 (CH_2), 120.0 (C_{quat}), 120.4 (CH), 121.6 (CH), 123.0 (CH), 124.5 (C_{quat}), 126.1 (CH), 127.4 (CH), 127.9 (CH), 128.2 (CH), 128.7 (CH), 129.2 (CH), 129.7 (CH), 130.0 (CH), 132.4 (C_{quat}), 134.6 (C_{quat}), 135.7 (C_{quat}), 137.2 (C_{quat}), 140.6 (C_{quat}), 141.1 (C_{quat}), 143.5 (C_{quat}), 145.6 (C_{quat}), 159.1 (C_{quat}), 165.4 (C_{quat}); signals for the minor diastereomer: δ 25.3 (CH_3), 26.2 (CH_3), 37.4 (CH_3), 55.6 (CH_3), 49.4 (C_{quat}), 107.2 (C_{quat}), 107.8 (CH), 114.6 (CH), 118.1 (CH_2), 121.1 (CH), 122.1 (CH), 122.5 (CH), 123.5 (C_{quat}), 123.6 (CH), 125.0 (C_{quat}), 127.6 (CH), 128.1 (CH), 128.6 (CH), 128.8 (CH), 129.4 (CH), 129.8 (CH), 131.1 (CH), 132.6 (C_{quat}), 134.5 (C_{quat}), 137.0 (C_{quat}), 137.4 (C_{quat}), 140.8 (C_{quat}), 141.3 (C_{quat}), 144.6 (C_{quat}), 154.7 (C_{quat}), 161.1 (C_{quat}), 167.3 (C_{quat}). EI MS: (70 eV, *m/z* (%)): 538 ($[M]^+$, 26), 463 ($[C_{31}H_{31}N_2O_2]^+$, 25), 365

($[C_{25}H_{19}NO_2]^+$, 100), 297 ($[C_{22}H_{17}O]^+$, 46). IR (KBr): $\tilde{\nu}$ = 3051, 3005, 2953, 2929, 2860, 1654, 1602, 1506, 1469, 1454, 1438, 1409, 1375, 1352, 1336, 1288, 1261, 1238, 1205, 1180, 1138, 1122, 1089, 1076, 1053, 1024, 958, 925, 898, 839, 815, 802, 746, 732, 725, 702, 663, 652 cm^{-1}. UV/Vis (CH_2Cl_2): λ_{max} (ε) = 267 nm (56300), 327 (25800), 513 (24200). HRMS (EI) calcd. for ($C_{37}H_{34}N_2O_2$): 538.2620; Found: 538.2616.

2.3. 3-(3-(4-Chlorophenyl)-1-phenyl-2-(1,3,3-trimethylindolin-2-ylidene)but-3-en-1-ylidene)-1-methylindolin-2-one (8c)

After chromatography on silica gel (hexane/ethylacetate 9:1) compound **8c** was obtained as a violet solid (441 mg, 0.82 mmol, 82%), dr = 56:44, Mp 228 °C. ^{1}H NMR (300 MHz, $CDCl_3$): δ 1.46-1.58 (m, 6 H), 2.75-3.29 (m, 6 H), 4.92 (0.56 H), 5.61 (s, 0.56 H), 5.78 (s, 0.56 H), 6.25 (d, J = 7.8 Hz, 0.56 H), 6.40-6.56 (m, 2 H), 6.60-6.67 (m, 1 H), 6.80-7.29 (m, 11 H), 7.32 (d, J = 7.4 Hz, 0.56 H); additional signals for the minor diastereomer: δ 4.61 (s, 0.44 H), 5.33 (s, 0.44 H), 6.73 (d, J = 7.5 Hz, 0.88 H), 7.49 (d, J = 7.1 Hz, 0.44 H). ^{13}C NMR (75 MHz, $CDCl_3$): δ 25.2 (CH_3), 26.2 (CH_3), 36.2 (CH_3), 49.4 (C_{quat}), 106.8 (C_{quat}), 107.1 (CH), 107.9 (CH), 115.0 (CH_2), 120.4 (CH), 121.3 (CH), 122.2 (CH), 124.3 (C_{quat}), 124.8 (C_{quat}), 126.4 (CH), 127.4 (CH), 127.8 (CH), 128.0 (CH), 128.9 (CH), 129.5 (CH), 130.0 (CH), 131.2 (CH), 132.9 (C_{quat}), 133.2 (C_{quat}), 140.2 (C_{quat}), 140.4 (C_{quat}), 140.5 (C_{quat}), 141.4 (C_{quat}), 144.4 (C_{quat}), 145.5 (C_{quat}), 153.9 (C_{quat}), 165.4 (C_{quat}); additional signals for the minor diastereomer: δ 26.3 (CH_3), 37.4 (CH_3), 50.1 (C_{quat}), 107.2 (C_{quat}), 107.2 (CH), 120.1 (CH_2), 120.8 (CH), 121.6 (CH), 122.5 (CH), 126.5 (CH), 127.5 (CH), 128.2 (CH), 128.3 (CH), 128.4 (CH), 128.6 (CH), 129.0 (CH), 129.5 (CH), 132.5 (CH), 154.4 (C_{quat}), 167.5 (C_{quat}). EI MS: (70 eV, m/z (%)): 544 ($[^{37}Cl\text{-}M]^+$, 23), 542 ($[^{35}Cl\text{-}M]^+$, 100), 382 ($[C_{25}H_{18}{}^{35}ClNO]^+$, 22), 158 ($[C_{11}H_{12}N]^+$, 38). IR (KBr): $\tilde{\nu}$ = 3080, 3053, 2968, 2924, 2862, 1654, 1598, 1541, 1471, 1456, 1438, 1413, 1373, 1352, 1336, 1288, 1263, 1236, 1203, 1138, 1122, 1085, 1074, 1024, 1009, 958, 925, 893, 846, 825, 799, 732, 711, 693, 650, 619 cm^{-1}. UV/Vis (CH_2Cl_2): λ_{max} (ε) = 259 nm (40200), 513 (17900). HRMS: calcd. for $C_{36}H_{31}{}^{35}ClN_2O$: 542.2125; Found: 542.2119. Anal. calcd. for $C_{36}H_{31}ClN_2O$ (543.1): C 79.61, H 5.75, N 5.16; Found: C 79.80, H 6.02, N 5.16.

2.4. 4-(4-(1-Methyl-2-oxoindolin-3-ylidene)-4-phenyl-3-(1,3,3-trimethylindolin-2-ylidene)but-1-en-2-yl)benzonitrile (8d)

After chromatography on silica gel (hexane/ethylacetate 4:1) compound **8d** was obtained as a violet solid (464 mg, 0.87 mmol, 87%), dr = 52:48, Mp 240 °C. ^{1}H NMR (500 MHz, $CDCl_3$): δ 1.52-1.63 (m, 6 H), 3.12-3.36 (m, 6 H), 5.14 (s, 0.52 H), 5.79 (s, 0.52 H), 5.86 (d, J = 7.7 Hz, 0.52 H), 6.43-7.49 (m, 15 H), 7.54 (d, J = 7.4 Hz, 0.52 H); additional signals for the minor diastereomer: δ 4.77 (s, 0.48 H), 5.47 (s, 0.48 H), 6.24 (d, J = 7.4 Hz, 0.48 H), 7.60 (d, J = 7.4 Hz, 0.48 H). ^{13}C NMR (125 MHz, $CDCl_3$): δ 25.1 (CH_3), 26.4 (CH_3), 36.2 (CH_3), 37.4 (CH_3), 49.5 (C_{quat}), 107.2 (CH), 107.4 (CH), 108.8 (C_{quat}), 116.7 (CH_2), 119.5 (C_{quat}), 120.6 (CH), 121.5 (CH), 121.8 (CH), 122.0 (CH), 123.6 (C_{quat}), 124.6 (C_{quat}), 125.4 (C_{quat}), 126.5 (CH), 128.1 (CH), 128.5 (CH), 129.3 (CH), 131.1 (CH), 131.8 (CH), 132.4 (CH), 140.2 (C_{quat}), 141.0 (C_{quat}), 144.4 (C_{quat}), 145.3 (C_{quat}), 146.1 (C_{quat}), 147.4 (C_{quat}), 148.8 (C_{quat}), 153.6 (C_{quat}), 165.4 (C_{quat}); additional signals for the minor diastereomer: δ 26.2 (CH_3), 29.4 (CH_3), 36.3 (CH_3), 50.3 (C_{quat}), 107.3 (CH), 108.0 (CH), 110.2 (C_{quat}), 120.5 (C_{quat}), 121.0 (CH), 121.7 (CH), 122.1 (CH), 122.4 (CH_2), 124.2 (C_{quat}), 126.7 (CH), 127.6 (CH), 127.9 (CH), 128.7 (CH), 130.2 (CH), 131.9 (CH), 140.3 (C_{quat}), 147.5 (C_{quat}). EI MS: (70 eV, *m/z* (%)): 533 ($[M]^+$, 100), 518 ($[M – CH_3]^+$, 24), 374 ($[C_{26}H_{18}N_2O]^+$, 18), 158 (30). IR (KBr): $\tilde{\nu}$ = 3053, 2968, 2924, 2862, 2225, 1658, 1598, 1544, 1471, 1456, 1438, 1415, 1371, 1352, 1336, 1307, 1286, 1263, 1236, 1203, 1159, 1138, 1122, 1091, 1074, 1014, 956, 914, 893, 860, 848, 748, 736, 694, 653 cm^{-1}. UV/Vis (CH_2Cl_2): λ_{max} (ε) = 265 nm (51300), 298 (39700), 517 (21500). Anal. calcd. for $C_{37}H_{31}N_3O$ (533.7): C 83.27, H 5.86, N 7.87; Found: C 83.10, H 5.95, N 7.73.

2.5. 1-Methyl-3-(3-(4-nitrophenyl)-1-phenyl-2-(1,3,3-trimethylindolin-2-ylidene)but-3-en-1-ylidene)indolin-2-one (8e)

After chromatography on silica gel (hexane/ethylacetate 4:1) compound **8e** was obtained as a violet solid (504 mg, 0.91 mmol, 91%), dr = 52:48, Mp 223 °C. ^{1}H NMR (500 MHz, $CDCl_3$): δ 1.59-1.59 (m, 6 H), 2.77 (s, 1.56 H), 3.11 (s, 1.56 H), 5.14 (s, 0.52 H), 5.79 (s, 0.52 H), 5.81 (d, J = 7.6 Hz, 0.52 H), 6.41-6.47 (m, 1 H),

6.57 (d, J = 7.7 Hz, 0.52 H), 6.62-6.69 (m, 2 H), 6.76 (d, J = 7.7 Hz, 0.52 H), 6.89-7.30 (m, 8 H), 7.45 (d, J = 8.5 Hz, 1.04 H), 7.92 (d, J = 8.7 Hz, 1.04 H); additional signals for the minor diastereomer: δ 3.21 (s, 1.44 H), 3.27 (s, 1.44 H), 4.76 (s, 0.48 H), 5.47 (s, 0.48 H), 6.20 (d, J = 7.3 Hz, 0.48 H), 6.53 (d, J = 7.4 Hz, 0.48 H), 6.90 (m, 0.48 H), 7.83 (d, J = 8.6 Hz, 0.96 H). ^{13}C NMR (125 MHz, $CDCl_3$): δ 26.5 (CH_3), 26.6 (CH_3), 36.5 (CH_3), 37.6 (CH_3), 49.7 (C_{quat}), 107.4 (CH), 107.6 (CH), 108.2 (C_{quat}), 117.5 (CH_2), 120.8 (CH), 121.7 (CH), 122.0 (CH), 122.4 (CH), 123.6 (CH), 124.3 (C_{quat}), 127.0 (CH), 128.1 (CH), 128.7 (CH), 128.9 (CH), 129.5 (CH), 132.2 (CH), 140.4 (C_{quat}), 140.5 (C_{quat}), 141.2 (C_{quat}), 144.4 (C_{quat}), 145.5 (C_{quat}), 147.0 (C_{quat}), 147.2 (C_{quat}), 148.3 (C_{quat}), 148.7 (C_{quat}), 149.7 (C_{quat}), 168.1 (C_{quat}); additional signals for the minor diastereomer: δ 50.5 (C_{quat}), 109.4 (C_{quat}), 124.7 (C_{quat}), 107.5 (CH), 117.1 (CH), 121.2 (CH), 121.9 (CH), 122.2 (CH), 122.7 (CH), 123.5 (CH_2), 126.8 (CH), 127.9 (CH), 128.5 (CH), 129.0 (CH), 130.4 (CH), 131.2 (CH), 132.6 (CH). EI MS: (70 eV, *m/z* (%)): 553 ($[M]^+$, 100), 538 ($[C_{35}H_{28}N_3O_3]^+$, 22), 173 ($[C_{10}H_7NO_2]^+$, 10), 158 ($[C_{10}H_8NO]^+$, 41), 160 ($[C_9H_6NO_2]^+$, 12). IR (KBr): $\tilde{\nu}$ = 3082, 3055, 2976, 2926, 2862, 1656, 1598, 1544, 1512, 1471, 1458, 1438, 1438, 1415, 1373, 1336, 1309, 1286, 1263, 1236, 1203, 1159, 1138, 1122, 1091, 1074, 1053, 1024, 958, 916, 893, 871, 858, 827, 767, 734, 711, 700, 678, 650, 617 cm^{-1}. UV/Vis (CH_2Cl_2): λ_{max} (ε) = 317 nm (29300), 522 (14900). Anal. calcd. for $C_{36}H_{31}N_3O_3$ (553.7): C 78.10, H 5.64, N 7.59; Found: C 77.87, H 5.89, N 7.31.

3. General Procedure for the synthesis of 4-(1,3,3-trimethylindolin-2-ylidene)but-2-en-1-ylideneindolones 10

In a flame-dried and argon-flushed Schlenk tube iodo phenylanilide **5** (1.00 mmol), alkyne **6** (1.10 mmol), and dry, degassed THF (5 mL) were placed (for experimental details see Table 2). After the addition of $PdCl_2(PPh_3)_2$ (35 mg, 0.05 mmol) and CuI (10 mg, 0.05 mmol), diisopropylethylamine (1.7 mL, 10 mmol) was added and the reaction mixture was stirred at rt for 16 h. Then, the enamine **7** (2.00 mmol) or the benzothiazolium salt **9** (2.00 mmol and 1.1 mmol of diisopropylethylamine), and EtOH (2 mL) were added. The sealed reaction vessel was placed in a thermostatted oil bath at 80 °C and stirred for 48 h. After cooling to rt the solvents were removed in vacuo and the residue was chromatographed on silica gel (hexane/EtOAc) to give the 4-(1,3,3-trimethylindolin-2-ylidene)but-2-en-1-ylideneindolones **10** as bluish-black or darkgreen solids.

Table S2: Experimental details of the three-component synthesis of the 4-(1,3,3-trimethylindolin-2-ylidene)but-2-en-1-ylideneindolones **10**.

Entry	iodo phenylanilide **5**	Alkyne **6**	Enamine **7** or benzothiazolium salt **9**	4-(1,3,3-Trimethylindolin-2-ylidene)but-2-en-1-ylidene-indolones **10** (isolated yield)
1	501 mg (1.00 mmol) of **5b**	112 mg (1.10 mmol) of **6a**	346 mg (2.00 mmol) of **7**	636 mg (98%) of **10a**
2	501 mg (1.00 mmol) of **5b**	162 mg (1.10 mmol) of **6e**	346 mg (2.00 mmol) of **7**	597 mg (90%) of **10b**
3	501 mg (1.00 mmol) of **5b**	150 mg (1.10 mmol) of **6c**	346 mg (2.00 mmol) of **7**	532 mg (78%) of **10c**
4	501 mg (1.00 mmol) of **5b**	140 mg (1.10 mmol) of **6d**	346 mg (2.00 mmol) of **7**	553 mg (82%) of **10d**
5	536 mg (1.00 mmol) of **5c**	174 mg (1.10 mmol) of 4-*t*-butyl phenylacetylene (**6f**)	346 mg (2.00 mmol) of **7**	606 mg (82%) of **10e**
6	536 mg (1.00 mmol) of **5c**	150 mg (1.10 mmol) of **6c**	346 mg (2.00 mmol) of **7**	660 mg (92%) of **10f**
7	536 mg (1.00 mmol) of **5c**	140 mg (1.10 mmol) of **6d**	346 mg (2.00 mmol) of **7**	637 mg (90%) of **10g**
8	501 mg (1.00 mmol) of **5a**	150 mg (1.10 mmol) of **6c**	582 mg (2.00 mmol) of **9**[c]	448 mg (84%) of **10h**

[c]Diisopropylamine was added for in situ generation of the *S*,*N*-ketene acetal.

3.1. (*Z*)-3-((2*Z*,4*E*)-1,3-Diphenyl-4-(1,3,3-trimethylindolin-2-ylidene)but-2-en-1-ylidene)-1-tosylindolin-2-one (10a)

After chromatography on silica gel (hexane/ethylacetate 4:1) compound **10a** was obtained as a bluish-black solid (636 mg, 0.98 mmol, 98%), Mp 141 °C. ^{1}H NMR (300 MHz, $CDCl_3$): δ 0.85 (s, 6 H), 2.30 (s, 3 H), 2.31 (s, 3 H), 4.68 (d, *J* = 1.4 Hz, 1 H), 5.70 (d, *J* = 7.3 Hz, 1 H), 6.27 (d, *J* = 7.8 Hz, 1 H), 6.56 (t, *J* = 7.8 Hz, 1 H), 6.74 (dt, *J* = 7.3, 0.7 Hz, 1 H), 6.90-7.22 (m, 12 H), 7.32 (dd, *J* = 7.7, 1.8 Hz, 2 H), 7.66 (d,

J = 1.4 Hz, 1 H), 7.80 (dd, J = 8.2, 2.2 Hz, 1 H), 7.86 (d, J = 7.8 Hz, 1 H), 7.92 (d, J = 8.4 Hz, 2 H). ^{13}C NMR (75 MHz, $CDCl_3$): δ 21.9 (CH_3), 29.4 (CH_3), 34.4 (CH_3), 46.7 (C_{quat}), 94.3 (CH), 107.4 (CH), 113.0 (CH), 118.2 (C_{quat}), 120.8 (CH), 121.8 (CH), 122.7 (CH), 123.4 (CH), 124.6 (CH), 125.4 (C_{quat}), 127.3 (CH), 127.8 (CH), 127.9 (CH), 128.1 (CH), 128.7 (CH), 128.9 (CH), 129.1 (CH), 129.3 (CH), 129.5 (CH), 129.8 (CH), 136.4 (C_{quat}), 137.1 (C_{quat}), 138.2 (C_{quat}), 140.7 (C_{quat}), 144.4 (C_{quat}), 145.1 (C_{quat}), 146.4 (C_{quat}), 153.9 (C_{quat}), 155.9 (C_{quat}), 161.8 (C_{quat}), 165.9 (C_{quat}). EI MS: (70 eV, *m/z* (%)): 648 ($[M]^+$, 4), 493 ($[C_{35}H_{29}N_2O]^+$, 6), 334 (31), 321 (11), 306 (11), 291 (12), 222 (11), 218 (37), 144 (33), 142 (47), 132 (27), 127 (22), 117 (16), 105 (53), 91 (100). IR (KBr): $\tilde{\nu}$ = 3051, 2964, 2924, 2862, 1691, 1577, 1504, 1485, 1465, 1454, 1442, 1400, 1367, 1334, 1315, 1290, 1246, 1217, 1176, 1161, 1130, 1161, 1130, 1116, 1085, 1076, 1060, 1018, 1001, 960, 943, 925, 873, 854, 815, 773, 742, 725, 686, 663, 651, 611 cm^{-1}. UV/Vis (CH_2Cl_2): λ_{max} (ε) = 290 nm (20400), 587 (33600). Anal. calcd. for $C_{42}H_{36}N_2O_3S$ (648.8): C 77.75, H 5.59, N 4.32; Found: C 77.58, H 5.41, N 4.29.

3.2. (*Z*)-3-((2*Z*,4*E*)-3-(4-Aminophenyl)-1-phenyl-4-(1,3,3-trimethylindolin-2-ylidene)but-2-en-1-ylidene)-1-tosylindolin-2-one (10b)

After chromatography on silica gel (hexane/ethylacetate 4:1) compound **10b** was obtained as a bluish black solid (597 mg, 0.90 mmol, 90%), Mp 175 °C. ^{1}H NMR (500 MHz, $CDCl_3$): δ 0.86 (s, 6 H), 2.33 (s, 3 H), 2.43 (s, 3 H), 3.77 (s, 2 H), 4.66 (s, 1 H), 5.63 (d, J = 7.7 Hz, 1 H), 6.33 (d, J = 7.8 Hz, 1 H), 6.51 (d, J = 8.5 Hz, 2 H), 6.57 (t, J = 8.2 Hz, 1), 6.77 (t, J = 7.4 Hz, 1 H), 6.95 (d, J = 7.4 Hz, 1 H), 6.99 (t, J = 8.4 Hz, 2 H), 7.03 (dt, J = 7.8, 1.0 Hz, 2 H), 7.17 (d, J = 8.5 Hz, 2 H), 7.19 (s, 1 H), 7.20-7.24 (m, 4 H), 7.69 (d, J = 1.2 Hz, 1 H), 7.88 (d, J = 8.1 Hz, 1 H), 7.95 (d, J = 8.3 Hz, 2 H). ^{13}C NMR (125 MHz, $CDCl_3$): δ 22.1 (CH_3), 29.6 (CH_3), 34.6 (CH_3), 46.9 (C_{quat}), 95.0 (CH), 107.5 (CH), 113.1 (CH), 115.3 (CH), 116.8 (C_{quat}), 120.9 (CH), 122.0 (CH), 122.6 (CH), 122.9 (CH), 123.5 (CH), 126.1 (C_{quat}), 126.9 (CH), 128.0 (CH), 128.3 (CH), 129.0 (CH), 129.6 (CH), 129.7 (CH), 130.0 (CH), 130.7 (CH), 134.6 (C_{quat}), 136.8 (C_{quat}), 136.9 (C_{quat}), 138.5 (C_{quat}), 141.2 (C_{quat}), 145.2 (C_{quat}), 146.7 (C_{quat}), 148.1 (C_{quat}), 155.1 (C_{quat}), 156.9 (C_{quat}), 162.2 (C_{quat}), 166.1 (C_{quat}). EI MS: (70 eV, *m/z* (%)): 663 ($[M]^+$, 21), 376 (17), 361 (55), 349 (21), 287

(16), 180 (21), 173 (43), 158 (100). IR (KBr): $\tilde{\nu}$ = 3375, 2960, 2920, 2362, 1631, 1602, 1573, 1467, 1438, 1419, 1367, 1332, 1317, 1292, 1269, 1246, 1159, 1122, 1074, 1056, 1020, 981, 960, 943, 923, 885, 862, 812, 771, 740, 702, 688, 659, 640, 612 cm^{-1}. UV/Vis (CH_2Cl_2): λ_{max} (ε) = 276 nm (51400), 592 (57500). Anal. calcd. for $C_{42}H_{37}N_3O_3S$ (663.8): C 75.99, H 5.62, N 6.33; Found: C 75.97, H 5.58, N 6.19.

3.3. (*Z*)-3-((2Z,4*E*)-3-(4-Chlorphenyl)-1-phenyl-4-(1,3,3-trimethylindolin-2-yliden)but-2-en-1-yliden)-1-tosylindolin-2-on (10c)

After chromatography on silica gel (hexane/ethylacetate 4:1) compound **10c** was obtained as a bluish black solid (532 mg, 0.78 mmol, 78%), Mp 143 °C. ^{1}H NMR (500 MHz, $CDCl_3$): δ 0.91 (s, 6 H), 2.39 (s, 3 H), 2.44 (s, 3 H), 4.71 (d, *J* = 1.5 Hz, 1 H), 5.78 (d, *J* = 7.4 Hz, 1 H), 6.39 (d, *J* = 7.8 Hz, 1 H), 6.65 (t, *J* = 7.8 Hz, 1 H), 6.83 (dt, *J* = 7.4, 0.7 Hz, 1 H), 7.00 (d, *J* = 7.4 Hz, 1 H), 7.08 (t, *J* = 7.7 Hz, 2 H), 7.22-7.36 (m, 11 H), 7.71 (d, *J* = 1.5 Hz, 1 H), 7.93 (d, *J* = 8.0 Hz, 1 H), 8.00 (d, *J* = 8.4 Hz, 2 H). ^{13}C NMR (75 MHz, $CDCl_3$): δ 21.9 (CH_3), 29.4 (CH_3), 34.6 (CH_3), 46.8 (C_{quat}), 93.7 (CH), 107.5 (CH), 113.1 (CH), 118.8 (C_{quat}), 121.0 (CH), 121.8 (CH), 122.9 (CH), 123.5 (CH), 124.8 (CH), 125.2 (C_{quat}), 127.7 (CH), 127.9 (CH), 128.1 (CH), 128.9 (CH), 129.0 (CH), 129.3 (CH), 129.6 (CH), 129.9 (CH), 130.1 (CH), 134.9 (C_{quat}), 136.4 (C_{quat}), 137.3 (C_{quat}), 138.2 (C_{quat}), 140.5 (C_{quat}), 142.9 (C_{quat}), 145.2 (C_{quat}), 146.3 (C_{quat}), 152.0 (C_{quat}), 155.5 (C_{quat}), 161.9 (C_{quat}), 165.9 (C_{quat}). EI MS: (70 eV, *m/z* (%)): 684 ([^{37}Cl-M]$^+$, 9), 682 ([^{35}Cl-M]$^+$, 37), 527 ([$C_{35}H_{28}ClN_2O$]$^+$, 5), 380 ([$C_{25}H_{12}ClNO$]$^+$, 100). IR (KBr): $\tilde{\nu}$ = 2960, 2926, 2868, 1703, 1581, 1514, 1487, 1463, 1454, 1442, 1377, 1336, 1317, 1292, 1247, 1176, 1159, 1116, 1085, 1076, 1056, 1018, 1012, 958, 939, 925, 871, 777, 815, 744, 732, 721, 704, 683, 657, 632, 611 cm^{-1}. UV/Vis (CH_2Cl_2): λ_{max} (ε) = 257 nm (66400), 557 (30200). HRMS calcd. for $C_{42}H_{35}{}^{35}ClN_2O_3S$: 682.2057; Found: 682.2051. Anal. calcd. for $C_{42}H_{35}ClN_2O_3S$ (683.3): C 73.83, H 5.16, N 4.10; Found: C 73.63, H 5.45, N 3.87.

3.4. 4-((1*Z*,2*Z*,4*E*)-4-(2-Oxo-1-tosylindolin-3-ylidene)-4-phenyl-1-(1,3,3-trimethyl-indolin-2-ylidene)but-2-en-2-yl)benzonitrile (10d)

After chromatography on silica gel (hexane/ethylacetate 4:1) compound **10d** was obtained as a bluish black solid (553 mg, 0.82 mmol, 82%), Mp 233 °C. ^{1}H NMR (300 MHz, $CDCl_3$): δ 0.91 (s, 6 H), 2.39 (s, 3 H), 2.41 (s, 3 H), 4.71 (s, 1 H), 5.78 (d, *J* = 7.8 Hz, 1 H), 6.39 (d, *J* = 7.8 Hz, 1 H), 6.64 (t, *J* = 7.7 Hz, 1 H), 6.83 (t, *J* = 7.4 Hz, 1 H), 7.00 (d, *J* = 7.2 Hz, 1 H), 7.08 (t, *J* = 7.7 Hz, 2 H), 7.2-7.4 (m, 11 H), 7.70 (s, 1 H), 7.93 (d, *J* = 8.2 Hz, 1 H), 8.00 (d, *J* = 8.00 Hz, 2 H). ^{13}C NMR (75 MHz, $CDCl_3$): δ 21.9 (CH_3), 29.4 (CH_3), 34.7 (CH_3), 46.8 (C_{quat}), 92.8 (CH), 107.7 (CH), 112.1 (C_{quat}), 113.2 (CH), 119.0 (C_{quat}), 120.1 (C_{quat}), 121.2 (CH), 121.8 (CH), 123.2 (CH), 123.7 (CH), 124.8 (C_{quat}), 126.3 (CH), 128.0 (CH), 128.1 (CH), 128.2 (CH), 129.2 (CH), 129.3 (CH), 129.6 (CH), 129.7 (CH), 129.9 (CH), 132.5 (CH), 136.2 (C_{quat}), 137.6 (C_{quat}), 139.9 (C_{quat}), 140.1 (C_{quat}), 145.3 (C_{quat}), 146.2 (C_{quat}), 149.1 (C_{quat}), 150.3 (C_{quat}), 154.6 (C_{quat}), 162.0 (C_{quat}), 166.0 (C_{quat}). EI MS: (70 eV, *m/z* (%)): 673 ($[M]^+$, 45), 518 ($[C_{36}H_{28}N_3O]^+$, 16), 371 (15), 172 (20), 159 (100). IR (KBr): $\tilde{\nu}$ = 2980, 2972, 2887, 2225, 1714, 1624, 1591, 1539, 1523, 1498, 1463, 1456, 1442, 1425, 1379, 1363, 1340, 1290, 1244, 1213, 1176, 1159, 1126, 1114, 1083, 1072, 1054, 1020, 1006, 968, 954, 939, 927, 858, 900, 840, 815, 779, 752, 706, 684, 657, 644, 617 cm^{-1}. UV/Vis (CH_2Cl_2): λ_{max} (ε) = 269 nm (36100), 375 (15200), 592 (34000). Anal. calcd. for $C_{43}H_{35}N_3O_3S$ (673.8): C 76.65, H 5.24, N 6.24; Found: C 76.43, H 5.37, N 6.08.

3.5. (*Z*)-3-((2*Z*,4*E*)-3-(4-(*tert*-Butyl)phenyl)-1-(4-chlorophenyl)-4-(1,3,3-trimethyl-indolin-2-ylidene)but-2-en-1-ylidene)-1-tosylindolin-2-one (10e)

After chromatography on silica gel (hexane/ethylacetate 4:1) compound **10e** was obtained as a bluish black solid (606 mg, 0.82 mmol, 82%), Mp 174 °C. ^{1}H NMR (300 MHz, $CDCl_3$): δ 0.98 (s, 6 H), 1.30 (s, 9 H), 2.39 (s, 3 H), 2.42 (s, 3 H), 4.67 (d, *J* = 1.3 Hz, 1 H), 5.91 (d, *J* = 7.4 Hz, 1 H), 6.39 (d, *J* = 7.8 Hz, 1 H), 6.70 (dt, 8.0, 1.0 Hz, 1 H), 6.85 (dt, *J* = 7.4, 0.7 Hz, 1 H), 7.03 (dd, 7.4, 0.7 Hz, 1 H), 7.06-7.13 (m, 2 H), 7.25-7.35 (m, 10 H), 7.71 (d, *J* = 1.3 Hz, 1 H), 7.94 (d, *J* = 8.0 Hz, 1 H), 7.99 (d,

J = 8.4 Hz, 2 H). ^{13}C NMR (75 MHz, CDCl$_3$): δ 21.9 (CH$_3$), 29.2 (CH$_3$), 31.5 (CH$_3$), 34.3 (CH$_3$), 34.9 (C$_{quat}$), 46.9 (C$_{quat}$), 94.4 (CH), 107.4 (CH), 133.2 (CH), 117.7 (C$_{quat}$), 121.0 (CH), 121.9 (CH), 122.5 (CH), 123.5 (CH), 124.0 (CH), 125.2 (C$_{quat}$), 125.7 (CH), 127.5 (CH), 127.8 (CH), 128.1 (CH), 128.7 (CH), 129.7 (CH), 129.9 (CH), 131.0 (CH), 135.1 (C$_{quat}$), 136.4 (C$_{quat}$), 137.1 (C$_{quat}$), 138.2 (C$_{quat}$), 138.9 (C$_{quat}$), 141.4 (C$_{quat}$), 145.1 (C$_{quat}$), 146.4 (C$_{quat}$), 152.8 (C$_{quat}$), 154.2 (C$_{quat}$), 154.4 (C$_{quat}$), 162.3 (C$_{quat}$), 165.8 (C$_{quat}$). EI MS: (70 eV, *m/z* (%)): 738 ([^{35}Cl-M]$^+$, 3), 583 ([C$_{39}$H$_{36}$^{35}ClN$_2$O]$^+$, 2), 566 ([C$_{34}$H$_{29}$^{35}ClNO$_3$S]$^+$, 2), 453 ([C$_{31}$H$_{32}$^{35}ClN]$^+$, 100), 436 (92), 431 (11), 420 (22), 345 (13), 343 (11), 317 ([C$_{23}$H$_{27}$N]$^+$12), 287 (37), 233 (11), 223 (10), 220 (10), 211 (15), 186 (17), 173 (23). IR (KBr): $\tilde{\nu}$ = 2962, 2943, 2864, 1697, 1581, 1506, 1483, 1469, 1452, 1365, 1332, 1317, 1290, 1271, 1247, 1168, 1157, 1118, 1083, 1058, 1020, 962, 939, 883, 831, 831, 831, 831, 831, 831, 831, 667, 650, 650, 628 cm^{-1}. UV/Vis (CH$_2$Cl$_2$): λ_{max} (ε) = 266 nm (37300), 373 (40300), 597 (50900). Anal. calcd. for C$_{46}$H$_{43}$ClN$_2$O$_3$S (739.4): C 74.73, H 5.86, N 3.79; Found: C 74.82, H 5.95, N 3.83.

3.6. (*Z*)-3-((2*Z*,4*E*)-1,3-Bis(4-chlorophenyl)-4-(1,3,3-trimethylindolin-2-ylidene)but-2-en-1-ylidene)-1-tosylindolin-2-one (10f)

After chromatography on silica gel (hexane/ethylacetate 4:1) compound **10f** was obtained as a bluish black solid (660 mg, 0.92 mmol, 92%), Mp 139 °C. ^{1}H NMR (500 MHz, CDCl$_3$): δ 0.98 (s, 6 H), 2.39 (s, 3 H), 2.44 (s, 3 H), 4.64 (s, 1 H), 5.94 (d, *J* = 7.7 Hz, 1 H), 6.41 (d, *J* = 7.7 Hz, 1 H), 6.71 (t, *J* = 7.3 Hz, 1 H), 6.83 (d, *J* = 7.8 Hz, 1 H), 6.86 (t, *J* = 7.2 Hz, 1 H), 7.04-7.05 (m, 2 H), 7.08-7.13 (m, 3 H), 7.19 (d, *J* = 6.6 Hz, 1 H), 7.24-7.32 (m, 6 H), 7.67 (s, 1 H), 7.95 (d, *J* = 8.1 Hz, 1 H), 7.99 (d, *J* = 8.3 Hz, 2 H). ^{13}C NMR (125 MHz, CDCl$_3$): δ 21.9 (CH$_3$), 29.2 (CH$_3$), 34.7 (CH$_3$), 46.9 (C$_{quat}$), 93.6 (CH), 107.7 (CH), 108.2 (CH), 113.2 (CH), 118.7 (C$_{quat}$), 121.2 (CH), 121.9 (CH), 122.5 (CH), 122.7 (CH), 122.8 (CH), 123.7 (CH), 124.4 (CH), 124.9 (C$_{quat}$), 127.9 (CH), 128.0 (CH), 129.0 (CH), 129.9 (CH), 130.9 (CH), 135.0 (C$_{quat}$), 135.3 (C$_{quat}$), 136.3 (C$_{quat}$), 137.4 (C$_{quat}$), 138.1 (C$_{quat}$), 138.7 (C$_{quat}$), 142.8 (C$_{quat}$), 145.3 (C$_{quat}$), 146.2 (C$_{quat}$), 152.1 (C$_{quat}$), 153.7 (C$_{quat}$), 162.4 (C$_{quat}$), 165.8 (C$_{quat}$). EI MS: (70 eV, *m/z* (%)): 716 ([^{35}Cl^{35}Cl-M]$^+$, 2), 429 ([C$_{27}$H$_{21}$^{35}Cl$_2$N]$^+$, 25), 287 ([C$_{21}$H$_{21}$N]$^+$, 19), 200 ([C$_{14}$H$_{18}$N]$^+$, 14), 186 ([C$_{13}$H$_{16}$N, 25), 173 (C$_{12}$H$_{15}$N,

25), 160 (100), 146 (35), 132 (37). IR (KBr): $\tilde{\nu}$ = 3053, 2962, 2926, 2860, 1707, 1695, 1577, 1483, 1465, 1454, 1379, 1334, 1290, 1315, 1244, 1176, 1159, 1126, 1118, 1085, 1074, 1056, 1010, 939, 923, 869, 827, 815, 777, 744, 705, 678, 655 cm^{-1}. UV/Vis (CH_2Cl_2): λ_{max} (ε) = 591 nm (28700). Anal. calcd. for $C_{42}H_{34}Cl_2N_2O_3S$ (717.7): C 70.29, H 4.77, N 3.90; Found: C 70.02, H 5.00, N 3.82.

3.7. 4-((1*Z*,2*Z*,4*E*)-4-(4-Chlorophenyl)-4-(2-oxo-1-tosylindolin-3-ylidene)-1-(1,3,3-tri-methylindolin-2-ylidene)but-2-en-2-yl)benzonitrile (10g)

After chromatography on silica gel (hexane/ethylacetate 4:1) compound **10g** was obtained as a bluish black solid (637 mg, 0.90 mmol, 90%), Mp 191 °C. ^{1}H NMR (300 MHz, $CDCl_3$): δ 0.99 (s, 6 H), 2.39 (s, 3 H), 2.42 (s, 3 H), 4.63 (d, *J* = 1.5 Hz, 1 H), 5.99 (d, *J* = 7.3 Hz, 1 H), 6.40 (d, *J* = 7.7 Hz, 1 H), 6.73 (dt, *J* = 8.0, 1.1 Hz, 1 H), 6.88 (dt, *J* = 7.4, 1.0 Hz, 1 H), 7.04 (dd, *J* = 7.4, 0.8 Hz, 1 H), 7.08-7.13 (m, 6 H), 7.25-7.36 (m, 6 H), 7.70 (d, *J* = 1.5 Hz, 1 H), 7.95 (d, *J* = 8.5 Hz, 1 H), 7.99 (d, *J* = 8.4 Hz, 2 H). ^{13}C NMR (75 MHz, $CDCl_3$): δ 21.9 (CH_3), 29.2 (CH_3), 34.7 (CH_3), 46.9 (C_{quat}), 92.7 (CH), 107.8 (CH), 112.2 (C_{quat}), 113.3 (CH), 119.0 (C_{quat}), 120.1 (C_{quat}), 121.5 (CH), 121.9 (CH), 123.0 (CH), 123.8 (CH), 124.6 (C_{quat}), 125.9 (CH), 128.0 (CH), 128.1 (CH), 128.5 (CH), 129.3 (CH), 129.9 (CH), 130.0 (CH), 130.8 (CH), 132.5 (CH), 135.5 (C_{quat}), 136.1 (C_{quat}), 137.7 (C_{quat}), 137.9 (C_{quat}), 138.3 (C_{quat}), 145.4 (C_{quat}), 146.0 (C_{quat}), 148.9 (C_{quat}), 150.4 (C_{quat}), 152.8 (C_{quat}), 162.4 (C_{quat}), 165.8 (C_{quat}). EI MS: (70 eV, *m/z* (%)): 532 ($[M-C_{12}H_{14}N]^+$, 5), 370 (21), 269 (15), 248 (13), 126 (15), 163 (14), 148 (100), 128 (40). IR (KBr): $\tilde{\nu}$ = 3012, 2960, 2918, 2856, 2227, 1732, 1708, 1587, 1532, 1517, 1487, 1469, 1454, 1392, 1363, 1334, 1288, 1244, 1172, 1165, 1132, 1116, 1085, 1062, 1047, 1018, 977, 958, 941, 921, 896, 856, 827, 817, 781, 738, 723, 704, 684, 657, 649 cm^{-1}. UV/Vis (CH_2Cl_2): λ_{max} (ε) = 276 nm (40700), 376 (20400), 597 (39400). Anal. calcd. for $C_{43}H_{34}ClN_3O_3S$ (708.3): C 72.95, H 4.84, N 5.93; Found: C 72.66, H 5.09, N 5.75.

3.8. (*Z*)-3-((2*Z*,4*Z*)-3-(4-Chlorophenyl)-4-(3-methylbenzo[*d*]thiazol-2(3*H*)-ylidene)-1-phenylbut-2-en-1-ylidene)-1-methylindolin-2-one (10h)

After chromatography on silica gel (hexane/ethylacetate 4:1) compound **10h** was obtained as a darkgreen solid (448 mg, 0.84 mmol, 84%), Mp 244 °C. ^{1}H NMR (300 MHz, $CDCl_3$): δ 2.41 (s, 3 H), 3.05 (s, 3 H), 4.83 (s, 1 H), 5.59 (s, 1 H), 6.25 (t, *J* = 7.7 Hz, 1 H), 6.33 (t, *J* = 7.7 Hz, 1 H), 6.47-6.94 (m, 11 H), 7.13 (d, *J* = 8.4 Hz, 2 H), 7.18 (d, *J* = 8.4 Hz, 2 H). ^{13}C NMR (75 MHz, $CDCl_3$): δ 25.9 (CH_3), 31.3 (CH_3), 93.7 (CH), 120.0 (C_{quat}), 121.0 (CH), 121.3 (CH), 122.1 (CH), 122.5 (CH), 124.3 (C_{quat}), 125.7 (C_{quat}), 126.1 (CH), 126.9 (CH), 128.1 (CH), 128.2 (CH), 128.3 (CH), 129.0 (CH), 129.1 (CH), 129.5 (CH), 131.1 (CH), 131.8 (CH), 134.7 (C_{quat}), 140.6 (C_{quat}), 141.8 (C_{quat}), 141.9 (C_{quat}), 142.0 (C_{quat}), 149.5 (C_{quat}), 150.9 (C_{quat}), 152.4 (C_{quat}), 168.2 (C_{quat}). EI MS: (70 eV, *m/z* (%)): 535 ([^{37}Cl-M]$^+$, 1) 533 ([^{35}Cl-M]$^+$, 3), 420 (11), 405 (18), 256 (12), 171 (35), 159 (100), 132 (21). IR (KBr): $\tilde{\nu}$ = 3055, 2981, 2881, 1658, 1544, 1494, 1469, 1456, 1431, 1354, 1340, 1325, 1288, 1240, 1186, 1166, 1155, 1120, 1093, 1074, 1041, 1016, 997, 873, 852, 831, 812, 786, 775, 759, 744, 731, 700, 667, 628, 615 cm^{-1}. UV/Vis (CH_2Cl_2): λ_{max} (ε) = 345 nm (24500), 563 (68900). Anal. calcd. for $C_{33}H_{25}ClN_2OS$ (553.1): C 74.35, H 4.73, N 5.25; Found: C 74.16, H 4.88, N 5.25.

4. ^{1}H and ^{13}C NMR Spectra of the Compounds 8

4.1. 3-(1,3-Diphenyl-2-(1,3,3-trimethylindolin-2-ylidene)but-3-en-1-ylidene)-1-methylindolin-2-one (8a)

^{1}H NMR (500 MHz, $CDCl_3$, T = 298 K)

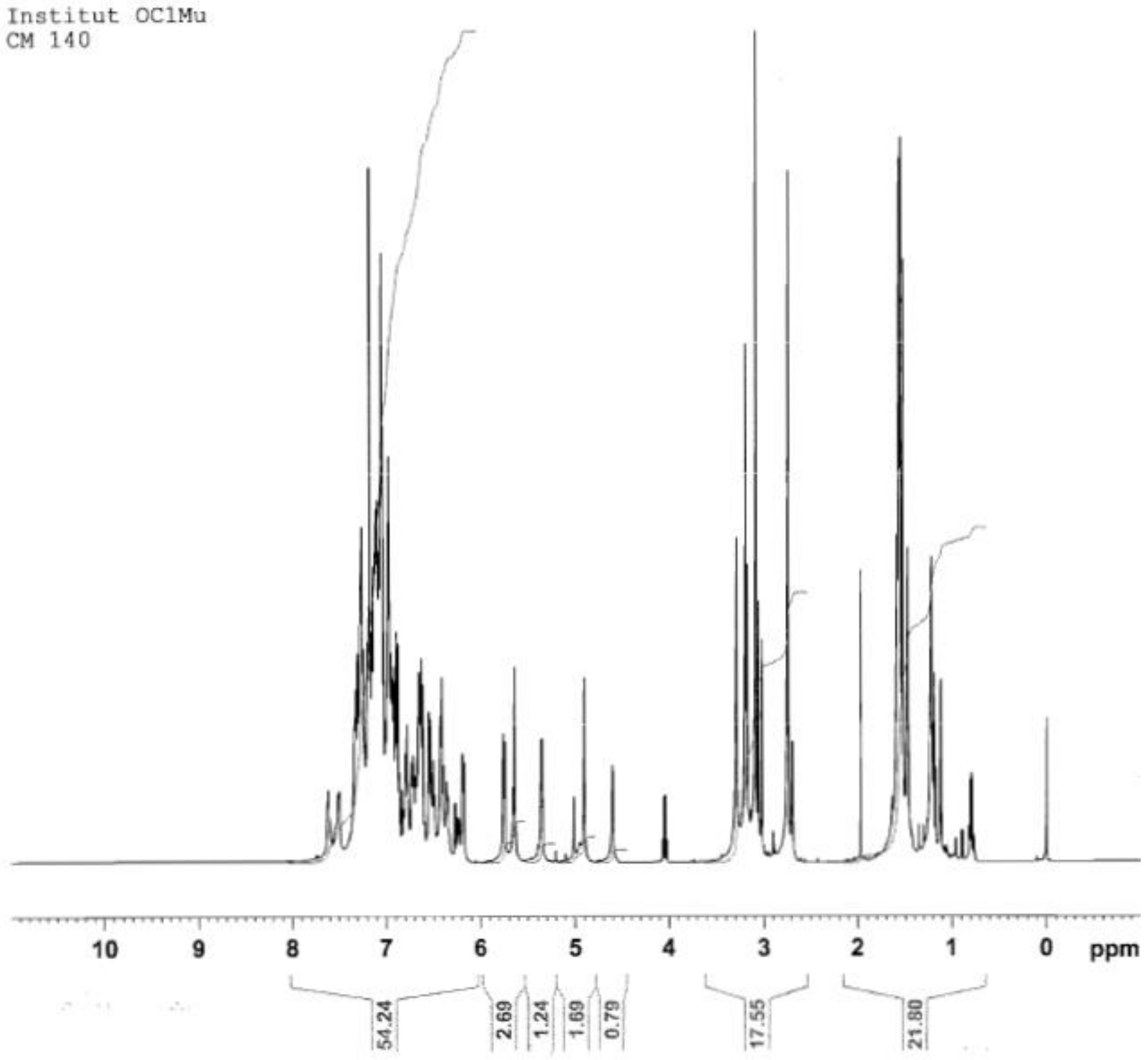

^{13}C NMR (125 MHz, $CDCl_3$, T = 298 K)

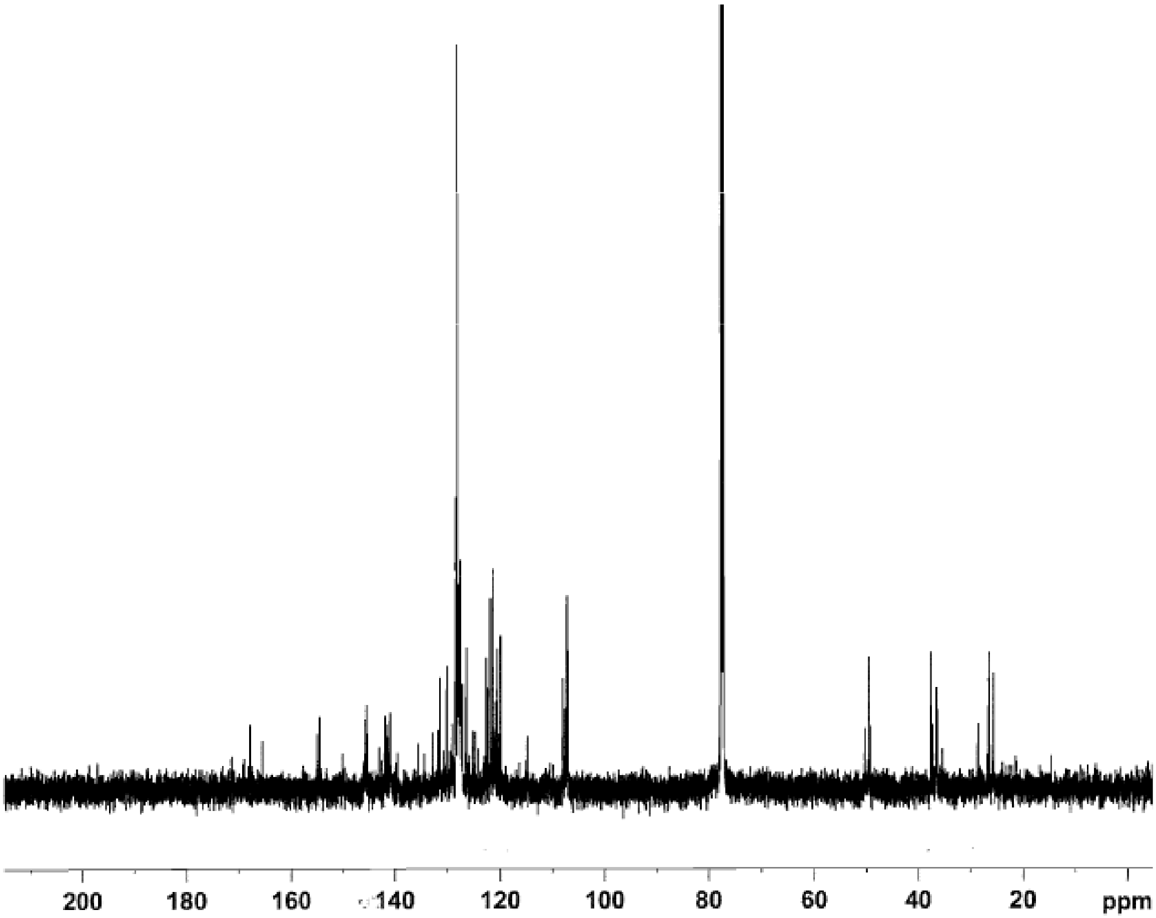

4.2. 3-(3-(4-Methoxyphenyl)-1-phenyl-2-(1,3,3-trimethylindolin-2-ylidene)but-3-en-1-ylidene)-1-methylindolin-2-one (8b)

^{1}H NMR (500 MHz, $CDCl_3$, T = 298 K)

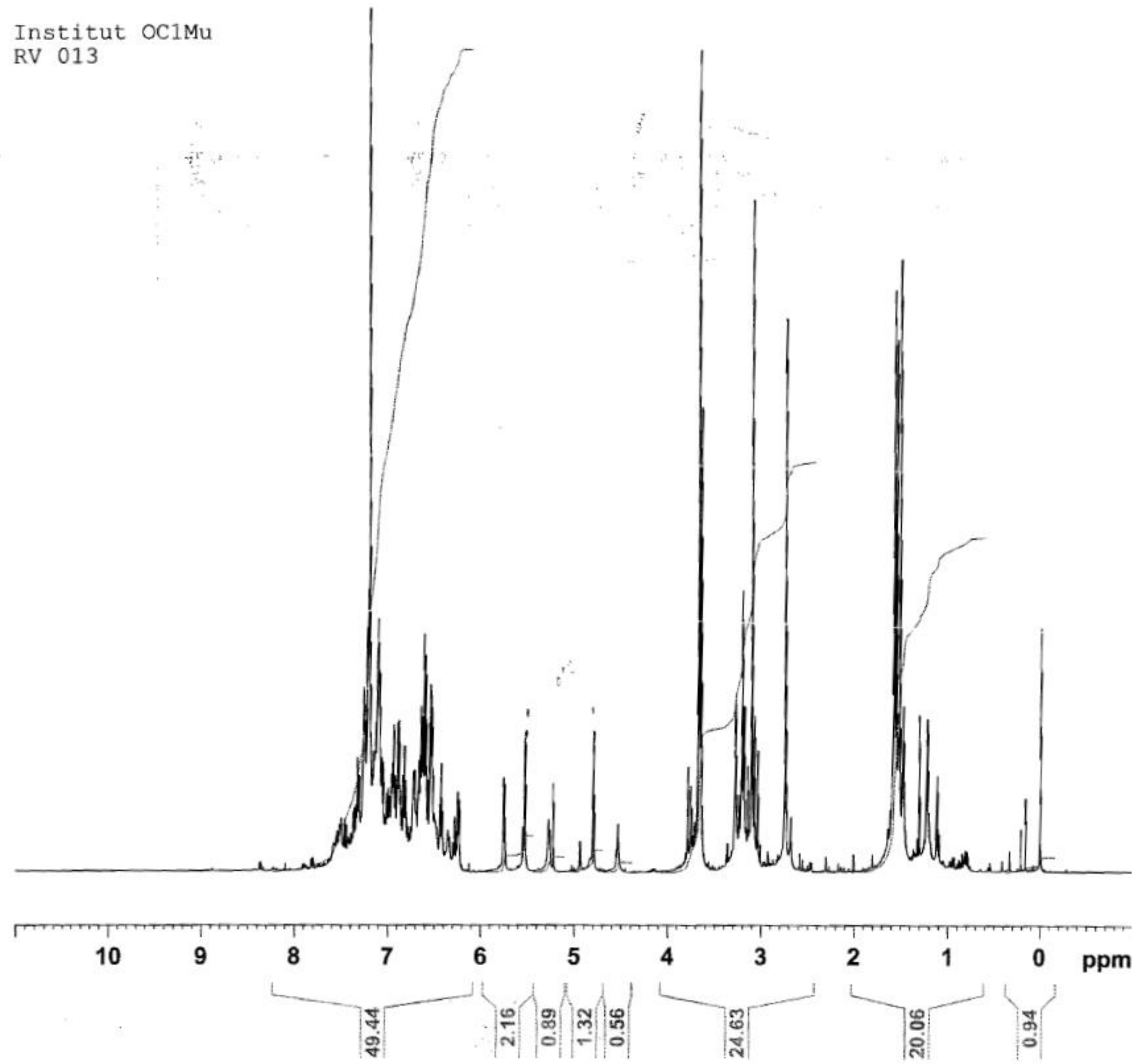

^{13}C NMR (125 MHz, $CDCl_3$, T = 298 K)

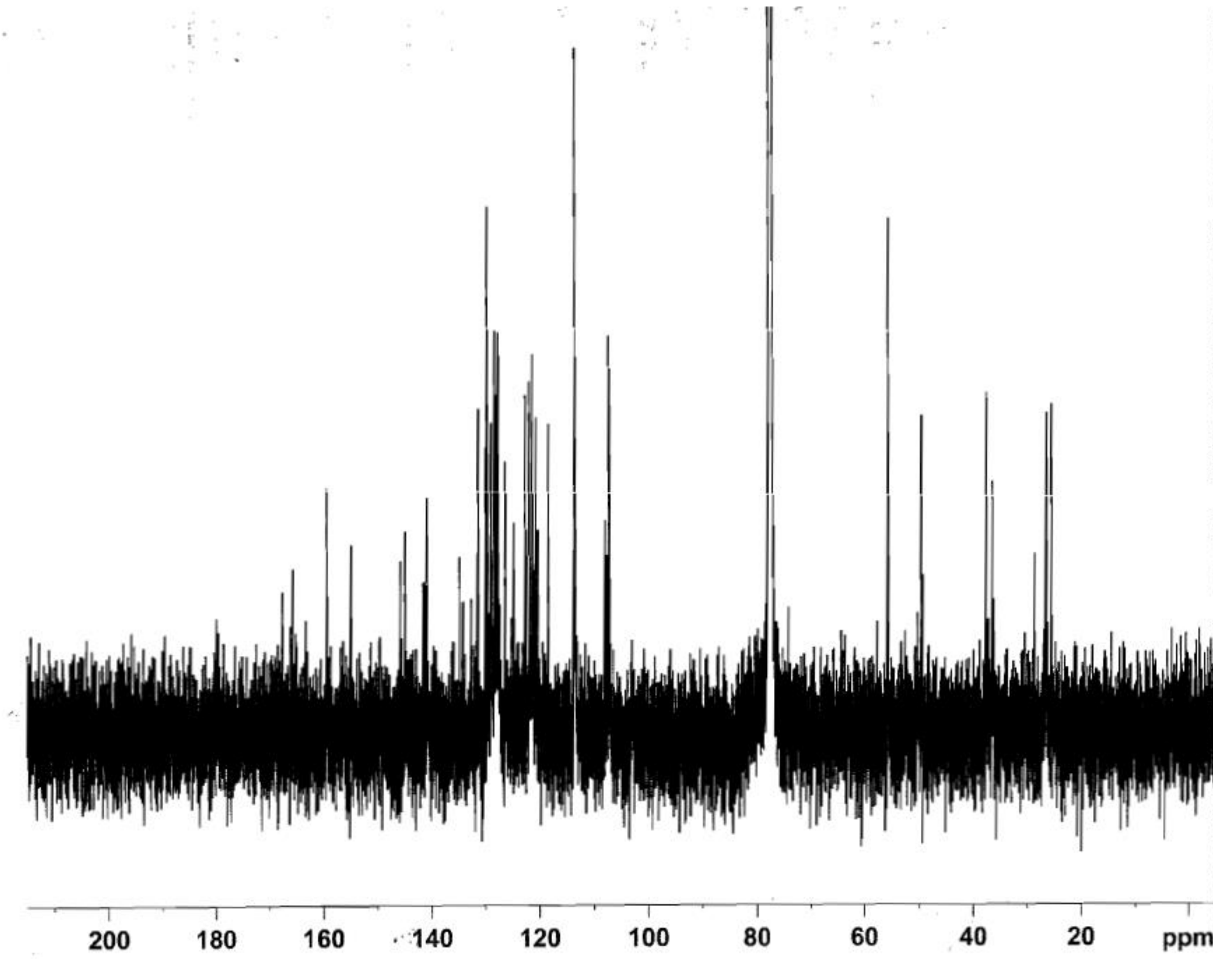

4.3. 3-(3-(4-Chlorophenyl)-1-phenyl-2-(1,3,3-trimethylindolin-2-ylidene)but-3-en-1-ylidene)-1-methylindolin-2-one (8c)

^{1}H NMR (300 MHz, $CDCl_3$, T = 298 K)

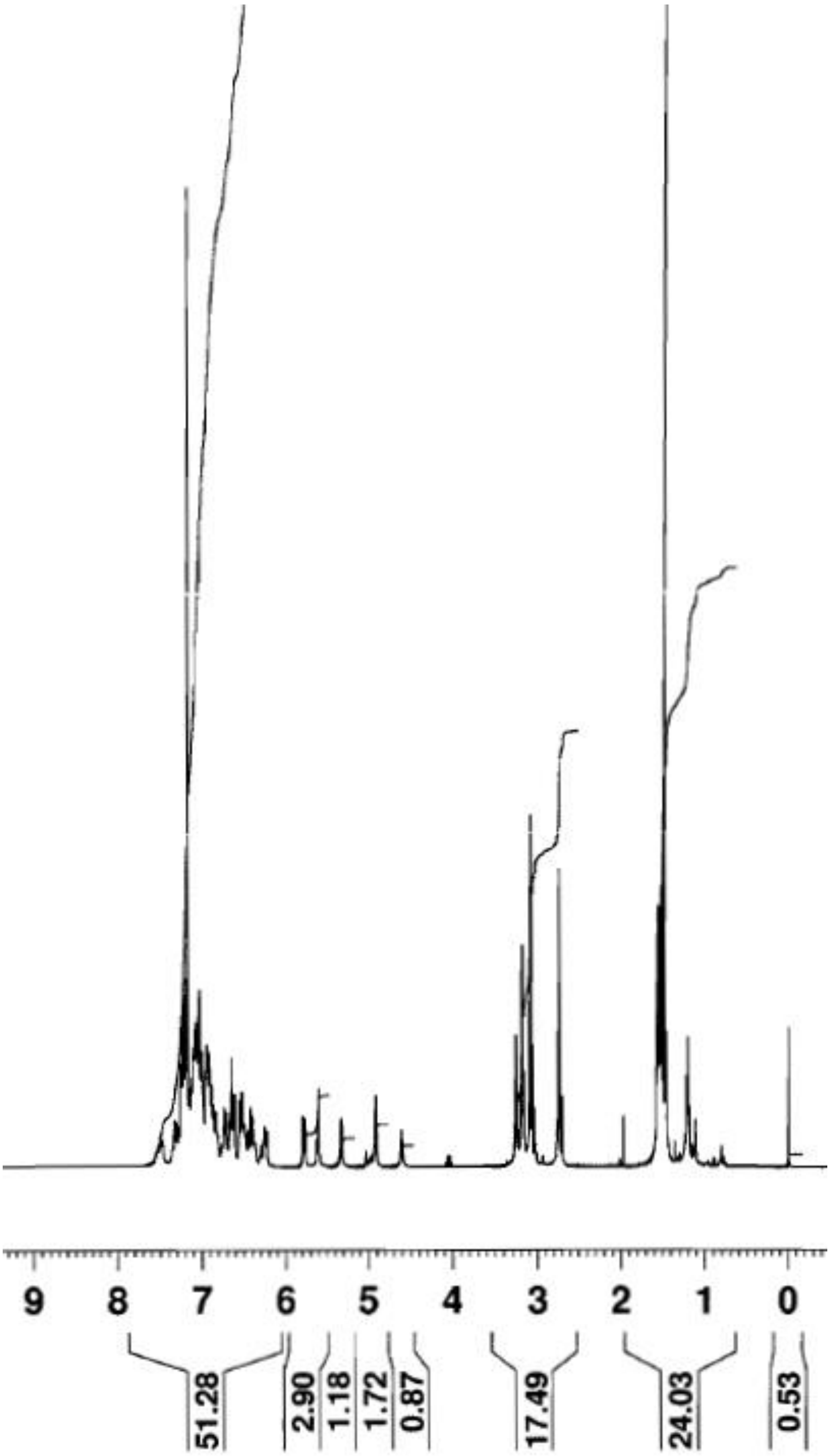

^{13}C NMR (75 MHz, $CDCl_3$, T = 298 K)

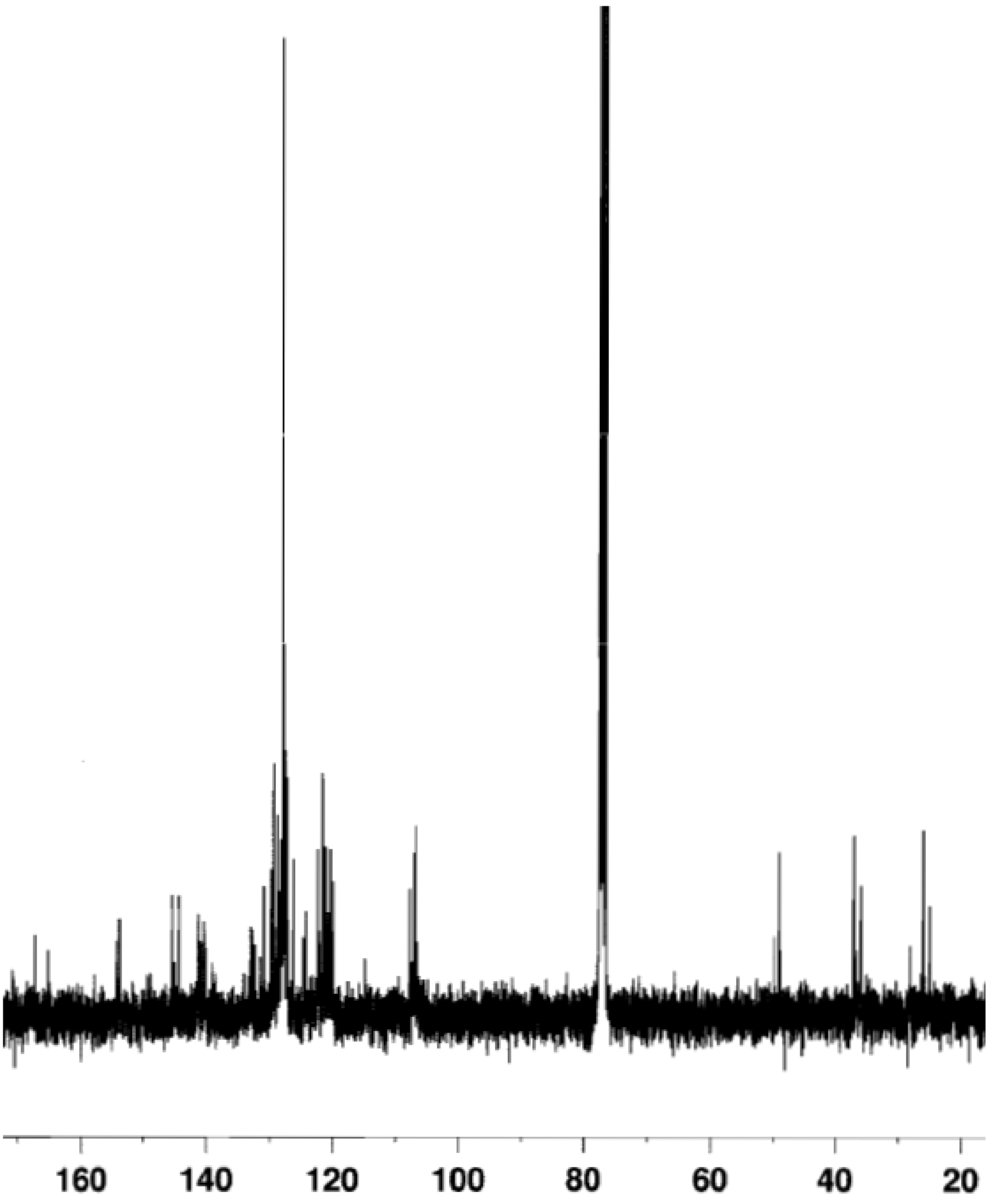

4.4. 4-(4-(1-Methyl-2-oxoindolin-3-ylidene)-4-phenyl-3-(1,3,3-trimethylindolin-2-ylidene)but-1-en-2-yl)benzonitrile (8d)

^{1}H NMR (500 MHz, $CDCl_3$, T = 298 K)

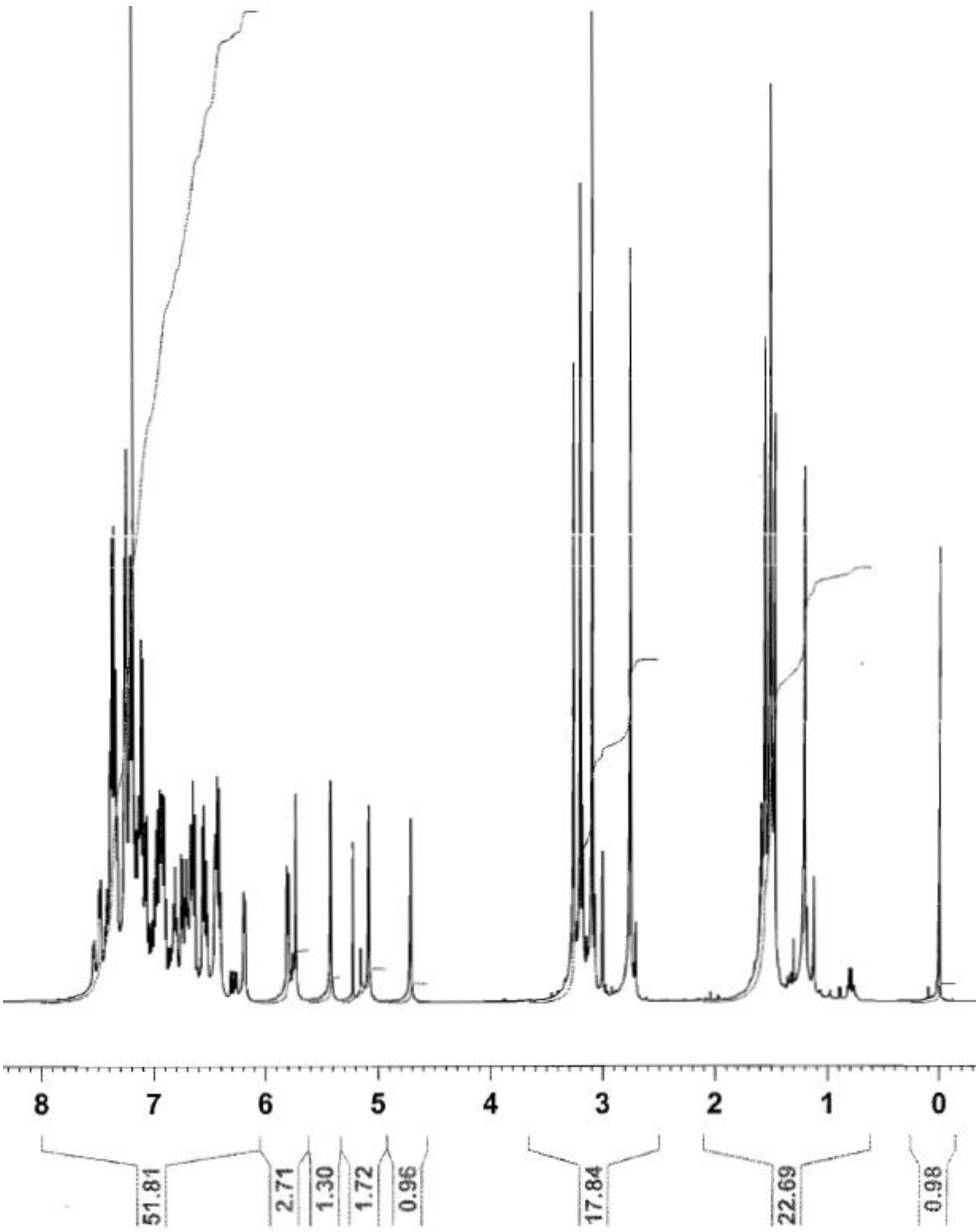

^{13}C NMR (125 MHz, $CDCl_3$, T = 298 K)

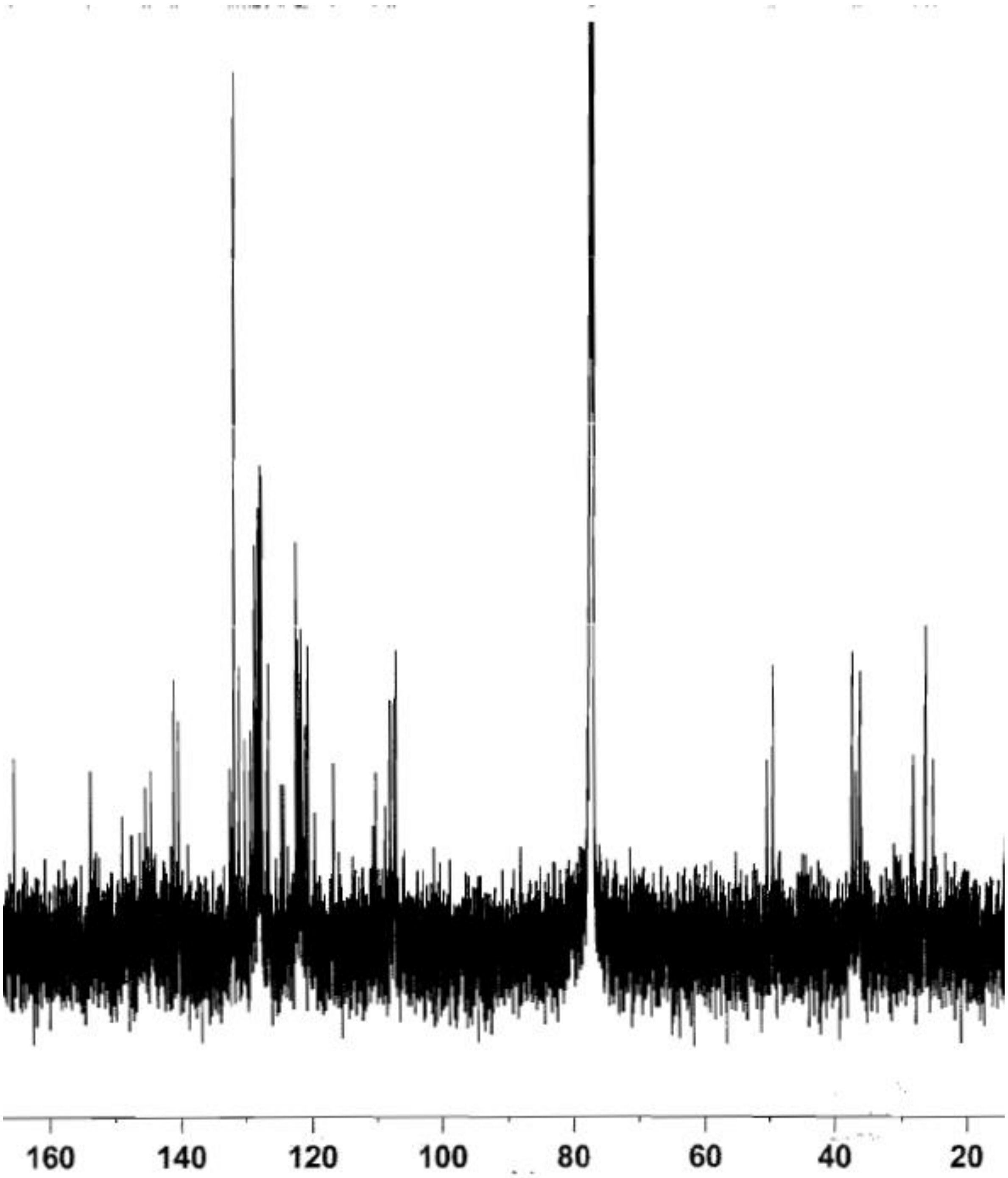

4.5. 1-Methyl-3-(3-(4-nitrophenyl)-1-phenyl-2-(1,3,3-trimethylindolin-2-ylidene)but-3-en-1-ylidene)indolin-2-one (8e)

^{1}H NMR (500 MHz, $CDCl_3$, T = 298 K)

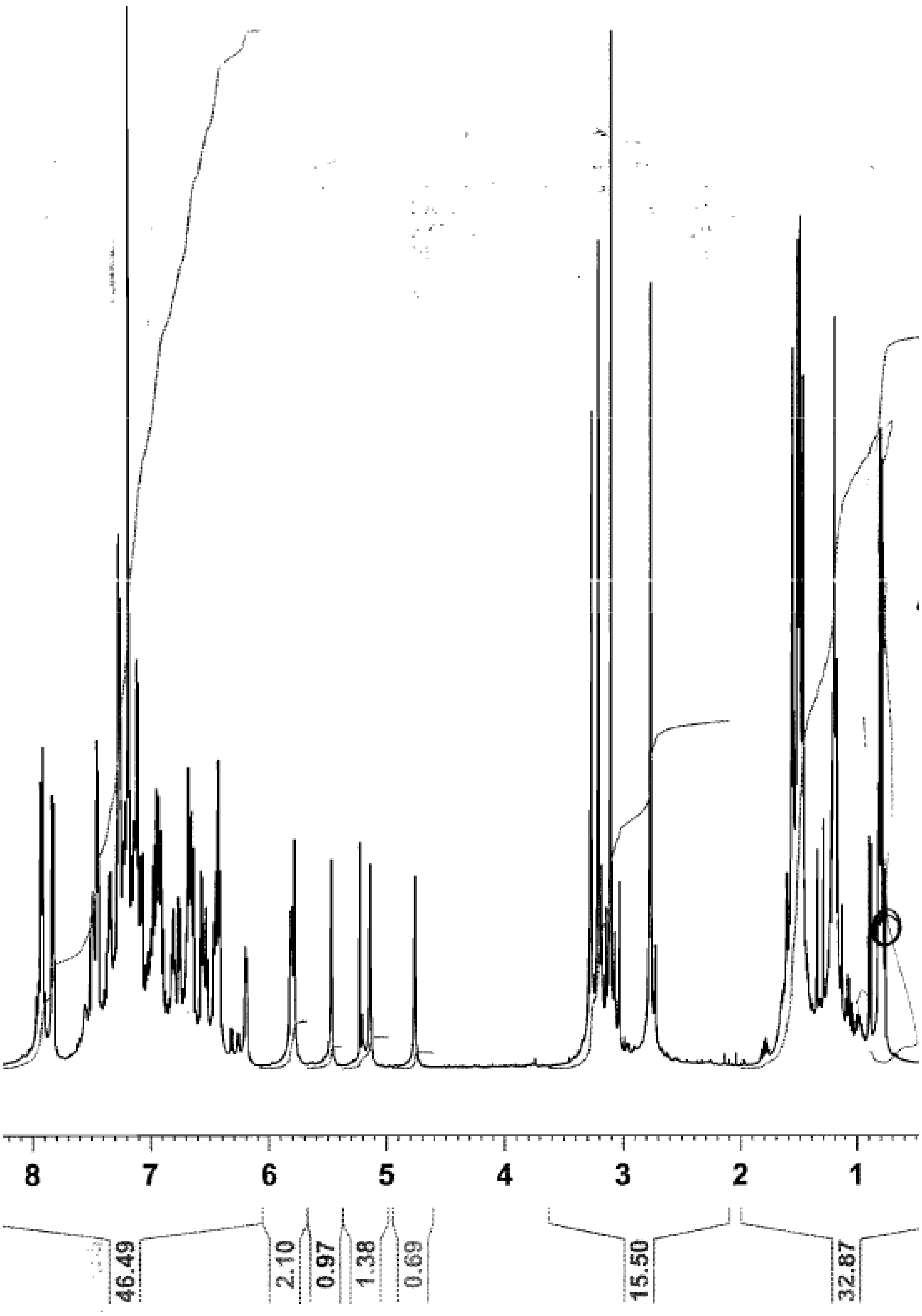

^{13}C NMR (125 MHz, $CDCl_3$, T = 298 K)

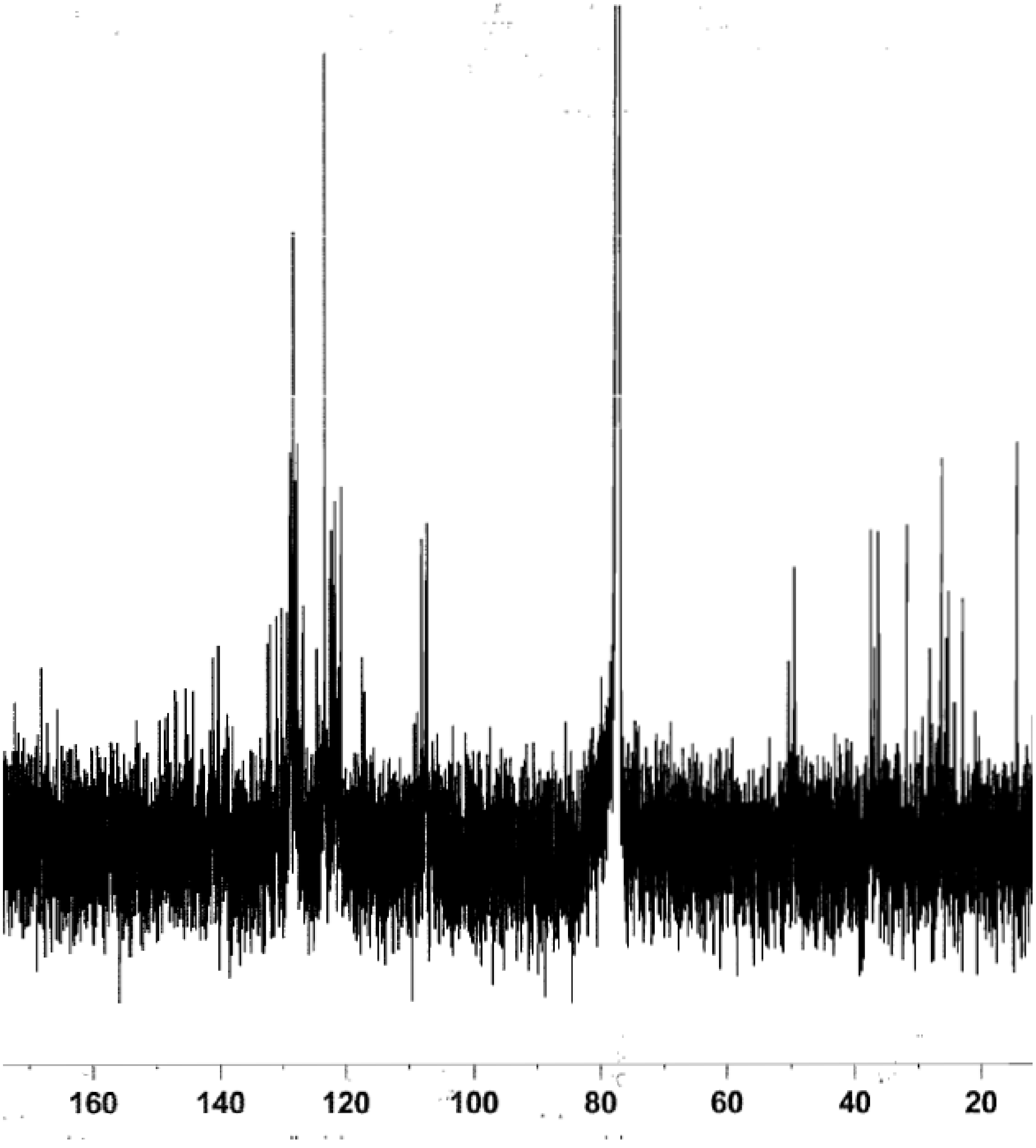

5. ^{1}H and ^{13}C NMR Spectra of the Compounds 10

5.1. (*Z*)-3-((2*Z*,4*E*)-1,3-Diphenyl-4-(1,3,3-trimethylindolin-2-ylidene)but-2-en-1-ylidene)-1-tosylindolin-2-one (10a)

^{1}H NMR (300 MHz, $CDCl_3$, T = 298 K)

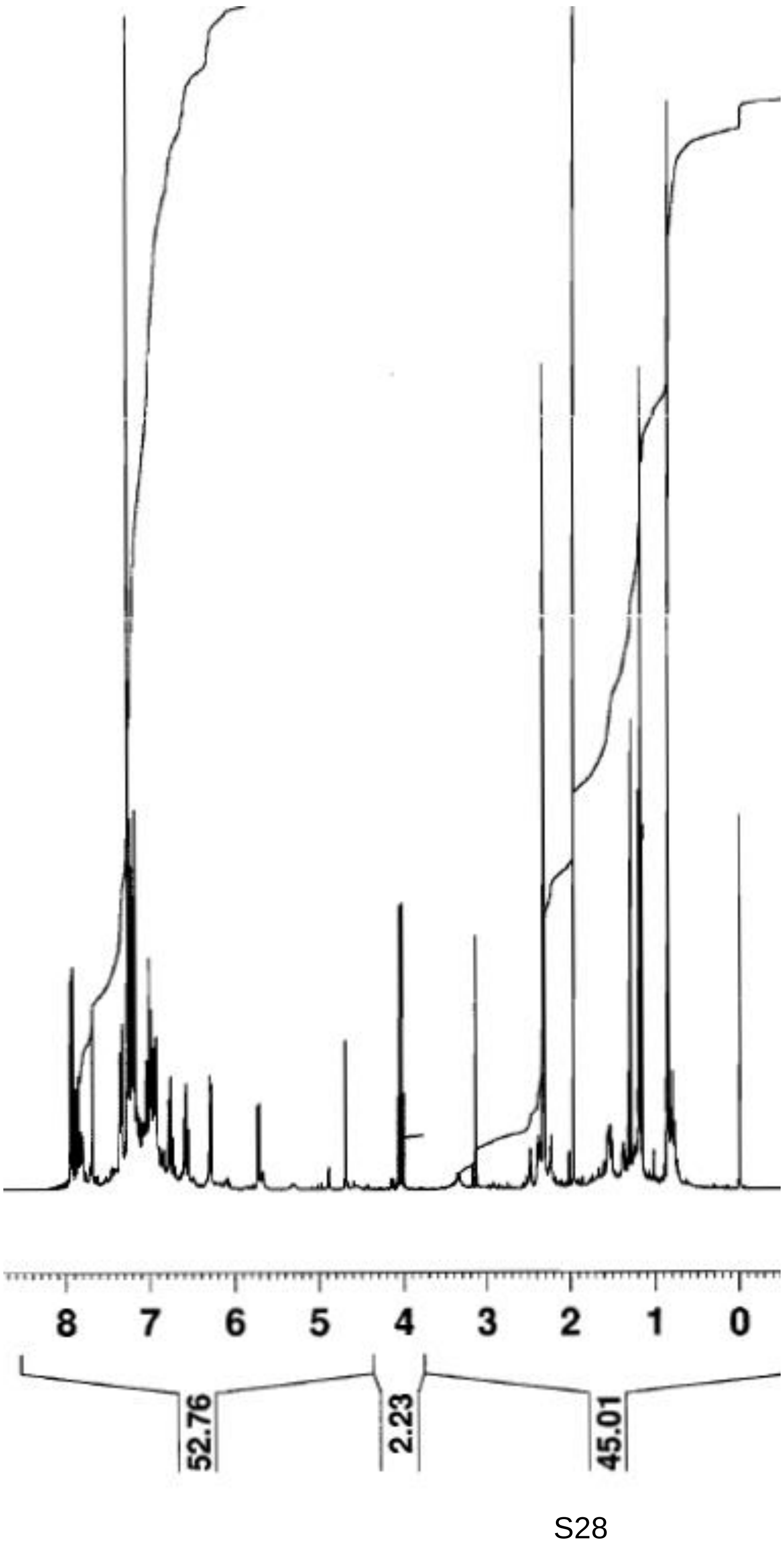

^{13}C NMR (75 MHz, $CDCl_3$, T = 298 K)

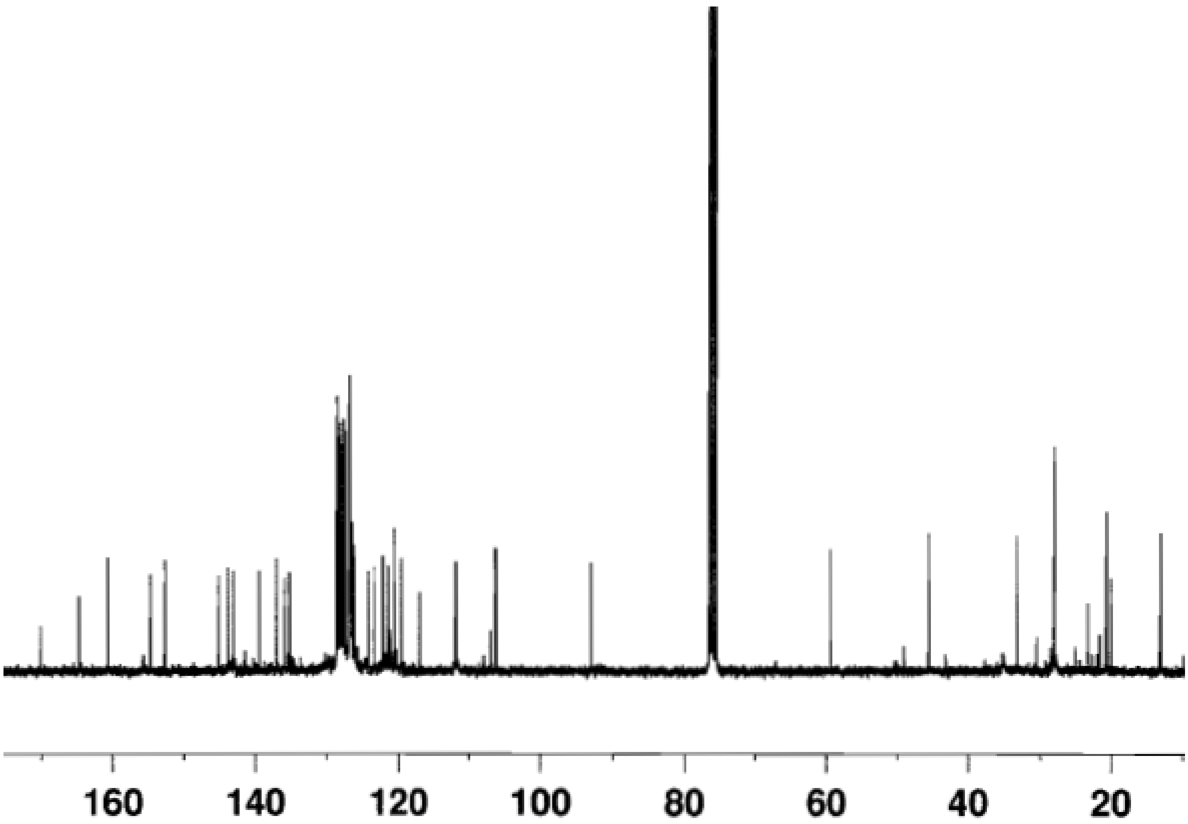

5.2. (*Z*)-3-((2*Z*,4*E*)-3-(4-Aminophenyl)-1-phenyl-4-(1,3,3-trimethylindolin-2-ylidene)but-2-en-1-ylidene)-1-tosylindolin-2-one (10b)

^{1}H NMR (500 MHz, $CDCl_3$, T = 298 K)

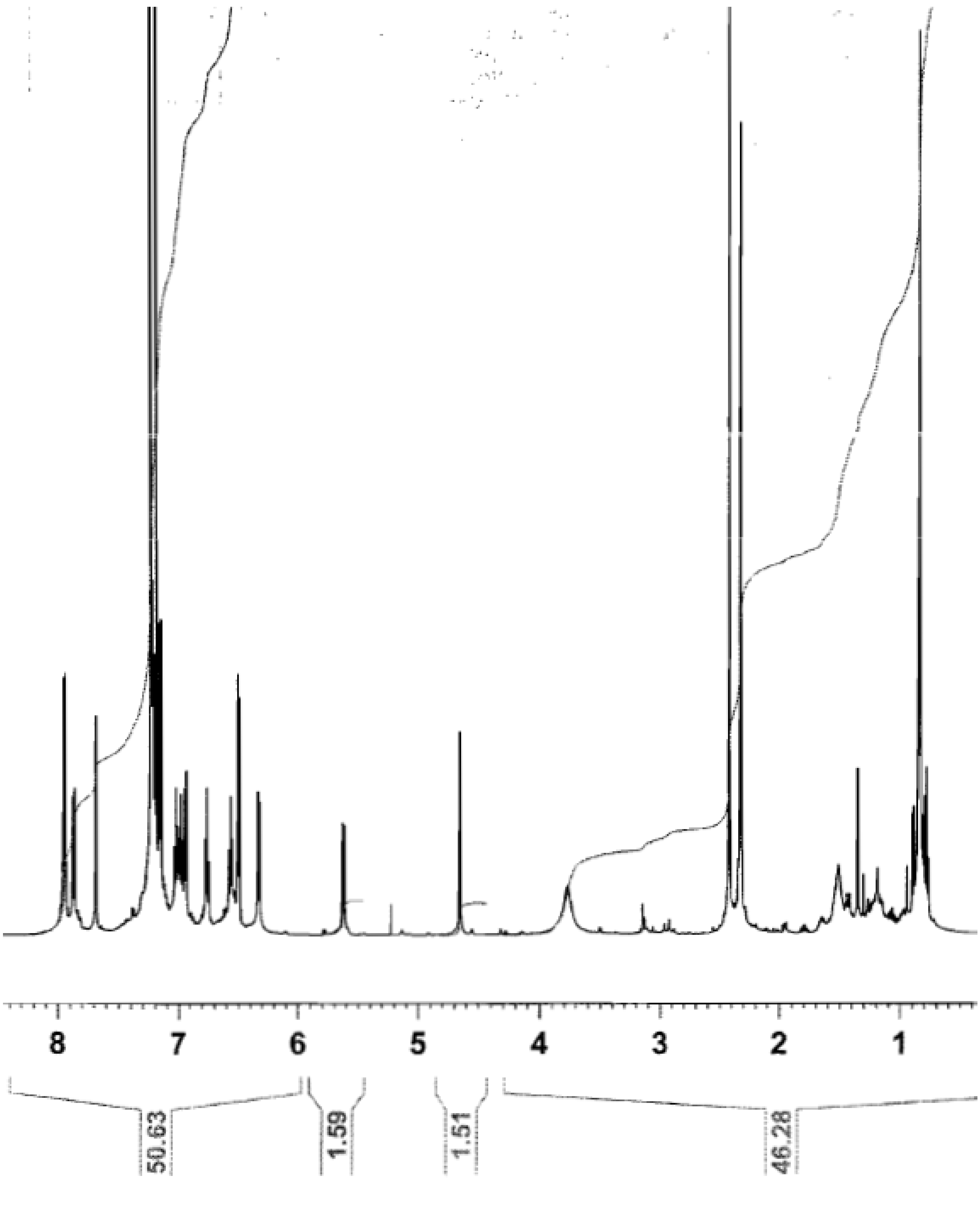

^{13}C NMR (125 MHz, $CDCl_3$, T = 298 K)

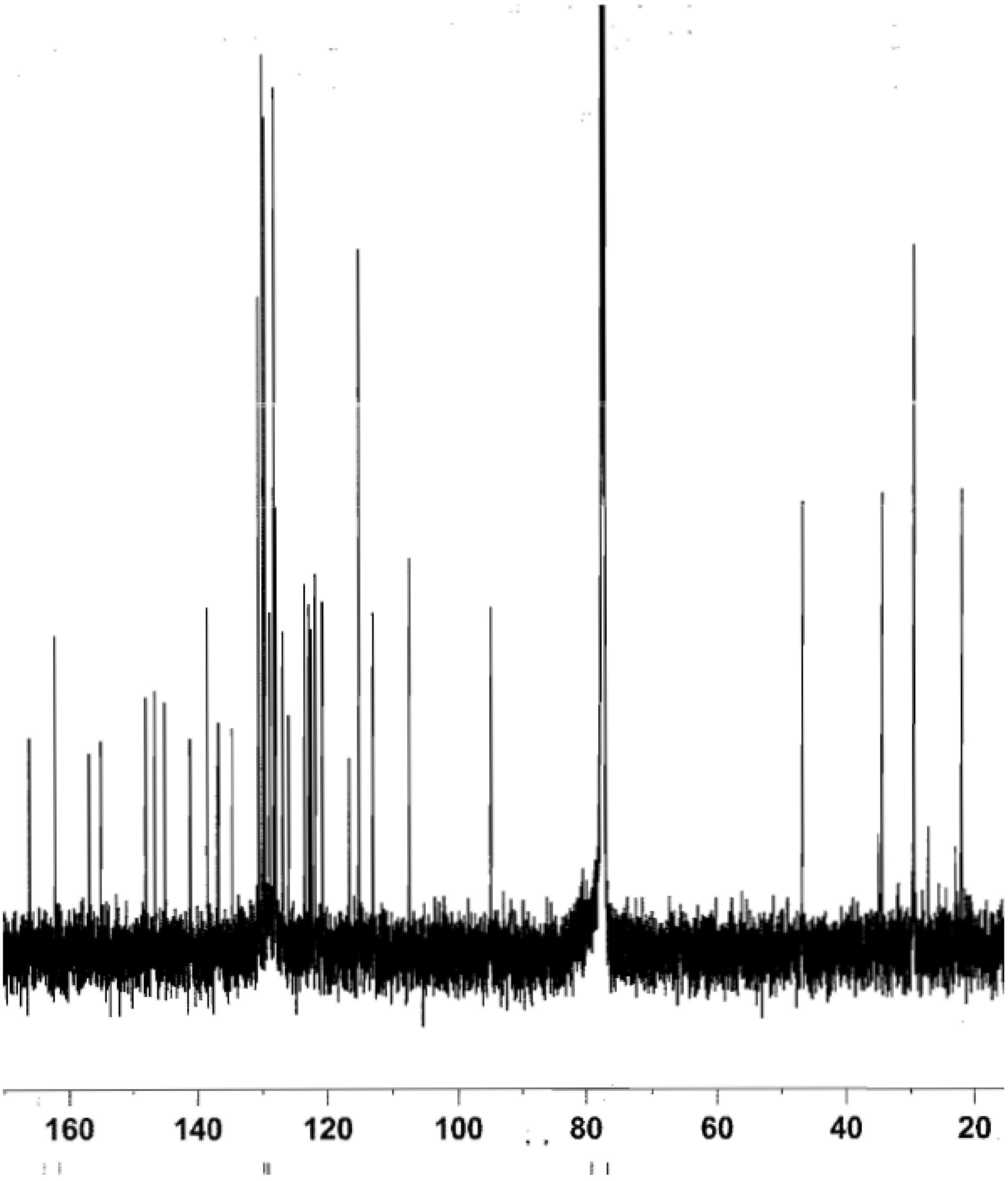

5.3. (*Z*)-3-((2*Z*,4*E*)-3-(4-Chlorphenyl)-1-phenyl-4-(1,3,3-trimethylindolin-2-yliden)but-2-en-1-yliden)-1-tosylindolin-2-on (10c)

^{1}H NMR (500 MHz, $CDCl_3$, T = 298 K)

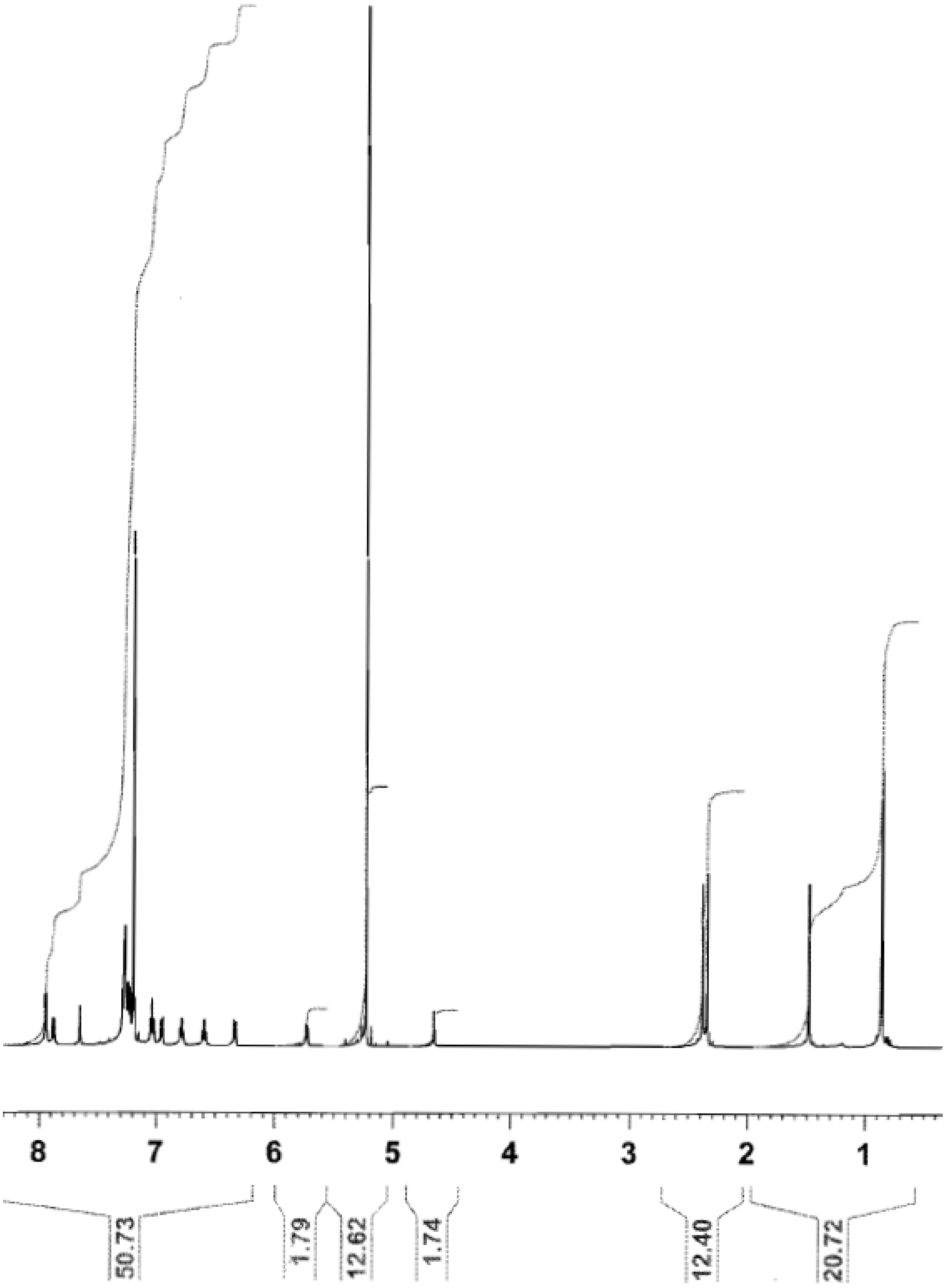

^{13}C NMR (75 MHz, $CDCl_3$, T = 298 K)

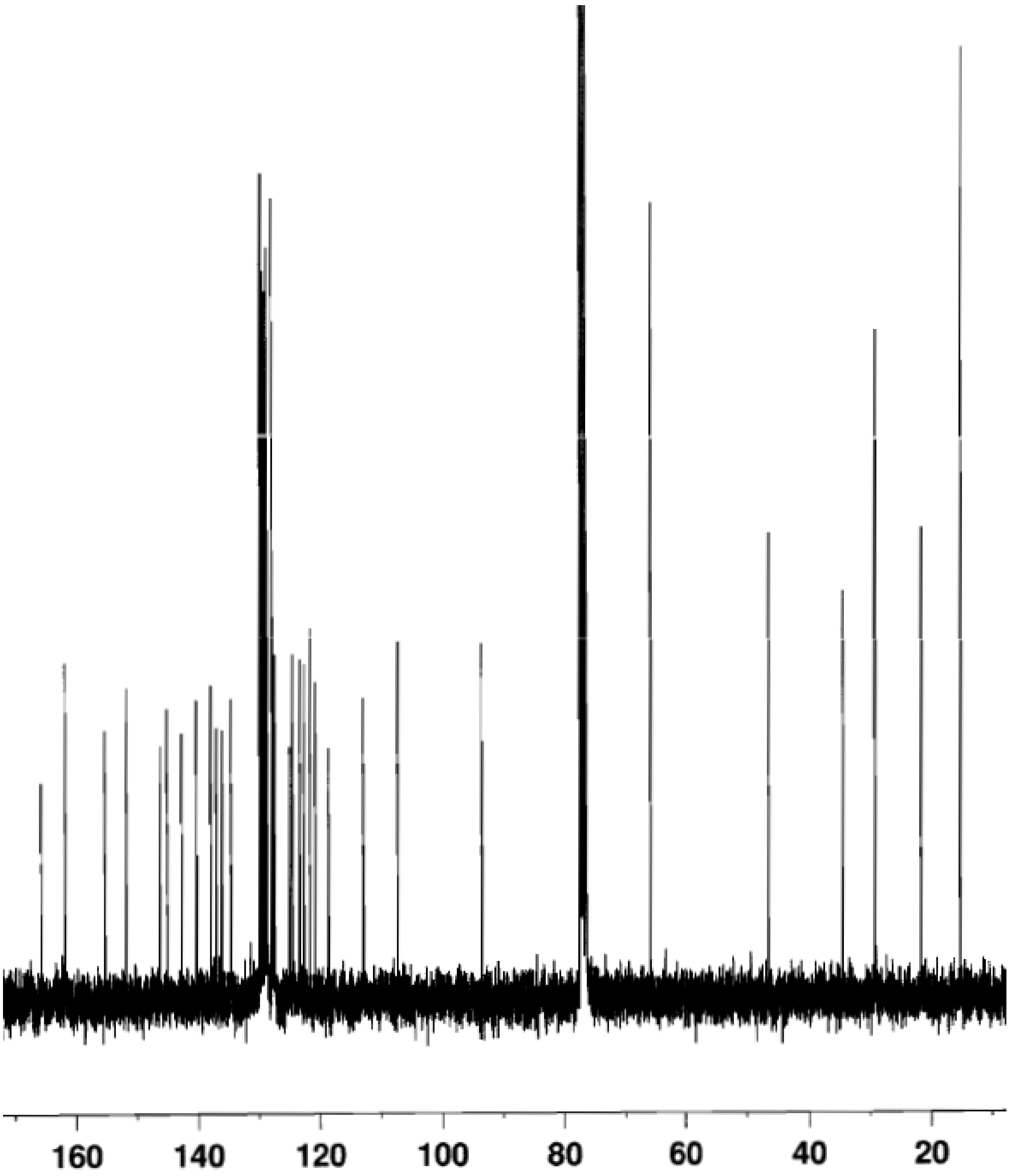

5.4. 4-((1*Z*,2*Z*,4*E*)-4-(2-Oxo-1-tosylindolin-3-ylidene)-4-phenyl-1-(1,3,3-trimethyl-indolin-2-ylidene)but-2-en-2-yl)benzonitrile (10d)

^{1}H NMR (300 MHz, $CDCl_3$, T = 298 K)

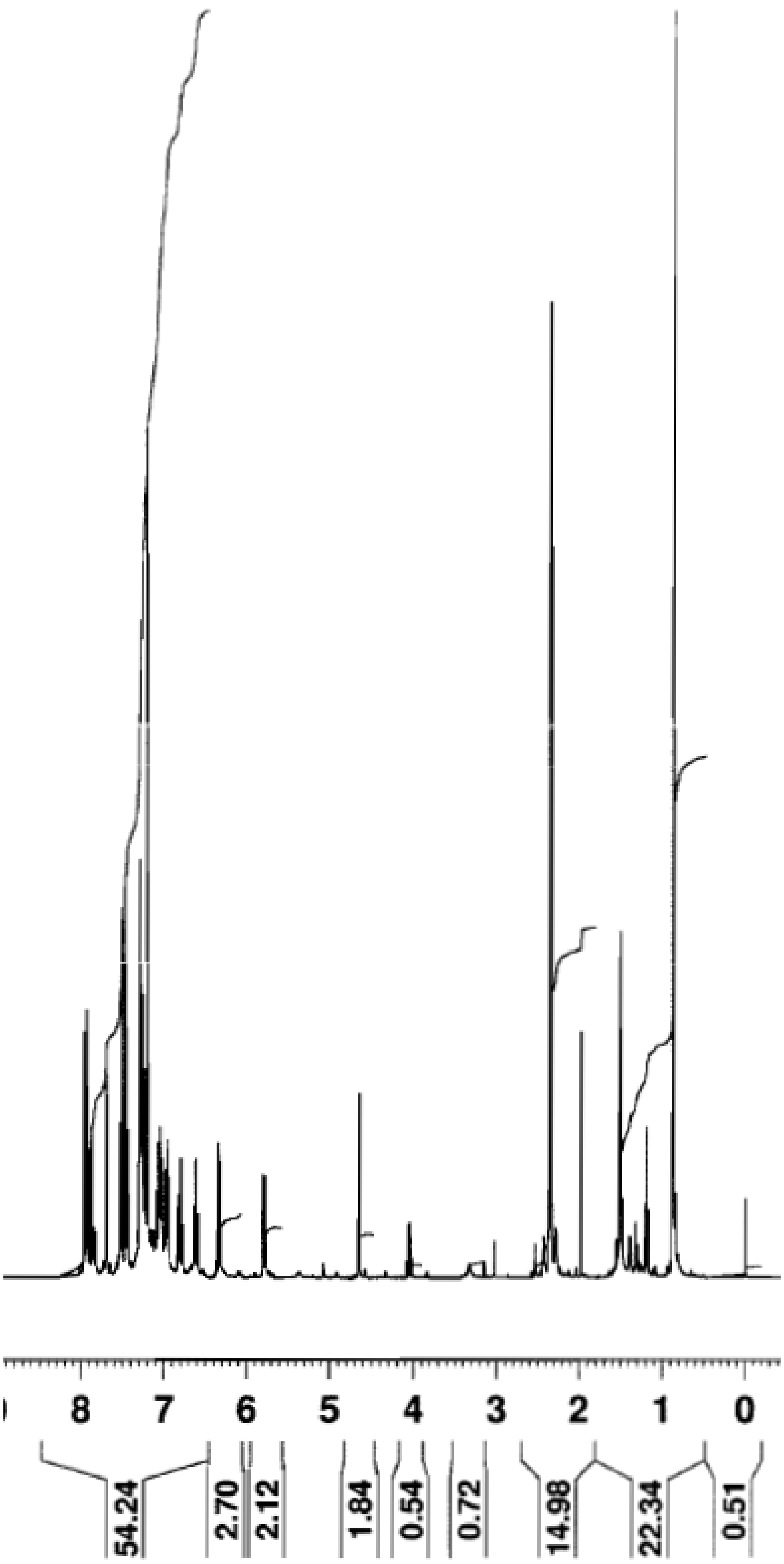

^{13}C NMR (75 MHz, $CDCl_3$, T = 298 K)

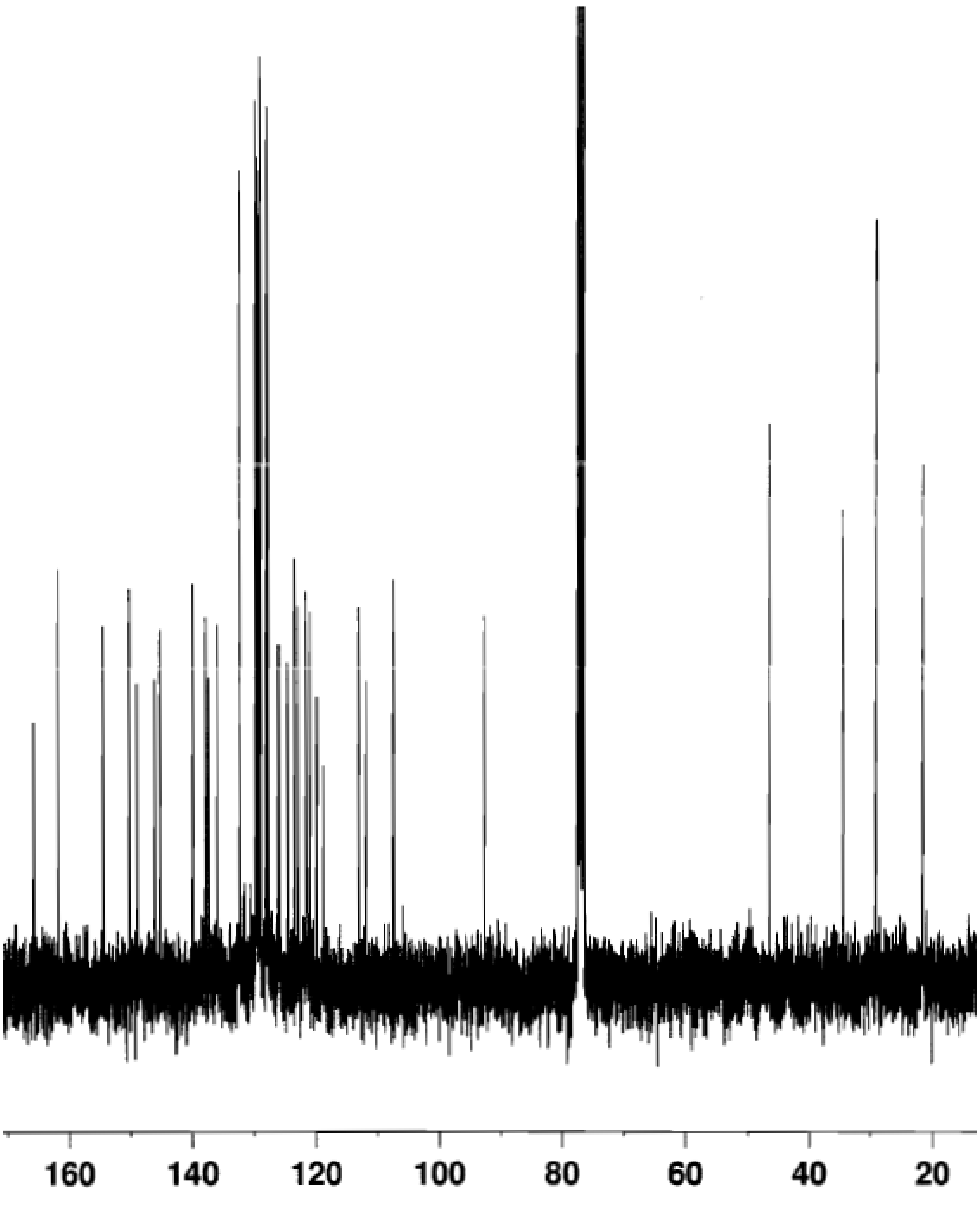

5.5. (*Z*)-3-((2*Z*,4*E*)-3-(4-(tert-Butyl)phenyl)-1-(4-chlorophenyl)-4-(1,3,3-trimethyl-indolin-2-ylidene)but-2-en-1-ylidene)-1-tosylindolin-2-one (10e)

^1H NMR (300 MHz, $CDCl_3$, T = 298 K)

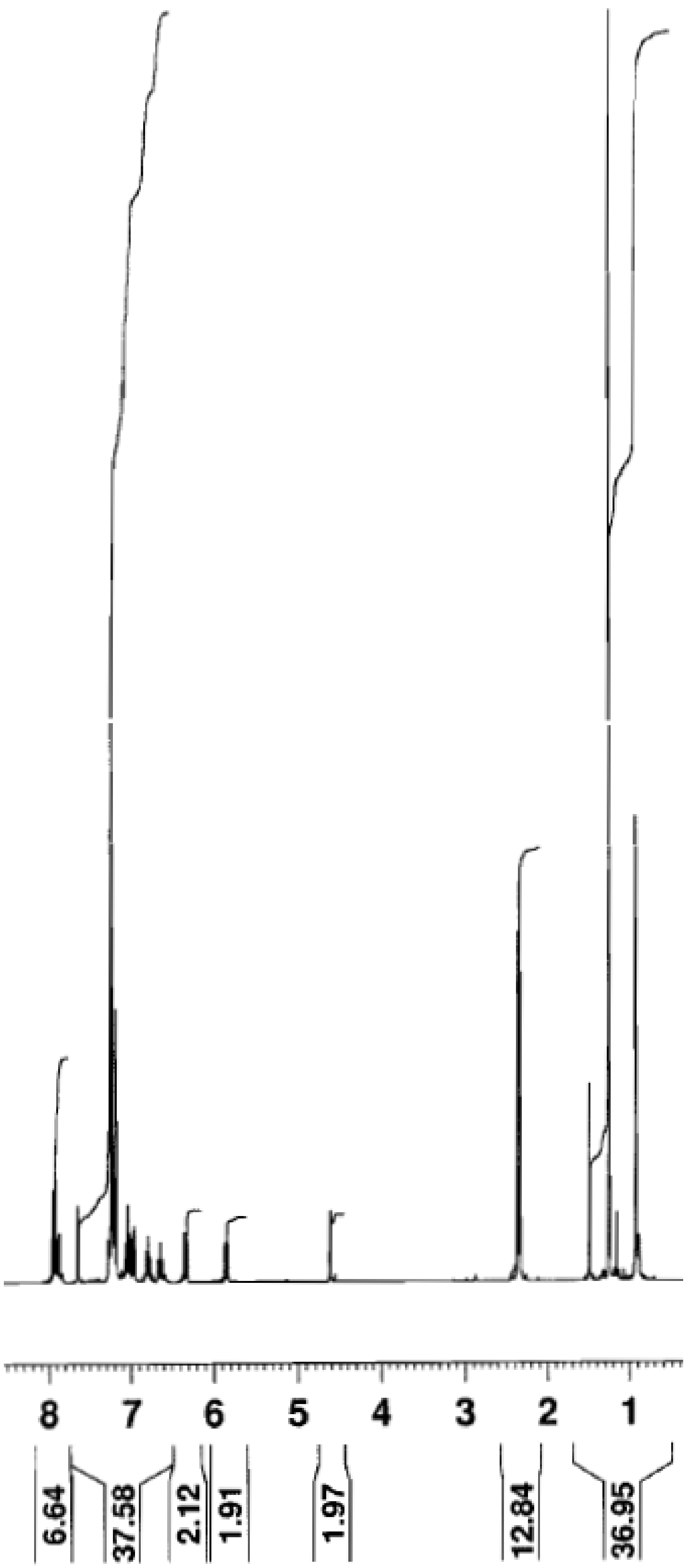

^{13}C NMR (75 MHz, $CDCl_3$, T = 298 K)

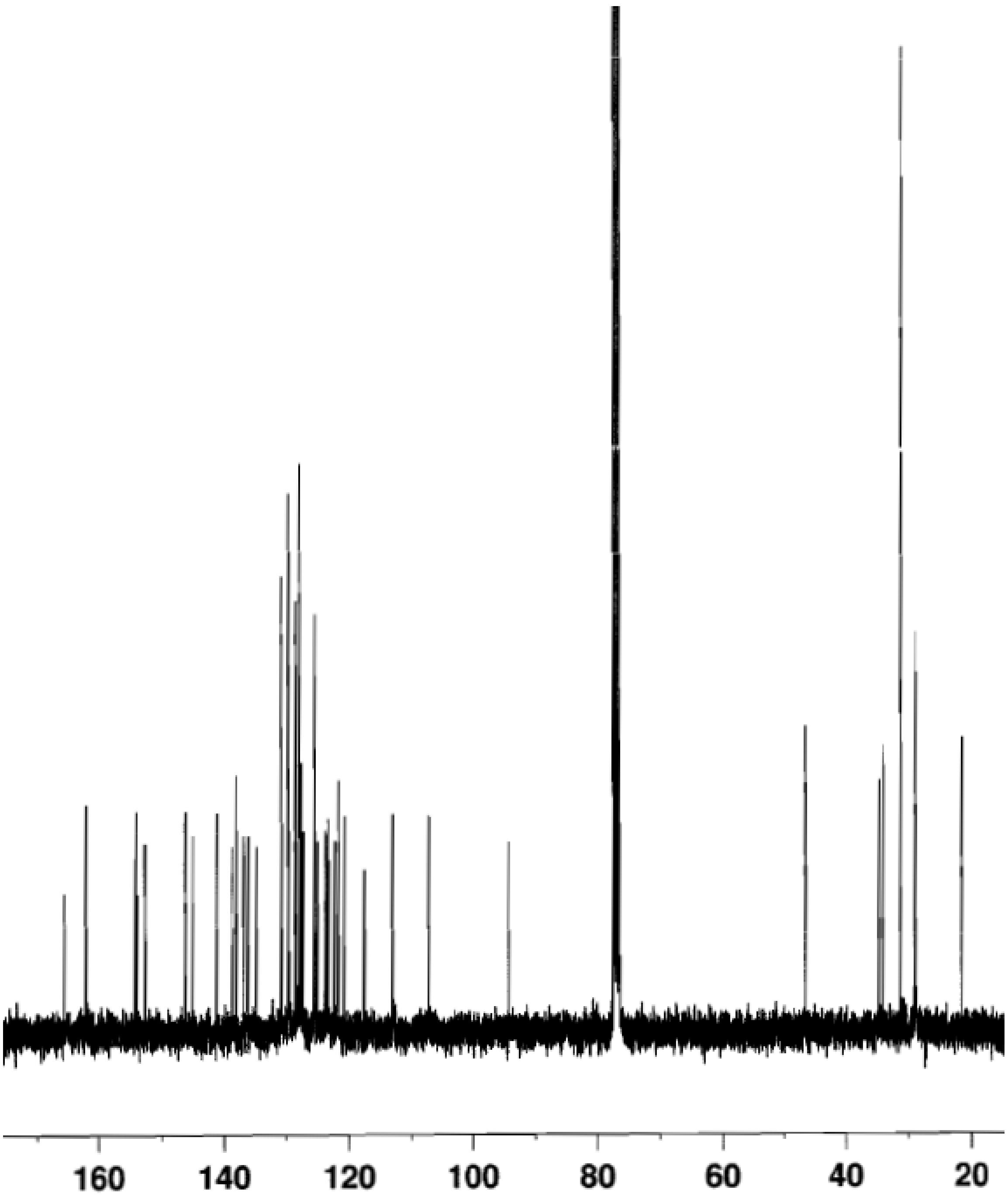

5.6. (*Z*)-3-((2*Z*,4*E*)-1,3-Bis(4-chlorophenyl)-4-(1,3,3-trimethylindolin-2-ylidene)but-2-en-1-ylidene)-1-tosylindolin-2-one (10f)

^{1}H NMR (500 MHz, $CDCl_3$, T = 298 K)

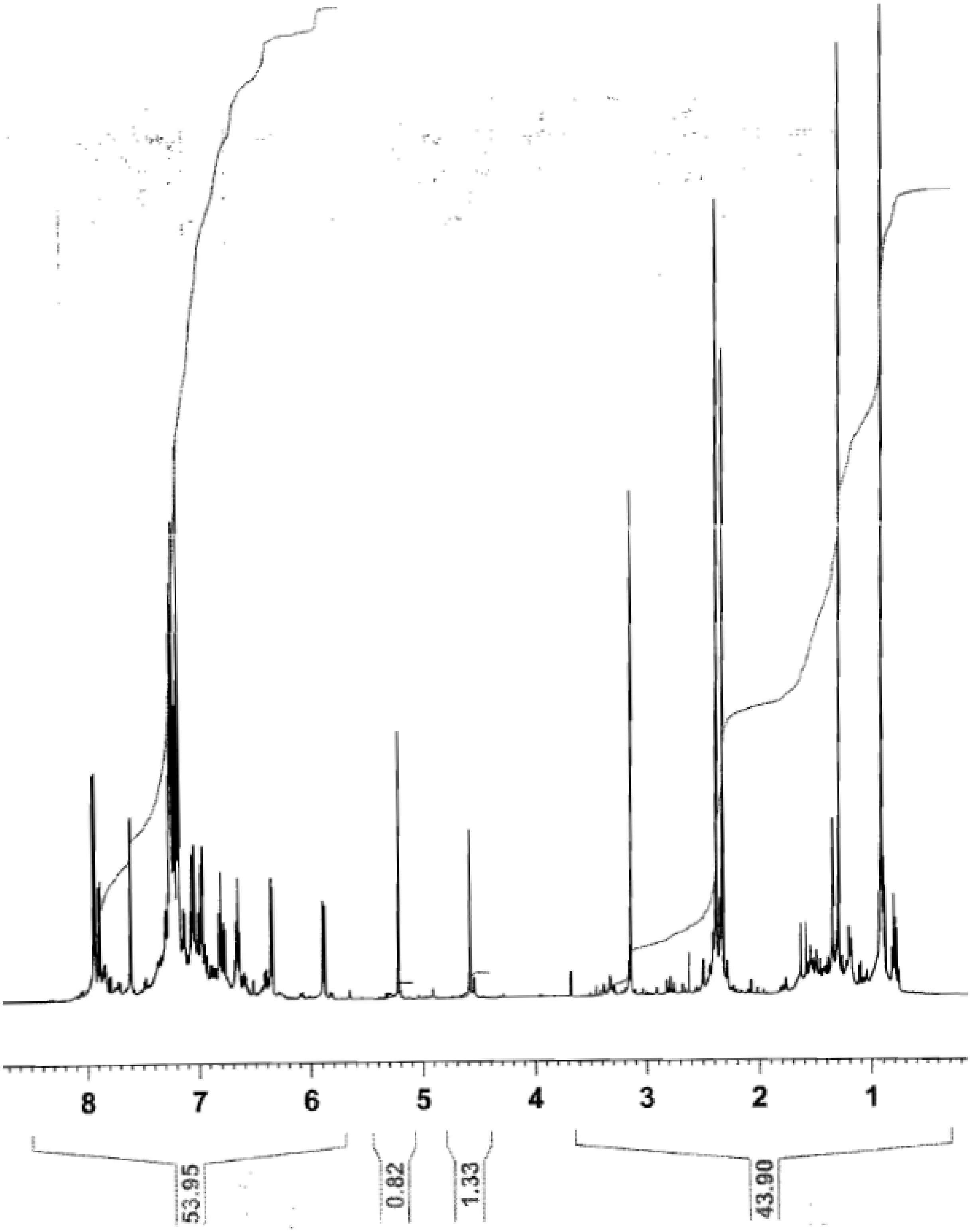

^{13}C NMR (125 MHz, $CDCl_3$, T = 298 K)

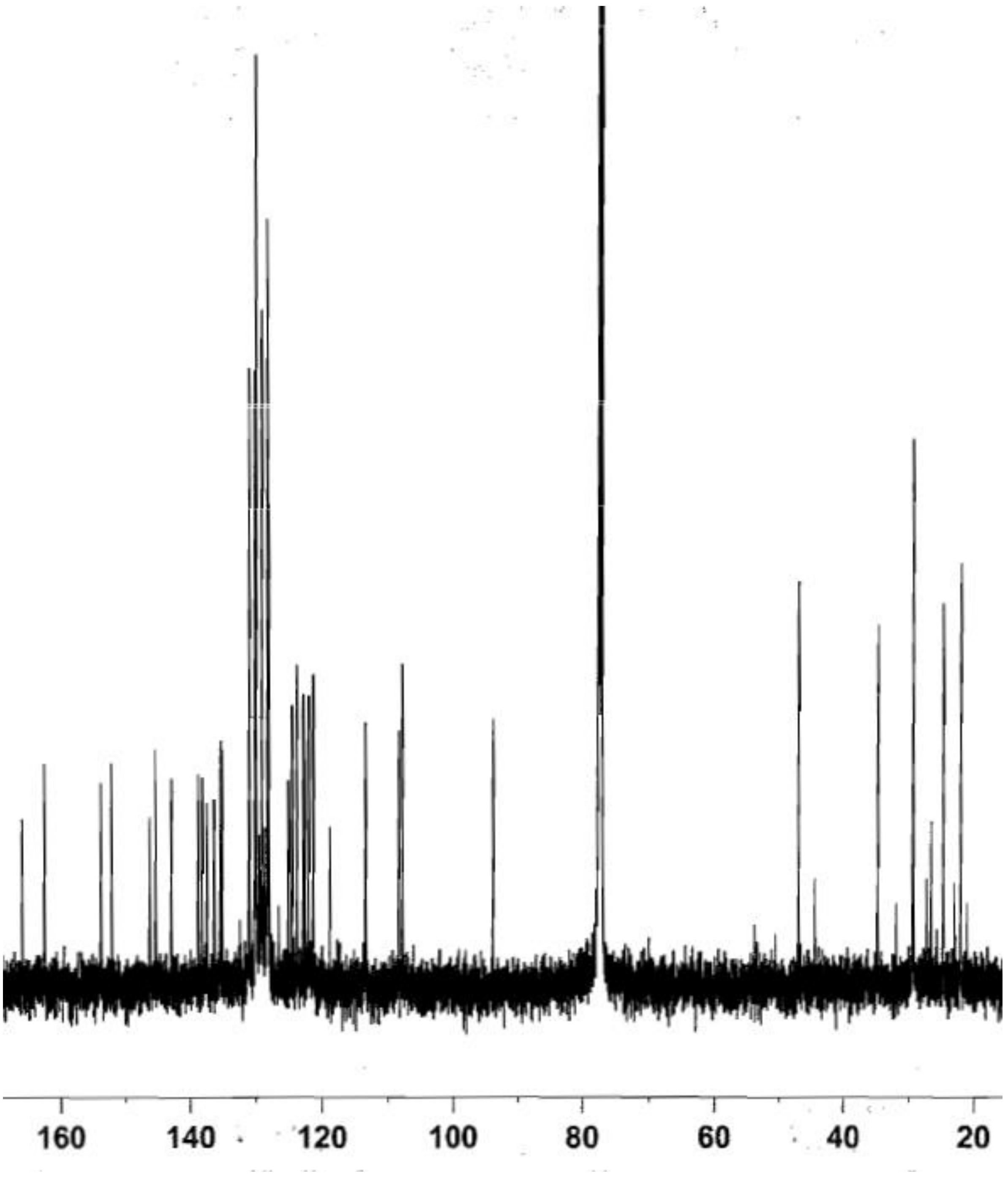

5.7. 4-((1*Z*,2*Z*,4*E*)-4-(4-Chlorophenyl)-4-(2-oxo-1-tosylindolin-3-ylidene)-1-(1,3,3-trimethylindolin-2-ylidene)but-2-en-2-yl)benzonitrile (10g)

^{1}H NMR (300 MHz, $CDCl_3$, T = 298 K)

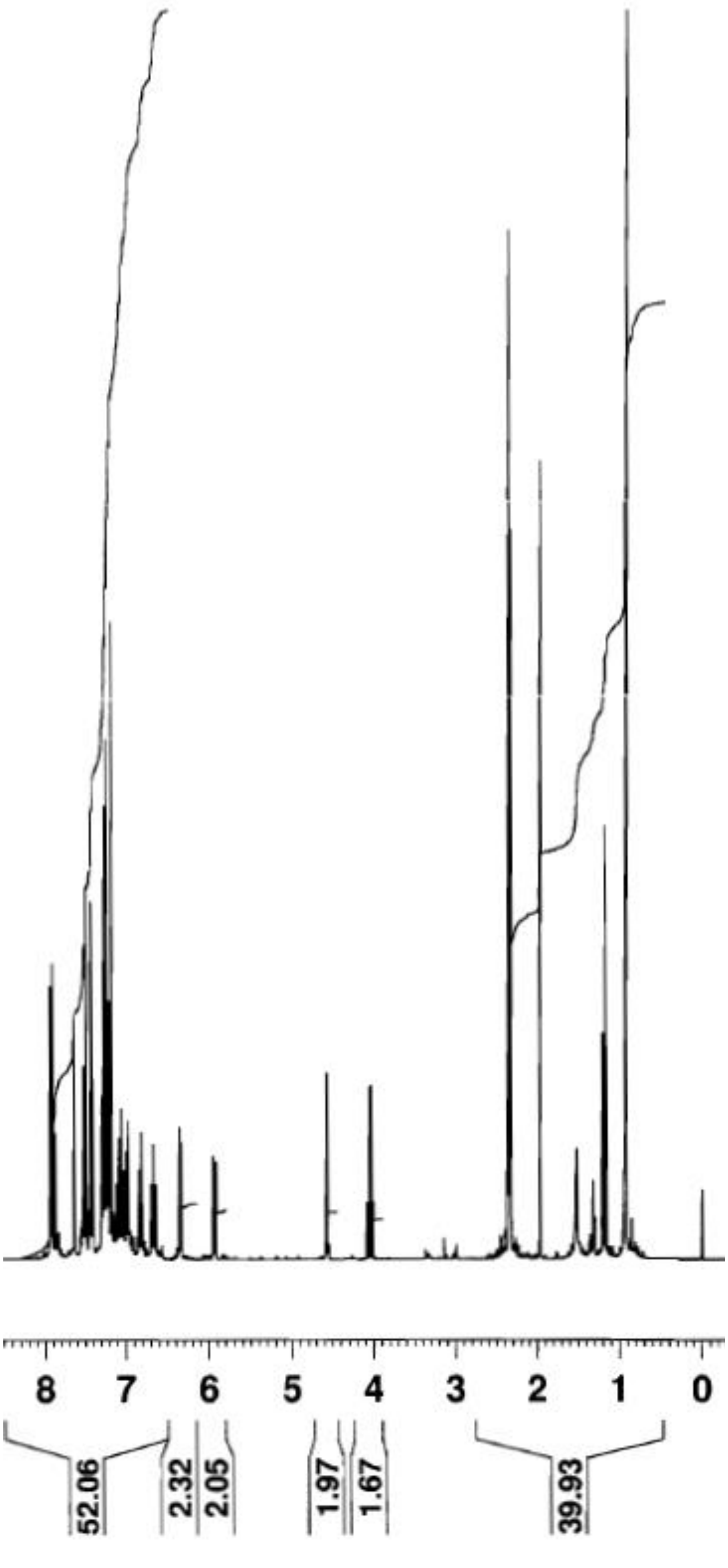

^{13}C NMR (75 MHz, $CDCl_3$, T = 298 K)

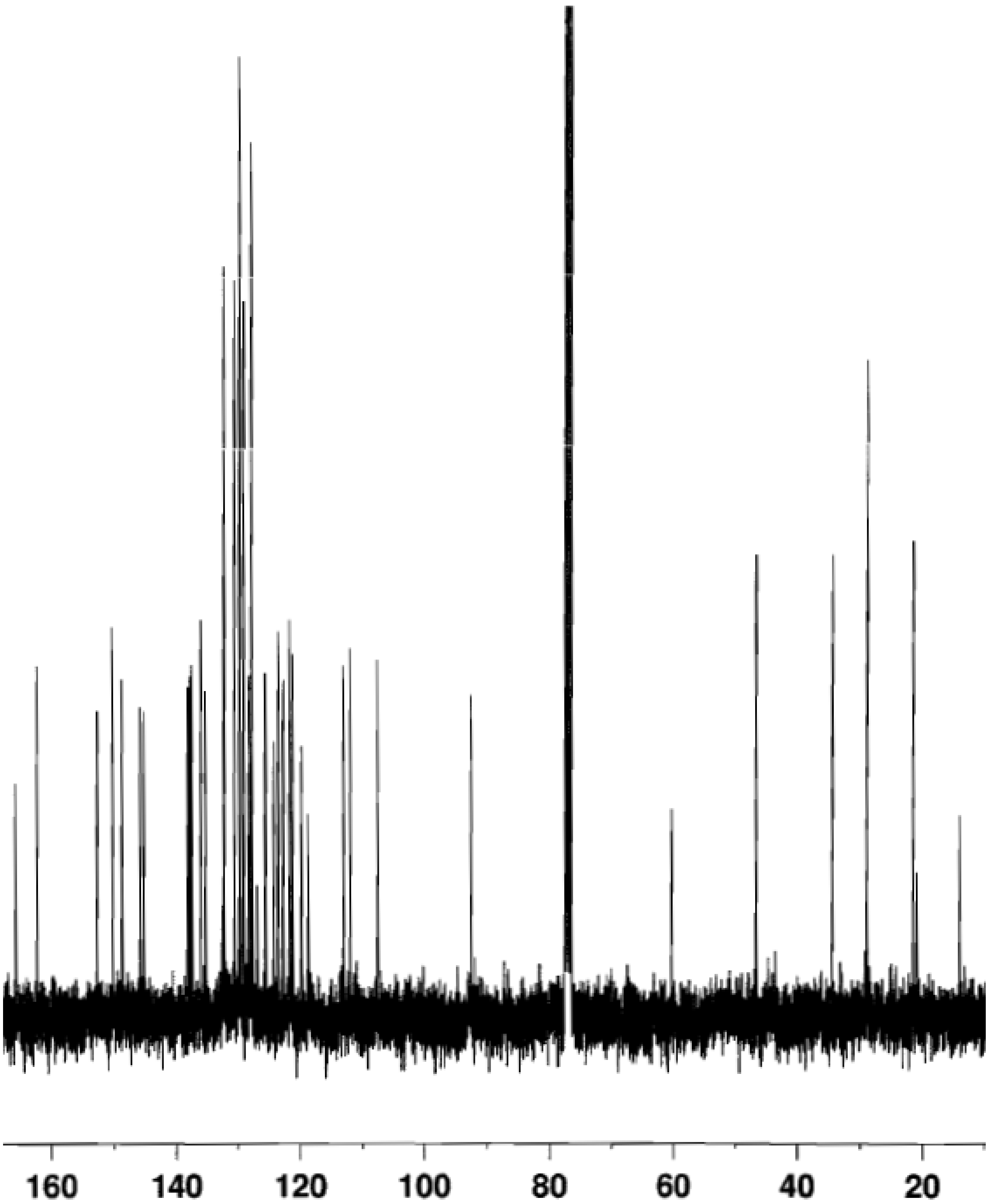

5.8. (*Z*)-3-((2*Z*,4*Z*)-3-(4-Chlorophenyl)-4-(3-methylbenzo[*d*]thiazol-2(3*H*)-ylidene)-1-phenylbut-2-en-1-ylidene)-1-methylindolin-2-one (10h)

^{1}H NMR (300 MHz, $CDCl_3$, T = 298 K)

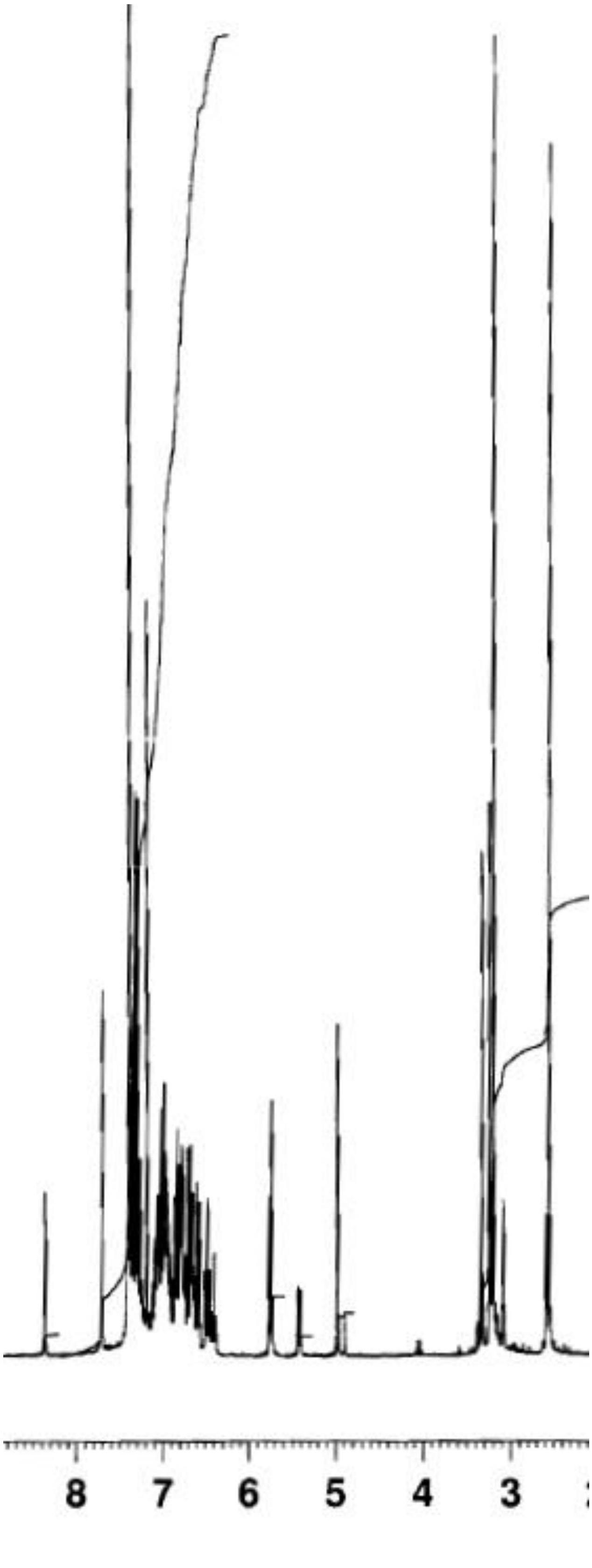

^{13}C NMR (75 MHz, $CDCl_3$, T = 298 K)

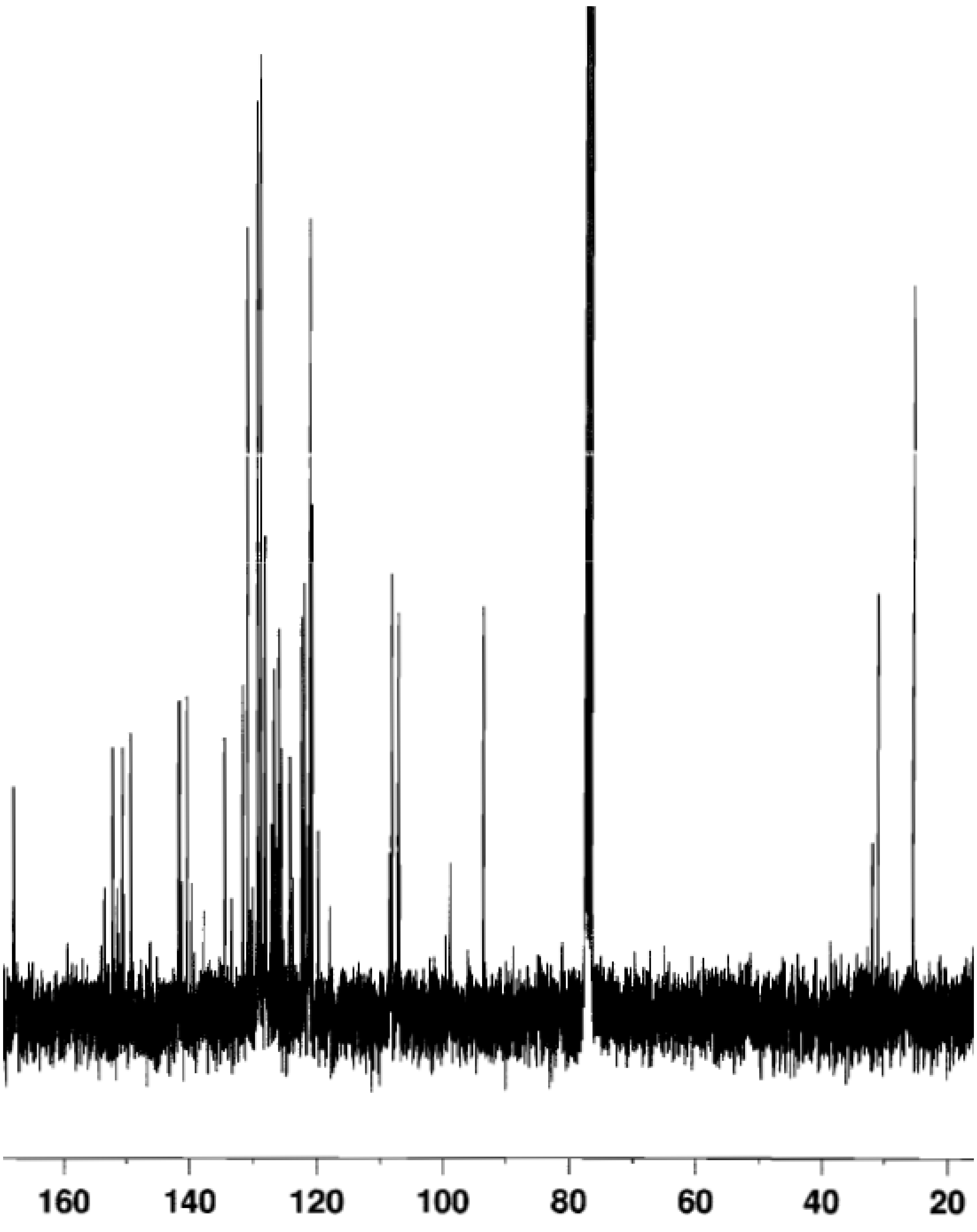

6. Computational data of Structures 2*E*,4*Z*,6*E*-10a, 2*E*,4*Z*,6*Z*-10a, 2*E*,4*Z*,6*Z*-10h, 2*E*,4*Z*,6*E*-10h

Programs used for the computations:

1) DFT-Computations at the B3LYP 6-31G* level of theory: SPARTAN '08, Wavefunction Inc. Irvine CA, **2008**.

Wavefunction Developers: B. J. Deppmeier, A. J. Driessen, T. S. Hehre, W. J. Hehre, J. A. Johnson, P. E. Klunzinger, J. M. Leonard, I. N. Pham, W. J. Pietro, J. Yu

Q-Chem Developers: Y. Shao, L. Fusti-Molnar, Y. Jung, J. Kussmann, C. Ochsenfeld, S. T. Brown, A. T. B. Gilbert, L. V. Slipchenko,S. V. Levchenko, D. P. O'Neill, R. A. Distasio Jr., R. C. Lochan, T. Wang, G. J. O. Beran, N. A. Besley, J. M., Herbert, C. Y. Lin, T. Van Voorhis, S. H. Chien, A. Sodt, R. P. Steele, V. A. Rassolov, P. E. Maslen, P. P. Korambath, R. D. Adamson, B. Austin, J. Baker, E. F. C. Byrd, H. Dachsel, R. J. Doerksen, A. Dreuw, B. D. Dunietz, A. D. Dutoi, T. R. Furlani, S. R. Gwaltney, A. Heyden, S. Hirata, C.-P. Hsu, G. Kedziora, R. Z. Khalliulin, P. Klunzinger, A. M. Lee, M. S. Lee, W. Liang, I. Lotan, N. Nair, B. Peters, E. I. Proynov, P. A. Pieniazek, Y. M. Rhee, J. Ritchie, E. Rosta, C. D. Sherrill, A. C. Simmonett, J. E. Subotnik, H. L. Woodcock III, W. Zhang, A. T. Bell, A. K. Chakraborty, D. M. Chipman, F. J. Keil, A. Warshel, W. J. Hehre, H. F. Schaefer III, J. Kong, A. I. Krylov, P. M. W. Gill, M. Head-Gordon, Phys. Chem. Chem. Phys., (2006) **8**, 3172 - 3191.

2) TD-DFT-computations at the B3LYP 6-311G(d,p) level of theory:

M. J. Frisch, G. W. Trucks, H. B. Schlegel, G. E. Scuseria, M. A. Robb, J. R. Cheeseman, G. Scalmani, V. Barone, B. Mennucci, G. A. Petersson, H. Nakatsuji, M. Caricato, X. Li, H. P. Hratchian, A. F. Izmaylov, J. Bloino, G. Zheng, J. L. Sonnenberg, M. Hada, M. Ehara, K. Toyota, R. Fukuda, J. Hasegawa, M. Ishida, T. Nakajima, Y. Honda, O. Kitao, H. Nakai, T. Vreven, J. A. Montgomery, Jr., J. E. Peralta, F. Ogliaro, M. Bearpark, J. J. Heyd, E. Brothers, K. N. Kudin, V. N. Staroverov, R. Kobayashi, J. Normand, K. Raghavachari, A. Rendell, J. C. Burant, S. S. Iyengar, J. Tomasi, M. Cossi, N. Rega, J. M. Millam, M. Klene, J. E. Knox, J. B. Cross, V. Bakken, C. Adamo, J. Jaramillo, R. Gomperts, R. E. Stratmann, O. Yazyev, A. J. Austin, R. Cammi, C. Pomelli, J. W. Ochterski, R. L. Martin, K. Morokuma, V. G. Zakrzewski, G. A. Voth, P. Salvador, J. J. Dannenberg, S. Dapprich, A. D. Daniels, O. Farkas, J. B. Foresman, J. V. Ortiz, J. Cioslowski, and D. J. Fox, *GAUSSIAN 09* (*Revision A.02*) Gaussian, Inc., Wallingford CT, **2009**.

6.1. Structure 2*Z*,4*Z*-8a

Cartesian Coordinates (Angstroms)

Atom			X	Y	Z
1	C	C3	-1.2002130	2.7889854	-0.8203241
2	C	C4	-2.7173379	5.0836081	-0.2092585
3	C	C5	-1.8929048	3.4855890	-1.8257576
4	C	C6	-1.2780404	3.2660505	0.4987773
5	C	C7	-2.0252711	4.4047759	0.7963856
6	C	C8	-2.6472088	4.6198589	-1.5239225
7	H	H1	-1.8528651	3.1199287	-2.8484612
8	H	H6	-0.7236759	2.7493956	1.2736502
9	H	H5	-2.0654390	4.7649816	1.8215421
10	H	H7	-3.1856933	5.1356165	-2.3151811
11	C	C11	0.9511170	-1.7494845	-0.7249413
12	C	C12	0.9653708	-4.3887427	-1.7146869
13	C	C13	1.6452169	-2.7710749	-0.0504075
14	C	C14	0.2653371	-2.0813650	-1.9068753
15	C	C15	0.2808781	-3.3848592	-2.4018029
16	C	C16	1.6462312	-4.0768700	-0.5350628
17	H	H9	2.1815987	-2.5311250	0.8626660
18	H	H10	-0.2638916	-1.3023888	-2.4455053
19	H	H11	-0.2450657	-3.6169053	-3.3241928
20	H	H13	2.1768788	-4.8532826	0.0098275
21	H	H14	0.9680264	-5.4070491	-2.0942771
22	C	C17	3.4993913	-0.0473437	-0.0444348
23	C	C19	6.2937122	0.0613430	-0.2871233
24	C	C20	4.1577305	-0.9109771	-0.9242057
25	C	C21	4.2732819	0.8932328	0.6797093
26	C	C22	5.6580312	0.9576472	0.5797086
27	C	C23	5.5508566	-0.8545900	-1.0367361
28	H	H12	3.5996383	-1.6276081	-1.5160849
29	H	H16	6.2296904	1.6857806	1.1472062
30	H	H19	6.0580879	-1.5336745	-1.7163220
31	H	H20	7.3758786	0.0879490	-0.3817558
32	N	N1	3.4280557	1.7065309	1.4361448
33	C	C43	-2.9860329	-0.1881623	-0.3165209
34	C	C28	-3.5307289	-1.4652842	0.3191573
35	C	C29	-4.1073901	-3.7051418	1.8668944
36	C	C30	-2.5965308	-1.9664222	1.2310249
37	C	C31	-4.7639588	-2.0838080	0.1839128
38	C	C32	-5.0480868	-3.2204099	0.9572438
39	C	C33	-2.8623812	-3.0825761	2.0211219
40	H	H24	-5.5057606	-1.6987080	-0.5121067
41	H	H25	-6.0047307	-3.7230851	0.8471304
42	H	H26	-2.1294108	-3.4729968	2.7202003
43	H	H27	-4.3367981	-4.5847133	2.4626685
44	C	C27	-0.4903261	-1.1737948	2.3003589
45	H	H4	0.0028963	-0.2004799	2.3423069
46	H	H15	0.2643019	-1.9623523	2.2032568
47	H	H22	-1.0420695	-1.3352892	3.2340627
48	C	C26	-3.0880428	-0.2246907	-1.8506711
49	H	H23	-2.6401214	0.6646027	-2.2997992
50	H	H28	-4.1405279	-0.2618067	-2.1539679
51	H	H29	-2.5875279	-1.1090049	-2.2550698
52	C	C44	-3.8162408	0.9945497	0.2517763
53	H	H30	-4.8774102	0.8114538	0.0473623

54	H	H31	-3.5362568	1.9442736	-0.2027065
55	H	H33	-3.6917466	1.0751693	1.3363441
56	H	H8	-3.3067635	5.9652491	0.0293588
57	C	C2	-0.3699647	0.3720960	-0.3095539
58	C	C34	3.8464365	2.7360429	2.3603523
59	H	H2	4.4119372	3.5213189	1.8441799
60	H	H3	4.4717034	2.3180794	3.1590255
61	H	H21	2.9428805	3.1659069	2.7957138
62	N	N2	-1.4407394	-1.1767136	1.1977178
63	C	C1	-1.5128980	-0.2255295	0.1817195
64	C	C9	0.9284323	-0.3556348	-0.1962428
65	C	C24	-0.3612069	1.6009951	-1.1643933
66	C	C10	2.0888800	0.2346614	0.2501182
67	C	C18	2.0960676	1.3715696	1.2182026
68	O	O1	1.1589434	1.8794680	1.8341902
69	C	C25	0.4383653	1.6743742	-2.2497416
70	H	H17	0.5106022	2.5875431	-2.8320401
71	H	H35	1.0707511	0.8480412	-2.5580770

Convergence after 18 cycles

Heat of Formation: -1575.813533 a.u.

Solvation Energy SM5.4/P: -1575.81585 a.u.

6.2. Structure 2*Z*,4*E*-8a

Cartesian Coordinates (Angstroms)

Atom		X	Y	Z
---------		-------------	-------------	-------------
1 C	C3	-1.4805687	2.1581656	-1.2721120
2 C	C4	-3.5050045	4.0978752	-0.9352383
3 C	C5	-2.0292109	2.8366906	-2.3759398
4 C	C6	-1.9810929	2.4635949	0.0034843
5 C	C7	-2.9786130	3.4237593	0.1681105
6 C	C8	-3.0240796	3.7995289	-2.2115471
7 H	H1	-1.6846274	2.5885411	-3.3761908
8 H	H6	-1.5525648	1.9689164	0.8650405
9 H	H5	-3.3422267	3.6482896	1.1676842
10 H	H7	-3.4305249	4.3076220	-3.0827400
11 C	C11	1.5404400	-1.8349808	-0.7576890
12 C	C12	2.2198963	-4.4328032	-1.6164874
13 C	C13	2.3490862	-2.6710181	0.0358727
14 C	C14	1.0704465	-2.3369357	-1.9845530
15 C	C15	1.4181876	-3.6163210	-2.4157423
16 C	C16	2.6800249	-3.9567395	-0.3853354
17 H	H9	2.7050821	-2.3066473	0.9946959
18 H	H10	0.4386743	-1.7089950	-2.6048708
19 H	H11	1.0572805	-3.9782641	-3.3749202
20 H	H13	3.2931207	-4.5904890	0.2500032
21 H	H14	2.4796544	-5.4351384	-1.9463156
22 C	C17	3.5616601	0.3701024	0.1614474
23 C	C19	6.2852005	1.0509358	0.2648966
24 C	C20	4.5014034	-0.3914689	-0.5393045
25 C	C21	4.0201330	1.5023889	0.8800499
26 C	C22	5.3643147	1.8497678	0.9518074
27 C	C23	5.8566234	-0.0501598	-0.4804277
28 H	H12	4.1914762	-1.2486676	-1.1249933
29 H	H16	5.6893377	2.7201079	1.5137667
30 H	H19	6.5812730	-0.6518410	-1.0217910
31 H	H20	7.3423096	1.2989460	0.3072252
32 N	N1	2.9331612	2.1767685	1.4326915
33 C	C43	-1.4615138	-1.6559145	1.1184313
34 C	C28	-2.9584597	-1.5425116	1.4039202
35 C	C29	-5.7310558	-1.3188951	1.2985410
36 C	C30	-3.5916190	-0.9806995	0.2905332
37 C	C31	-3.7158375	-1.9978765	2.4709094
38 C	C32	-5.1137607	-1.8708602	2.4214080
39 C	C33	-4.9769904	-0.8665169	0.2074872
40 H	H24	-3.2406995	-2.4484484	3.3391366
41 H	H25	-5.7155445	-2.2065749	3.2610950
42 H	H26	-5.4595315	-0.4271340	-0.6599584
43 H	H27	-6.8137163	-1.2293789	1.2675681
44 C	C27	-2.9927475	-0.3978319	-2.0443303
45 H	H4	-2.0888057	-0.3523738	-2.6502767
46 H	H15	-3.5533907	0.5303120	-2.1893379
47 H	H22	-3.6079233	-1.2430864	-2.3812775
48 C	C26	-0.5941909	-1.2100801	2.3053326
49 H	H23	0.4618772	-1.4331040	2.1306163
50 H	H28	-0.8977896	-1.7504048	3.2094065
51 H	H29	-0.6837761	-0.1363913	2.4874460
52 C	C44	-1.1953830	-3.1502745	0.7791430
53 H	H30	-1.4834311	-3.7694020	1.6361968

54	H	H31	-0.1438016	-3.3341768	0.5572444
55	H	H33	-1.7940471	-3.4677248	-0.0810060
56	H	H8	-4.2849255	4.8434345	-0.8030864
57	C	C2	-0.2456329	-0.0390841	-0.5825321
58	C	C34	3.0023131	3.3618967	2.2566415
59	H	H2	3.4780760	4.1887444	1.7153366
60	H	H3	3.5681853	3.1699195	3.1764986
61	H	H21	1.9776912	3.6374080	2.5122503
62	N	N2	-2.6294112	-0.5978807	-0.6517280
63	C	C1	-1.3402840	-0.7454853	-0.1337550
64	C	C9	1.1529035	-0.4752755	-0.2972778
65	C	C24	-0.3725557	1.1685554	-1.4688684
66	C	C10	2.0930617	0.3685568	0.2451566
67	C	C18	1.7341089	1.5666328	1.0728342
68	O	O1	0.6377256	1.9463055	1.4692160
69	C	C25	0.5498477	1.4136377	-2.4248241
70	H	H17	0.5375184	2.3350883	-2.9977280
71	H	H35	1.3717833	0.7334892	-2.6215778

Convergence after 28 cycles

Heat of Formation: -1575.811048 a.u.

Solvation Energy SM5.4/P: -1575.81350 a.u.

6.3. Structure 2*E*,4*Z*,6*E*-10a

Cartesian Coordinates (Angstroms)

Atom	X	Y	Z
1 C C1	-0.4268355	0.5299964	-0.5455501
2 H H2	-0.1269517	-0.4054743	-1.0009095
3 C C2	-1.7218070	0.5395670	-0.0571182
4 C C3	-2.2554780	1.6413415	0.8051860
5 C C4	-3.1890657	3.6654454	2.5219628
6 C C5	-2.7833804	2.8303927	0.2869618
7 C C6	-2.1956479	1.4844385	2.1989813
8 C C7	-2.6537548	2.4892082	3.0510272
9 C C8	-3.2511921	3.8327802	1.1379822
10 H H1	-2.8091417	2.9778663	-0.7876432
11 H H6	-1.7832173	0.5671547	2.6102951
12 H H5	-2.5956472	2.3513073	4.1276970
13 H H7	-3.6528787	4.7501166	0.7163355
14 C C9	0.6379956	1.4964899	-0.5008751
15 C C10	1.9460163	1.1111832	-0.7705711
16 C C11	0.3692915	2.9479659	-0.2474029
17 C C12	-0.0277624	5.6958944	0.1667861
18 C C13	0.6172718	3.5201820	1.0068307
19 C C14	-0.0722758	3.7688605	-1.2945308
20 C C15	-0.2646574	5.1353791	-1.0899048
21 C C16	0.4097938	4.8838010	1.2153535
22 H H9	0.9749162	2.8923019	1.8176967
23 H H10	-0.2500080	3.3324976	-2.2738689
24 H H11	-0.5969477	5.7628295	-1.9130884
25 H H13	0.5974900	5.3128468	2.1959564
26 H H14	-0.1791355	6.7601554	0.3269082
27 C C18	2.3670192	-0.3075483	-0.9293645
28 C C17	3.1586434	1.9168852	-0.9466573
29 C C19	5.7423967	2.8977502	-1.4359023
30 C C20	3.4045173	3.2975733	-0.9306903
31 C C21	4.2465000	1.0479288	-1.2180131
32 C C22	5.5312283	1.5160719	-1.4696396
33 C C23	4.6923337	3.7777374	-1.1709415
34 H H12	2.6061382	3.9990749	-0.7332420
35 H H16	6.3373843	0.8320130	-1.6923605
36 H H19	4.8719554	4.8491424	-1.1543813
37 H H20	6.7404503	3.2824785	-1.6269720
38 N N1	3.7590702	-0.2871968	-1.2063358
39 O O1	1.7298897	-1.3472065	-0.8200217
40 C C24	-2.4521633	-0.6967598	-0.2605338
41 H H3	-1.7826880	-1.4836373	-0.5958647
42 C C25	-3.7613492	-1.1084300	-0.1699400
43 C C43	-5.1107497	-0.3618537	-0.0313883
44 N N2	-4.0530851	-2.4613989	-0.3488637
45 C C28	-6.1120653	-1.4891062	-0.2488858
46 C C29	-7.5086113	-3.8524614	-0.6866371
47 C C30	-5.4298203	-2.6953940	-0.4359783
48 C C31	-7.4980070	-1.4647890	-0.2691143
49 C C32	-8.2021218	-2.6574264	-0.4907649
50 C C33	-6.1095396	-3.8916077	-0.6645607
51 H H24	-8.0388877	-0.5332818	-0.1192964
52 H H25	-9.2878684	-2.6487887	-0.5146968
53 H H26	-5.5873841	-4.8275611	-0.8335771

54 H	H27	-8.0586283	-4.7725575	-0.8651436
55 C	C27	-3.0535868	-3.5033326	-0.4679990
56 H	H4	-2.2892385	-3.3790728	0.3056561
57 H	H15	-2.5605204	-3.4905222	-1.4491621
58 H	H22	-3.5290913	-4.4749648	-0.3279300
59 S	S1	4.6455227	-1.7574866	-1.4059410
60 C	C36	4.5200348	-2.5490018	0.1923443
61 C	C37	4.3767084	-3.8045180	2.6821321
62 C	C38	3.5001538	-3.4700392	0.4336889
63 C	C39	5.4731834	-2.2461056	1.1682470
64 C	C40	5.3901007	-2.8738563	2.4077360
65 C	C41	3.4395204	-4.0900858	1.6800960
66 H	H32	2.7704192	-3.6786857	-0.3383735
67 H	H35	6.2688568	-1.5415862	0.9512104
68 H	H36	6.1268811	-2.6434846	3.1733194
69 H	H37	2.6481303	-4.8084267	1.8787821
70 C	C42	4.3194309	-4.5062969	4.0180460
71 H	H38	4.9864610	-5.3784735	4.0307822
72 H	H39	3.3090811	-4.8638765	4.2406301
73 H	H40	4.6349892	-3.8454934	4.8324356
74 O	O2	3.9530885	-2.5637171	-2.4018036
75 O	O3	6.0372477	-1.3497099	-1.5994328
76 C	C26	-5.2673767	0.7000814	-1.1426657
77 H	H23	-4.5443678	1.5082655	-1.0202514
78 H	H28	-6.2728197	1.1344365	-1.1017296
79 H	H29	-5.1294624	0.2545252	-2.1329119
80 C	C44	-5.3464431	0.2614586	1.3638598
81 H	H30	-6.3843036	0.6066480	1.4314276
82 H	H31	-4.6921767	1.1123367	1.5476251
83 H	H33	-5.1878384	-0.4790406	2.1546544
84 H	H8	-3.5497650	4.4483417	3.1838887

Convergence after 26 cycles

Heat of Formation: -2355.438705 a.u.

Solvation Energy SM5.4/P: -2355.45511 a.u.

6.4. Structure 2*E*,4*Z*,6*Z*-10a

Cartesian Coordinates (Angstroms)

Atom			X	Y	Z
1	C	C1	-0.2969000	-0.5596789	-0.1265974
2	H	H2	0.0574556	0.4639654	-0.1450964
3	C	C2	-1.6744918	-0.6840883	-0.0940386
4	C	C3	-2.4044050	-1.9511667	0.1776618
5	C	C4	-3.8642082	-4.2983878	0.7209463
6	C	C5	-2.0597943	-2.7783898	1.2589615
7	C	C6	-3.4976033	-2.3226453	-0.6238697
8	C	C7	-4.2155844	-3.4875729	-0.3608966
9	C	C8	-2.7842971	-3.9381136	1.5297619
10	H	H1	-1.2333112	-2.4920240	1.9025800
11	H	H6	-3.7771643	-1.6906914	-1.4615292
12	H	H5	-5.0510742	-3.7620221	-0.9997912
13	H	H7	-2.5063753	-4.5604809	2.3763226
14	H	H8	-4.4273814	-5.2036416	0.9322031
15	C	C9	0.7609376	-1.5386343	-0.1725655
16	C	C10	2.0671772	-1.1631528	0.1212278
17	C	C11	0.4905107	-2.9279458	-0.6474895
18	C	C12	0.0160701	-5.5447464	-1.5595308
19	C	C13	-0.0950890	-3.1524167	-1.9013231
20	C	C14	0.8325598	-4.0354074	0.1441668
21	C	C15	0.5921414	-5.3321839	-0.3043399
22	C	C16	-0.3225628	-4.4509908	-2.3576078
23	H	H9	-0.3648400	-2.3024163	-2.5213974
24	H	H10	1.2934242	-3.8709341	1.1140801
25	H	H11	0.8559210	-6.1776811	0.3256160
26	H	H13	-0.7673027	-4.6073838	-3.3369301
27	H	H14	-0.1677227	-6.5561645	-1.9120012
28	C	C18	2.3941742	0.1030341	0.8233118
29	C	C17	3.3470418	-1.8171207	-0.1615789
30	C	C19	6.0415490	-2.5299438	-0.4677712
31	C	C20	3.6932861	-2.9709879	-0.8784883
32	C	C21	4.3922158	-1.0136083	0.3620721
33	C	C22	5.7329380	-1.3603981	0.2332069
34	C	C23	5.0353818	-3.3199954	-1.0274631
35	H	H12	2.9265984	-3.5929465	-1.3209383
36	H	H16	6.5091746	-0.7403118	0.6582403
37	H	H19	5.2946704	-4.2173642	-1.5824371
38	H	H20	7.0835532	-2.8171700	-0.5791825
39	N	N1	3.8077576	0.1274818	0.9757037
40	O	O1	1.6631022	0.9963237	1.2282758
41	C	C24	-2.4292076	0.5202342	-0.4100463
42	H	H3	-1.8734913	1.2261362	-1.0226635
43	C	C25	-3.6952501	0.9558588	-0.1254548
44	N	N2	-4.6218860	0.4876051	0.7900282
45	C	C34	-4.3105921	2.1727861	-0.8576914
46	C	C28	-5.8393001	1.1785000	0.6662710
47	C	C29	-8.0038431	2.8216305	0.1277399
48	C	C30	-5.7221245	2.1876098	-0.2970320
49	C	C31	-7.0245611	0.9675194	1.3678206
50	C	C32	-8.1074129	1.8083906	1.0819518
51	C	C33	-6.8002851	3.0161158	-0.5671401
52	H	H24	-7.1191872	0.1744864	2.1026568
53	H	H25	-9.0441111	1.6618479	1.6130358

54	H	H26	-6.7188936	3.8102926	-1.3056579
55	H	H27	-8.8571848	3.4606306	-0.0797172
56	C	C27	-4.3025818	1.9732432	-2.3871357
57	H	H4	-3.2777798	1.8908216	-2.7643578
58	H	H15	-4.7804431	2.8252352	-2.8842890
59	H	H22	-4.8454001	1.0658761	-2.6709784
60	S	S1	4.5977542	1.4847489	1.6962384
61	C	C36	4.2065999	2.8359291	0.5906444
62	C	C37	3.6373218	4.9602354	-1.1316303
63	C	C38	3.0866173	3.6302295	0.8347537
64	C	C39	5.0517557	3.0845154	-0.4942419
65	C	C40	4.7576142	4.1430141	-1.3483573
66	C	C41	2.8135730	4.6875912	-0.0317692
67	H	H32	2.4422615	3.4105459	1.6767066
68	H	H35	5.9269567	2.4638855	-0.6539989
69	H	H36	5.4110742	4.3406880	-2.1948107
70	H	H37	1.9411795	5.3103854	0.1509881
71	C	C42	3.3444755	6.1201738	-2.0531061
72	H	H38	4.0201865	6.9621728	-1.8539104
73	H	H39	2.3198942	6.4828992	-1.9261006
74	H	H40	3.4790498	5.8406783	-3.1040575
75	O	O2	3.9768126	1.7243377	2.9914958
76	O	O3	6.0303557	1.2077498	1.5849846
77	C	C35	-3.5579664	3.4728259	-0.4865764
78	H	H17	-2.5227379	3.4400285	-0.8437288
79	H	H18	-3.5419771	3.6245714	0.5971142
80	H	H21	-4.0506899	4.3376287	-0.9457408
81	C	C26	-4.3371239	-0.3322440	1.9573076
82	H	H23	-4.6329335	-1.3761276	1.8157476
83	H	H28	-4.8772413	0.0825356	2.8151091
84	H	H29	-3.2679387	-0.2953834	2.1666740

Convergence after 23 cycles

Heat of Formation: -2355.445364 a.u.

Solvation Energy SM5.4/P: -2355.46137 a.u.

6.5. Structure 2*E*,4*Z*,6*Z*-10h

Cartesian Coordinates (Angstroms)

Atom		X	Y	Z
---------		-------------	-------------	-------------
1 C	C1	0.7211120	-1.0899984	-0.3601998
2 H	H2	0.7550255	-2.1726716	-0.4139756
3 C	C2	-0.5423787	-0.5378334	-0.3047850
4 C	C3	-0.8020935	0.8945179	0.0425877
5 C	C4	-1.3241305	3.5425298	0.7672405
6 C	C5	-1.1183079	1.8533128	-0.9295838
7 C	C6	-0.7578880	1.2925513	1.3870224
8 C	C7	-1.0201425	2.6105787	1.7572915
9 C	C8	-1.3729344	3.1776565	-0.5770151
10 H	H1	-1.1575586	1.5632301	-1.9749671
11 H	H6	-0.5152716	0.5592541	2.1507648
12 H	H5	-0.9859641	2.9122724	2.7987244
13 H	H7	-1.6075711	3.9175552	-1.3349180
14 C	C9	2.0208196	-0.4652671	-0.3155752
15 C	C10	3.1511522	-1.2022124	-0.0104821
16 C	C11	2.1819215	0.9690595	-0.7156471
17 C	C12	2.5489733	3.6363238	-1.5194563
18 C	C13	2.4656985	1.9651977	0.2280390
19 C	C14	2.0858116	1.3264354	-2.0683268
20 C	C15	2.2768118	2.6493604	-2.4685288
21 C	C16	2.6388806	3.2909487	-0.1692878
22 H	H9	2.5502605	1.6950397	1.2765228
23 H	H10	1.8708270	0.5584895	-2.8067232
24 H	H11	2.2114410	2.9093440	-3.5221136
25 H	H13	2.8493691	4.0536239	0.5756706
26 H	H14	2.6908176	4.6681773	-1.8297111
27 C	C18	3.1126809	-2.6139773	0.4941555
28 C	C17	4.5718111	-0.8403172	-0.0758049
29 C	C19	7.3823515	-0.8240808	0.0220874
30 C	C20	5.2825289	0.2858696	-0.5054194
31 C	C21	5.3116508	-1.9610039	0.3825548
32 C	C22	6.6996485	-1.9709129	0.4445004
33 C	C23	6.6810288	0.2864062	-0.4517809
34 H	H12	4.7658238	1.1592787	-0.8813033
35 H	H16	7.2394005	-2.8422870	0.8031504
36 H	H19	7.2243410	1.1663288	-0.7848432
37 H	H20	8.4682671	-0.8049551	0.0603636
38 N	N1	4.4353280	-2.9898728	0.7213717
39 O	O1	2.1558612	-3.3571619	0.7047751
40 C	C24	-1.6570055	-1.4326722	-0.4856866
41 H	H3	-1.3678390	-2.4498026	-0.7285367
42 C	C25	-3.0067076	-1.2188834	-0.3610849
43 S	S2	-3.8289931	0.2965835	0.0856751
44 N	N2	-3.9552741	-2.2201267	-0.5657056
45 C	C28	-5.4056063	-0.4965866	0.0232545
46 C	C29	-7.6715361	-2.0719418	-0.1685717
47 C	C30	-5.2809087	-1.8489257	-0.3379133
48 C	C31	-6.6464162	0.0744996	0.2814167
49 C	C32	-7.7884105	-0.7248870	0.1819317
50 C	C33	-6.4257919	-2.6472847	-0.4296563
51 H	H24	-6.7232541	1.1221317	0.5571400
52 H	H25	-8.7653059	-0.2952810	0.3815503
53 H	H26	-6.3636334	-3.6978995	-0.6913610

54	H	H27	-8.5612511	-2.6909257	-0.2392004
55	C	C27	-3.5512194	-3.5574539	-0.9671460
56	H	H4	-4.4349384	-4.1549775	-1.1840105
57	H	H15	-2.9754678	-4.0498494	-0.1744439
58	H	H22	-2.9365638	-3.5113065	-1.8724216
59	Cl	Cl1	-1.6510149	5.2124430	1.2239936
60	C	C26	4.8122817	-4.2917678	1.2208063
61	H	H8	5.4503633	-4.8205169	0.5017391
62	H	H17	3.8915125	-4.8572399	1.3740705
63	H	H18	5.3510297	-4.2074333	2.1727639

Convergence after 32 cycles

Heat of Formation: -2315.682533 a.u.

Solvation Energy SM5.4/P: -2315.69065 a.u.

6.6. Structure 2*E*,4*Z*,6*E*-10h

Cartesian Coordinates (Angstroms)

Atom		X	Y	Z
1 C	C1	0.8179305	-1.0546110	0.4227618
2 H	H2	0.9159462	-2.1283762	0.5386253
3 C	C2	-0.4786729	-0.5841472	0.3858487
4 C	C3	-0.8439753	0.7838332	-0.0705558
5 C	C4	-1.6131383	3.3164715	-0.9944967
6 C	C5	-0.2730061	1.3354697	-1.2294100
7 C	C6	-1.8108388	1.5405072	0.6124231
8 C	C7	-2.1955358	2.8015940	0.1636144
9 C	C8	-0.6504583	2.5925378	-1.6961999
10 H	H1	0.4585072	0.7583317	-1.7867391
11 H	H6	-2.2630207	1.1353408	1.5121708
12 H	H5	-2.9369995	3.3802710	0.7042595
13 H	H7	-0.2083705	3.0049182	-2.5970375
14 C	C9	2.0797825	-0.3424945	0.3605659
15 C	C10	3.2259236	-0.9982109	-0.0506687
16 C	C11	2.1525426	1.0593993	0.8714003
17 C	C12	2.2842162	3.6876236	1.8665691
18 C	C13	1.6654283	1.3672264	2.1508120
19 C	C14	2.7018322	2.0926988	0.0963362
20 C	C15	2.7616415	3.3958861	0.5863627
21 C	C16	1.7379574	2.6682540	2.6481531
22 H	H9	1.2334374	0.5751914	2.7552799
23 H	H10	3.0804898	1.8663120	-0.8962366
24 H	H11	3.1799231	4.1856632	-0.0321213
25 H	H13	1.3656722	2.8852634	3.6459046
26 H	H14	2.3350376	4.7033292	2.2496795
27 C	C18	3.1833291	-2.3168943	-0.7612648
28 C	C17	4.6468495	-0.6549762	0.0715247
29 C	C19	7.4570023	-0.6561320	0.0212329
30 C	C20	5.3598601	0.3734947	0.6965264
31 C	C21	5.3829518	-1.6982680	-0.5484068
32 C	C22	6.7721177	-1.7118017	-0.5912772
33 C	C23	6.7584423	0.3683659	0.6644205
34 H	H12	4.8401883	1.1750325	1.2061199
35 H	H16	7.3110932	-2.5203927	-1.0758692
36 H	H19	7.3046505	1.1735062	1.1478818
37 H	H20	8.5434668	-0.6423999	0.0008636
38 N	N1	4.5044526	-2.6576001	-1.0468455
39 O	O1	2.2185878	-3.0046653	-1.0857534
40 C	C24	-1.5228375	-1.4784890	0.8622871
41 H	H3	-1.1878910	-2.2171914	1.5866238
42 C	C25	-2.8679441	-1.5299340	0.6065632
43 N	N2	-3.6170659	-0.9732729	-0.4190511
44 S	S2	-3.9488028	-2.4335101	1.7171625
45 C	C28	-5.0006835	-1.1303454	-0.2882026
46 C	C29	-7.6849533	-1.6957086	0.2092375
47 C	C30	-5.3737496	-1.9229896	0.8106899
48 C	C31	-5.9771670	-0.6118951	-1.1415182
49 C	C32	-7.3190720	-0.9030817	-0.8808933
50 C	C33	-6.7086528	-2.2154336	1.0657077
51 H	H24	-5.7022447	0.0159284	-1.9826677
52 H	H25	-8.0846229	-0.4997854	-1.5373030
53 H	H26	-6.9863955	-2.8342773	1.9136429

54	H	H27	-8.7323250	-1.9105576	0.3986601
55	C	C26	-3.0467250	-0.6312456	-1.7207501
56	H	H23	-2.9946951	0.4503365	-1.8720262
57	H	H28	-3.6683903	-1.0794045	-2.5027611
58	H	H29	-2.0420560	-1.0483917	-1.7863226
59	Cl	Cl1	-2.1057068	4.9010634	-1.5837501
60	C	C27	4.8774719	-3.8736869	-1.7320926
61	H	H4	3.9541603	-4.4028472	-1.9750685
62	H	H8	5.5043676	-4.5096598	-1.0946498
63	H	H15	5.4248996	-3.6534020	-2.6567149

Convergence after 18 cycles

Heat of Formation: -2315.675481 a.u.

Solvation Energy SM5.4/P: -2315.68401 a.u.

6.7. Structure of the *N*-methyl allenyl enolate 16a

Cartesian Coordinates (Angstroms)

Atom		X	Y	Z
---------		-------------	-------------	-------------
1 C	C3	0.8627880	2.4687206	1.8917776
2 C	C4	1.3536535	4.2849486	4.0013787
3 C	C5	0.9281275	2.0314970	3.2267335
4 C	C6	1.0566365	3.8394701	1.6399382
5 C	C7	1.2954121	4.7362178	2.6821460
6 C	C8	1.1671006	2.9264500	4.2679606
7 H	H1	0.7947252	0.9739837	3.4323680
8 H	H6	0.9759937	4.2200829	0.6249048
9 H	H5	1.4231193	5.7929590	2.4611084
10 H	H7	1.2125087	2.5604670	5.2906779
11 C	C11	-1.2622225	-1.6016771	1.3790065
12 C	C12	-1.0006553	-4.0675511	2.7130732
13 C	C13	-0.8742007	-1.6519576	2.7268607
14 C	C14	-1.5315676	-2.8098596	0.7143082
15 C	C15	-1.3987967	-4.0300250	1.3739480
16 C	C16	-0.7422704	-2.8732965	3.3880578
17 H	H9	-0.7086380	-0.7189565	3.2570230
18 H	H10	-1.8481632	-2.7746498	-0.3231821
19 H	H11	-1.6085138	-4.9548724	0.8422035
20 H	H13	-0.4495167	-2.8914620	4.4351446
21 H	H14	-0.9023902	-5.0197148	3.2282957
22 C	C17	-3.7955609	-0.4887031	-0.2552599
23 C	C19	-6.5208579	-0.9501512	-0.7819640
24 C	C20	-4.5825509	-1.1806741	0.6757887
25 C	C21	-4.4200062	-0.0160944	-1.4459271
26 C	C22	-5.7638151	-0.2394363	-1.7223693
27 C	C23	-5.9343559	-1.4091503	0.4035597
28 H	H12	-4.1469300	-1.5371087	1.6045115
29 H	H16	-6.2173736	0.1281622	-2.6392846
30 H	H19	-6.5391800	-1.9502018	1.1272854
31 H	H20	-7.5735907	-1.1393590	-0.9756908
32 N	N1	-3.4717413	0.6675739	-2.1961950
33 C	C43	2.2457629	-0.4270943	-1.9020376
34 C	C28	3.5882710	-1.1232450	-1.7933452
35 C	C29	6.1567406	-1.9909766	-1.1377501
36 C	C30	4.3459948	-0.4893508	-0.8058743
37 C	C31	4.1249803	-2.2150478	-2.4623404
38 C	C32	5.4170827	-2.6434614	-2.1291451
39 C	C33	5.6285091	-0.8925001	-0.4517835
40 H	H24	3.5575813	-2.7326407	-3.2309986
41 H	H25	5.8493340	-3.4946098	-2.6469508
42 H	H26	6.2014354	-0.3870548	0.3189567
43 H	H27	7.1557090	-2.3396267	-0.8931323
44 C	C27	4.1106328	1.4450146	0.7813013
45 H	H4	3.3469660	2.1446557	1.1146473
46 H	H15	4.9821190	1.9911655	0.4051586
47 H	H22	4.4156657	0.8272757	1.6309642
48 C	C26	2.0089748	0.2027342	-3.3013926
49 H	H23	1.0272745	0.6843726	-3.3293967
50 H	H28	2.0266771	-0.5907538	-4.0561846
51 H	H29	2.7849709	0.9323165	-3.5568887
52 C	C44	1.0631050	-1.3765769	-1.5772075

53	H	H30	1.0552330	-2.1821521	-2.3193475
54	H	H31	0.1212522	-0.8290203	-1.6404602
55	H	H33	1.1541795	-1.8230311	-0.5836134
56	H	H8	1.5398297	4.9835682	4.8125552
57	C	C1	2.3877538	0.6714825	-0.8586131
58	C	C2	1.3762243	1.6922579	-0.5618523
59	H	H17	0.6140752	1.6990300	-1.3464166
60	H	H18	1.8614825	2.6715564	-0.5129426
61	C	C9	0.5693543	1.4982650	0.7959812
62	C	C24	-1.3556019	-0.3003791	0.6434883
63	C	C18	-2.4152593	-0.0654453	-0.3017667
64	C	C10	-2.2299994	0.6676446	-1.5367374
65	O	O1	-1.2063210	1.1750357	-2.0560598
66	C	C25	-0.3952109	0.6156822	0.8601138
67	N	N2	3.5823683	0.5821613	-0.2706073
68	C	C34	-3.6787806	1.2608419	-3.4932703
69	H	H2	-4.4607466	2.0307052	-3.4594927
70	H	H3	-3.9661646	0.5074086	-4.2392642
71	H	H21	-2.7327289	1.7183282	-3.7898957

Convergence after 18 cycles

Heat of Formation: -1575.763902 a.u.

Solvation Energy SM5.4/P: -1575.78503 a.u.

6.8. Structure of the *N*-tosyl allenyl enolate 16b

Cartesian Coordinates (Angstroms)

Atom		X	Y	Z
---------		-------------	-------------	-------------
1 C	C3	-1.3586072	-1.3911617	2.8575581
2 C	C4	-1.4574849	-2.5646450	5.4281260
3 C	C5	-1.7875201	-0.6559112	3.9771857
4 C	C6	-0.9851482	-2.7323993	3.0590947
5 C	C7	-1.0302656	-3.3101574	4.3286575
6 C	C8	-1.8336627	-1.2320735	5.2447124
7 H	H1	-2.0898199	0.3771141	3.8363519
8 H	H6	-0.6088908	-3.3217731	2.2269734
9 H	H5	-0.7187259	-4.3436343	4.4576887
10 H	H7	-2.1698569	-0.6386854	6.0913581
11 C	C11	-1.0492507	2.8640283	1.0094010
12 C	C12	-2.6310327	5.1876511	1.2017562
13 C	C13	-1.8171174	3.1198812	2.1560817
14 C	C14	-1.0741130	3.8048528	-0.0345034
15 C	C15	-1.8599170	4.9521166	0.0606594
16 C	C16	-2.6032830	4.2678455	2.2515554
17 H	H9	-1.7687702	2.4201973	2.9849894
18 H	H10	-0.4714306	3.6246668	-0.9190269
19 H	H11	-1.8670315	5.6667810	-0.7586977
20 H	H13	-3.1843207	4.4500104	3.1522956
21 H	H14	-3.2382670	6.0861496	1.2760398
22 C	C17	2.0635226	2.5945342	0.2079052
23 C	C19	4.3831337	4.1306546	-0.1409974
24 C	C20	2.2207647	3.7974701	0.9124350
25 C	C21	3.1094562	2.1743862	-0.6591185
26 C	C22	4.2623048	2.9283544	-0.8474848
27 C	C23	3.3764125	4.5566287	0.7316542
28 H	H12	1.4476571	4.1309408	1.5977344
29 H	H16	5.0387070	2.5954219	-1.5221815
30 H	H19	3.4938717	5.4897839	1.2771986
31 H	H20	5.2769056	4.7341015	-0.2758299
32 N	N1	2.7102081	0.9277964	-1.2099260
33 C	C43	-2.8686202	-0.6472536	-1.8828010
34 C	C28	-4.3558507	-0.4814557	-2.1179153
35 C	C29	-7.1359230	-0.3498854	-1.9501451
36 C	C30	-5.0408340	-0.8829078	-0.9701176
37 C	C31	-5.0762077	-0.0043420	-3.2052284
38 C	C32	-6.4728534	0.0556973	-3.1131141
39 C	C33	-6.4239004	-0.8309872	-0.8464558
40 H	H24	-4.5705440	0.3184029	-4.1110445
41 H	H25	-7.0491589	0.4244187	-3.9565782
42 H	H26	-6.9371877	-1.1424611	0.0574318
43 H	H27	-8.2189045	-0.2908182	-1.8986576
44 C	C27	-4.4902697	-1.8134164	1.3052923
45 H	H4	-3.6311234	-2.2114693	1.8405828
46 H	H15	-5.2473278	-2.5922344	1.1793238
47 H	H22	-4.9131472	-0.9853389	1.8817548
48 S	S1	3.5565389	-0.0595776	-2.3092784
49 C	C36	3.9014539	-1.5495095	-1.3734559
50 C	C37	4.4784190	-3.8723364	0.0728740
51 C	C38	3.0374648	-2.6395029	-1.4567218
52 C	C39	5.0531615	-1.6000174	-0.5839728

53	C	C40	5.3299326	-2.7583808	0.1358431
54	C	C41	3.3366579	-3.7941304	-0.7340666
55	H	H32	2.1491415	-2.5700118	-2.0715228
56	H	H35	5.7219641	-0.7467591	-0.5463378
57	H	H36	6.2258264	-2.8011379	0.7510589
58	H	H37	2.6696494	-4.6508505	-0.7983231
59	C	C42	4.7990623	-5.1265555	0.8512497
60	H	H38	5.7417630	-5.5745719	0.5125928
61	H	H39	4.0127092	-5.8792467	0.7370301
62	H	H40	4.9107112	-4.9144763	1.9215994
63	O	O2	2.6715607	-0.3995770	-3.4201572
64	O	O3	4.8343770	0.6126877	-2.5592119
65	C	C26	-2.2040007	-1.6393660	-2.8735747
66	H	H23	-1.1343269	-1.7215869	-2.6621778
67	H	H28	-2.3174411	-1.2527955	-3.8914843
68	H	H29	-2.6663435	-2.6313891	-2.8304460
69	C	C44	-2.1161800	0.7104025	-1.9472032
70	H	H30	-2.2188803	1.1098439	-2.9614291
71	H	H31	-1.0563493	0.5584895	-1.7349735
72	H	H33	-2.5285105	1.4381604	-1.2441218
73	H	H8	-1.4942239	-3.0150856	6.4161703
74	C	C1	-2.8463884	-1.2216275	-0.4757807
75	C	C2	-1.6336067	-1.6274932	0.2610794
76	H	H17	-0.7683505	-1.5694202	-0.4032707
77	H	H18	-1.7663689	-2.6623482	0.5945802
78	C	C9	-1.2774064	-0.7441428	1.5149484
79	C	C24	-0.2589071	1.6002704	0.8691142
80	C	C18	1.0190763	1.5964928	0.1885352
81	C	C10	1.4285296	0.5363676	-0.6757714
82	O	O1	0.8773121	-0.5300339	-1.0202842
83	C	C25	-0.7953102	0.4627372	1.3300352
84	N	N2	-4.0848147	-1.3276352	-0.0116613

Convergence after 98 cycles

Heat of Formation: -2355.382800 a.u.

Solvation Energy SM5.4/P: -2355.41676 a.u.

6.9. HOMO and LUMO of 2*Z*,4*Z*-8a

LUMO -1.92911 eV

HOMO -4.66131 eV

6.10. TD-DFT calculation of 2*E*,4*Z*,6*Z*-10a

Computed xyz-Coordinates for 2*E*,4*Z*,6*Z*-10a

The ground state geometry of 2E,4Z,6Z-**10a** Trien EZZ was optimized in a DFT calculation with the B3LYP functional and the 6-311G(d,p) basis set in the program package Gaussian09. The minima structure was confirmed by analytical frequency analysis. Computational details of the calculated **2*E*,4*Z*,6*Z*-10a**:

XYZ-coordinates for **10a**:

C	-0.336926	0.458955	0.154811
C	-1.723133	0.506568	0.071686
C	-2.479604	1.725742	-0.319862
C	-3.970849	3.975620	-1.104981
C	-2.127193	2.456868	-1.462959
C	-3.591423	2.146022	0.425500
C	-4.324846	3.264639	0.041957
C	-2.869984	3.566418	-1.856335
C	0.644841	1.497764	0.225216
C	1.987882	1.223881	-0.037129
C	0.272430	2.862011	0.705235
C	-0.349763	5.423529	1.665366
C	-0.305307	3.027881	1.969372
C	0.528792	3.997174	-0.074428
C	0.212309	5.266711	0.397664
C	-0.605012	4.300921	2.450557
C	2.424375	0.020386	-0.762848
C	3.198889	1.972473	0.296407
C	5.813413	2.900638	0.702578
C	3.429682	3.118276	1.068146
C	4.319583	1.290154	-0.236589
C	5.619735	1.743363	-0.054878
C	4.730939	3.574746	1.265923
N	3.843438	0.134541	-0.916968
O	1.788424	-0.927330	-1.194565
C	-2.433667	-0.695354	0.439634
C	-3.695626	-1.175726	0.181892
N	-4.593067	-0.828133	-0.796156
C	-4.318602	-2.304568	1.034761
C	-5.813333	-1.508193	-0.626908
C	-7.980932	-3.082597	0.051012
C	-5.713596	-2.397160	0.445644
C	-6.980650	-1.382917	-1.373360
C	-8.066842	-2.188097	-1.014776
C	-6.793806	-3.193129	0.787723

C	-4.354756	-1.925520	2.528695
S	4.761671	-1.114870	-1.653582
C	4.621768	-2.495646	-0.528984
C	4.434239	-4.649401	1.232145
C	3.606509	-3.433534	-0.705797
C	5.545290	-2.610910	0.510135
C	5.441940	-3.687625	1.382578
C	3.523441	-4.503519	0.178512
C	4.351863	-5.829877	2.165897
O	4.121699	-1.447673	-2.917254
O	6.144864	-0.647655	-1.646786
C	-3.550068	-3.631867	0.835375
C	-4.294946	-0.104714	-2.024487
H	0.073137	-0.540875	0.205843
H	-1.282178	2.132688	-2.058853
H	-3.870786	1.598059	1.317567
H	-5.173515	3.581745	0.637586
H	-2.589434	4.111106	-2.750793
H	-4.547571	4.841546	-1.409711
H	-0.506184	2.155880	2.580720
H	0.975517	3.879444	-1.054888
H	0.405906	6.134931	-0.222149
H	-1.040015	4.414365	3.437172
H	-0.588669	6.413774	2.036368
H	2.604957	3.652076	1.516140
H	6.456536	1.215392	-0.483305
H	4.898276	4.463583	1.863395
H	6.821766	3.268258	0.853838
H	-1.869195	-1.342495	1.103269
H	-7.059910	-0.684390	-2.196639
H	-8.990606	-2.108058	-1.576301
H	-6.728366	-3.893747	1.613095
H	-8.836300	-3.694227	0.312540
H	-3.343415	-1.808047	2.924488
H	-4.855669	-2.709854	3.101903
H	-4.895754	-0.989639	2.683736
H	2.894795	-3.316026	-1.510077
H	6.335213	-1.880519	0.626474
H	6.157873	-3.783021	2.191327
H	2.735124	-5.236319	0.046715
H	4.955111	-6.661528	1.786390
H	3.325572	-6.188339	2.264999
H	4.729358	-5.578358	3.158871
H	-2.528700	-3.551966	1.214845
H	-3.506320	-3.906693	-0.220581
H	-4.052844	-4.436005	1.378755
H	-4.610547	0.938409	-1.974378
H	-4.810777	-0.596325	-2.851338
H	-3.223939	-0.142814	-2.209591

SCF Done: E(RB3LYP) = -2355.95986296 A.U. after 14 cycles

Sum of electronic and zero-point Energies= -2355.288329

Sum of electronic and thermal Energies= -2355.246284

Sum of electronic and thermal Enthalpies= -2355.245340

Sum of electronic and thermal Free Energies= -2355.367249

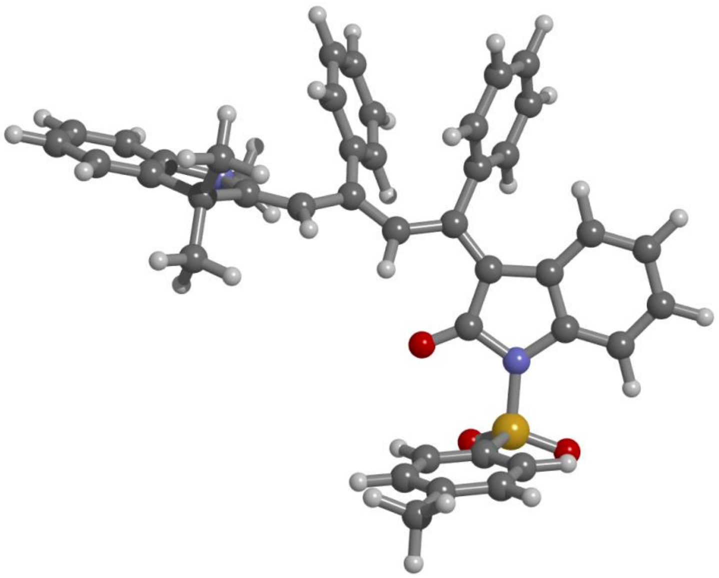

Figure S1: Optimized geometry of **2*E*,4*Z*,6*Z*-10a** at the B3LYP 6-311G(d,p) level of theory.

Computed UV–vis spectrum of calculated compound 10a by TD-DFT

The optimized structure was used in a TD-DFT calculation using the hybrid exchange-correlation functional CAM-B3LYP.

First four dominant transitions of the first excited state for **10a** regarding to the oscillatory strength:

1) 541 nm, oscillator strength: 1.4722, orbitals involved: HOMO → LUMO
2) 345 nm, oscillator strength: 0.2141, orbitals involved: HOMO-1 → LUMO, HOMO → LUMO+2
3) 286 nm, oscillator strength: 0.1454, orbitals involved: HOMO-11 → LUMO, HOMO-2 → LUMO, HOMO-1 → LUMO, HOMO → LUMO+1, HOMO → LUMO+2
4) 265 nm, oscillator strength: 0.0994, orbitals involved: HOMO-5 → LUMO, HOMO-2 → LUMO+6, , HOMO-1 → LUMO+5, HOMO-1 → LUMO+5, HOMO → LUMO+5, HOMO → LUMO+6

---

1 *Organikum*, 22nd ed.; Becker, H. G. O.; Beckert, R.; Domschke, G.; Fanghänel, E.; Habicher, W. D.; Metz, P.; Pavel, D.; Schwetlick, K. Wiley-VCH, Weinheim, New York, Chichester, Brisbane, Singapore, Toronto, **2004**.

2 D´Souza, D. M.; Kiel, A.; Herten, D. P.; Müller, T. J. J. *Chem. Eur. J.* **2008**, *14*, 529-547.
